# The Role of Extracellular Loops in the Folding of Outer Membrane Protein X (OmpX) of *Escherichia coli*

**DOI:** 10.3389/fmolb.2022.918480

**Published:** 2022-07-14

**Authors:** Simen Hermansen, David Ryoo, Marcella Orwick-Rydmark, Athanasios Saragliadis, James C. Gumbart, Dirk Linke

**Affiliations:** ^1^ Section for Genetics and Evolutionary Biology, Department of Biosciences, University of Oslo, Oslo, Norway; ^2^ Interdisciplinary Bioengineering Graduate Program, Georgia Institute of Technology, Atlanta, GA, United States; ^3^ School of Physics, Georgia Institute of Technology, Atlanta, GA, United States

**Keywords:** outer membrane protein, protein folding, membrane insertion, beta-barrel, ompx, bam complex, extracellular loops

## Abstract

The outer membrane of Gram-negative bacteria acts as an additional diffusion barrier for solutes and nutrients. It is perforated by outer membrane proteins (OMPs) that function most often as diffusion pores, but sometimes also as parts of larger cellular transport complexes, structural components of the cell wall, or even as enzymes. These OMPs often have large loops that protrude into the extracellular environment, which have promise for biotechnological applications and as therapeutic targets. Thus, understanding how modifications to these loops affect OMP stability and folding is critical for their efficient application. In this work, the small outer membrane protein OmpX was used as a model system to quantify the effects of loop insertions on OMP folding and stability. The insertions were varied according to both hydrophobicity and size, and their effects were determined by assaying folding into detergent micelles *in vitro* by SDS-PAGE and *in vivo* by isolating the outer membrane of cells expressing the constructs. The different insertions were also examined in molecular dynamics simulations to resolve how they affect OmpX dynamics in its native outer membrane. The results indicate that folding of OMPs is affected by both the insert length and by its hydrophobic character. Small insertions sometimes even improved the folding efficiency of OmpX, while large hydrophilic inserts reduced it. All the constructs that were found to fold *in vitro* could also do so in their native environment. One construct that could not fold *in vitro* was transported to the OM *in vivo*, but remained unfolded. Our results will help to improve the design and efficiency of recombinant OMPs used for surface display.

## 1 Introduction

The cell envelope of Gram-negative bacteria consists of two lipid bilayers separated by a peptidoglycan cell wall that keeps the cell stable against osmotic pressure. The bilayer that surrounds the cytoplasm is the inner membrane (IM) and is primarily composed of phospholipids. The bilayer that is exposed to the environment is the outer membrane (OM). It is asymmetric, where the outer leaflet consists of lipopolysaccharides and the inner leaflet consists of phospholipids. Outer membrane proteins (OMPs) are transmembrane β-barrel proteins in the outer membrane of Gram-negative bacteria. OMPs are essential to the viability of Gram-negative bacteria, and fulfil a variety of key functions, from nutrient transport to cell division (Koebnik et al., 2000).

The transmembrane domain of OMPs consists of an amphipathic β-sheet that is rolled up into a barrel-shaped structure (Fairman et al., 2011; Hermansen et al., 2022). This architecture is fundamentally different from the hydrophobic, helical transmembrane domains of other membrane proteins, and occurs only in proteins of the Gram-negative outer membrane, and of evolutionarily related membranes in eukaryotes such as the outer membranes of mitochondria and chloroplasts (Ulrich and Rapaport, 2015). In Gram-negative bacteria, OMPs have an even number of β-strands, where the N and C-terminus of the domain reside on the periplasmic side of the OM; this is based on the fact that ββ-hairpins are the repeating unit in OMP evolution (Arnold et al., 2007; Remmert et al., 2010). Based on this principle, known OMPs can have a varying barrel size with strand numbers ranging from eight to over 30 (Hermansen et al., 2022), where eight strands seem to be the minimal size to form a closed barrel in the membrane. The core transmembrane domain can be further decorated with other domains. These can either be inserted into periplasmic turns or extracellular loops, or can be attached to the N- or C-terminus. Examples for 8-stranded barrels with additional domains include OmpA and its homologues in diverse organisms that harbor a C-terminal peptidoglycan-binding domain (Grizot and Buchanan, 2004), or SabA from *Helicobacter pylori* that harbors an extracellular insertion between strands 1 and 2 (Coppens et al., 2018).

The extracellular loops and the periplasmic turns of OMPs have been subject to a wide variety of studies to examine their biological function. Loops of OMPs have been found to be involved in pathogenesis (Maruvada and Kim, 2011), they are essential for the function of the core component of the BAM complex, BamA (Browning et al., 2013), and *e.g*. for the proteolytic activity of the outer membrane protease OmpT (Hritonenko and Stathopoulos, 2007). The more indirect functions of loops on the structure and stability of OMPs have also been demonstrated. In the trimeric porin OmpF, loop three is folded into the core of the ß-barrel where it restricts the diameter of the channel, and loop two stabilizes trimerization interactions (Phale et al., 1998). In contrast to these very specific examples where extracellular loops participate directly or indirectly in protein function, OMP loops often do not appear to be essential for structural stability. When all the extracellular loops of OmpA were shortened, the mutated protein could still fold into the native β-barrel *in vivo* (Koebnik, 1999). Similarly, an insertion of 21 residues into loops two and three of OmpA did not interfere with membrane assembly *in vivo* (Freudl, 1989). In general, most structural studies indicate that the extracellular loops are modifiable without compromising the stability of the ß-barrel. This feature makes the loops of OMPs an attractive target for genetic modifications, with several possible biotechnological applications (Parwin et al., 2019). Loop modifications have been successfully used for surface display of epitopes (Lång, 2000; Rice et al., 2006), for the bio-adsorption of metals (Xu and Lee, 1999), and for the display of trypsin cleavage sites (Koebnik and Braun, 1993; Ried et al., 1994), all without causing any significant perturbations to the ß-barrel structure.

OmpX belongs to a family of small integral membrane proteins in Gram-negative bacteria of mostly unknown function. The protein was first characterized in *Enterobacter cloacae* (Stoorvogel et al., 1991) and later in *Escherichia coli* (Mecsas et al., 1995). The extracellular loops show a high degree of sequence variation between homologues, while the barrel domain is more conserved (Yamashita et al., 2011). With the signal peptide cleaved off, OmpX in *E. coli* has a molecular weight of 16.5 kDa, and with just eight transmembrane β-strands, the protein is one of the smallest characterized OMPs. Several functions have been attributed to OmpX, such as regulation of surface adhesion (Otto and Hermansson, 2004) and serum resistance (Lin et al., 2002), but no detailed mechanisms have been described that explain how the protein could carry out these possible functions. It is worth noting though that the OmpX homologue Ail from different *Yersinia* species plays a direct role in virulence (Kolodziejek et al., 2012).

The crystal structure of *E. coli* OmpX revealed that the extracellular loops two and three jointly form a four-stranded β-sheet that extends from the β-barrel into the extracellular space (Vogt and Schulz, 1999). It is not known if this β-sheet forms *in vivo* where the loops would be in contact with the LPS layer of the OM. Regardless of its (unclear) biological function, OmpX has been used extensively as a model system to study the folding (Michalik et al., 2017; Rath et al., 2019), evolution (Arnold et al., 2007; Zhang et al., 2021), and lipid interactions of OMPs (Fernández et al., 2002; Maurya et al., 2013; Chaturvedi and Mahalakshmi, 2018). As a model system, OmpX is ideal because of its relatively simple structure and low toxicity when expressed at high levels in *E. coli*. It can be produced in large quantities in inclusion bodies, and readily refolds *in vitro* with the help of detergents or lipids (Arnold et al., 2007; Rath et al., 2019).

In this study, we used OmpX as a model system to systematically assess how tolerant the extracellular loops of OMPs are to different insertions.

## 2 Materials and Methods

### 2.1 Cloning and Strain Generation

Variants of OmpX from *E. coli* containing loop inserts were generated by blunt-end ligation of PCR products. A pET3b plasmid containing ompX without the signal peptide, a T7 promotor and ampicillin resistance (Pautsch et al., 1999) or an identical plasmid containing the complete OmpX sequence (with signal peptide (Arnold et al., 2007)) was used as a template for the PCR. Primers were designed to amplify the plasmid with overhangs containing the intended insert. Primers are listed in Supplementary Material S1 (Supplementary Table S1). Ligation products were transformed into calcium-competent *E. coli* TOP10 cells (Invitrogen) and the DNA sequence of the inserts was confirmed with Sanger sequencing (LightRun, Eurofins).

For *in vivo* studies of OmpX folding, a variant of *E. coli* BL21 had to be generated that lacked the wildtype OmpX gene. This was done using P1 transduction as described (Saragliadis et al., 2018), with *E. coli* BL21 as the acceptor strain and an OmpX knockout from the Keio collection (Baba et al., 2006) as the donor strain. This gave rise to the kanamycin-resistant strain *E. coli* BL21ΔOmpX. Absence of the *ompX* gene was verified by Western blotting of whole-cell lysates using an OmpX rabbit antiserum (Arnold et al., 2007) and *E. coli* BL21 as a positive control (data not shown).

### 2.2 Expression of Inclusion Bodies

Loop variants of OmpX were expressed as inclusion bodies by introducing the plasmids into *E. coli* C41 (DE3) (Miroux and Walker, 1996). The cells were grown at 37°C in 500 ml LB medium to an optical density of 0.5 before adding 1 mM IPTG for induction of expression. Cultures were then incubated overnight at room temperature and harvested by centrifugation.

Cell pellets were resuspended in PBS with DNAse (0.1 mg/ml) and lysozyme (0.1 mg/ml). Inclusion bodies were released by lysing the cells in a French pressure cell, following published procedures (Arnold et al., 2007). In brief, inclusion bodies were collected by centrifugation (4,000 x *g*, for 10 min) and washed by resuspending the pellet in detergent solution (1% (v/v) Triton-X100 in 50 mM Tris-HCl pH 8.0). Residual detergent was washed away by repeated centrifugation and resuspension of the inclusion bodies in Tris-HCl buffer (three times).

Inclusion bodies were dissolved in urea buffer (8 M, 50 mM mM Tris-HCl, pH 8.0) at room temperature. Insoluble debris was removed by centrifugation (12,000 x *g*, for 10 min) and the crude fractions of proteins were diluted to a concentration of 1.4 mg/ml. Protein concentration was estimated by measuring absorbance at 280 nm with a BioPhotometer (Eppendorf). The extinction coefficient of OmpX was determined to be 34,840 M^−1^cm^−1^ with ProtParam (Gasteiger et al., 2005). These crude protein fractions were relatively pure and were suitable for SDS-PAGE heat-modifiability assays without requiring further purification steps. As an added benefit, this minimized the amount of time the protein remained diluted in urea before folding. Prolonged exposure in urea can cause carbamoylation, an irreversible modification of primary amines (Kollipara and Zahedi, 2013) that might negatively impact folding assays.

### 2.3 *In vitro* Protein Folding Assays

Folding of denatured protein was initiated by diluting the crude protein fractions 1/20 (v/v) in detergent buffer (1% (w/v) sulfobetaine 12, 50 mM Tris-HCl, pH 8.0). The folding was performed at 15 °C on a PCMT Thermo-Shaker at 1000rpm for up to 64 min 10uL of the sample was quenched in 10uL of ice-cold SDS-PAGE sample buffer (8% SDS, 40% Glycerol, 50 mM Tris/HCl pH 6.8, 2 mM EDTA, 0.1% bromophenol blue) at specific time points. Three technical replicates were produced for each data point of the folding kinetics experiments.

Samples were kept at 4°C and applied to a pre-cast Novex WedgeWell 4–20% Tris-Glycine polyacrylamide gel (Invitrogen) along with an unfolded control and ladder (PageRuler™ Plus Prestained Protein Ladder, 10–250 kDa). A VWR 250V power source and a xCell SureLock™ Electrophoresis Cell (Invitrogen) was used for electrophoresis at a constant 225V in Tris-glycine buffer. The electrophoresis chamber was kept on ice for the duration of the electrophoresis. Ensuring consistent voltage, current and temperature across all sampled gels was essential to measure reproducible folding kinetics. The gels were stained in 0.125% Coomassie Brilliant Blue R250 (SIGMA) in 50% Ethanol, 10% Acetic acid overnight, and destained in distilled water.

### 2.4 Gel Densitometry and Folding Kinetics

The gels were imaged with a GelDoc XR + Molecular Imager on a white light conversion screen (Bio-Rad). The lower range of the frequency distribution histogram of the image was removed and the gel images were converted to 16-bit greyscale with the Image Lab software (Bio-Rad). Gel densitometry was done by measuring the integrated density of the separated protein bands with ImageJ (Schindelin et al., 2012). Background noise was removed with rolling ball background subtraction with a ball radius of 50 pixels. The fraction of folded protein at given time point was calculated by dividing the integrated density of the folded protein fraction by the sum of the folded and unfolded fraction.

The folding kinetics were modeled by non-linear least-square regression. The folding kinetics of OMPs at high concentrations of detergent, lipid or amphipol to protein can be described by a single exponential function (Kleinschmidt, 2006). The non-linear function used for the modeling thus contained only two variables, for folding rate *(k)* and yield *(A)* (function 1).

Function 1: $XF\left(t\right)=A\times \left(1-e^{-kt}\right)$ where XF is the fraction of folded protein at time t.

It is worth noting that folding protocols that involve rapid dilution of OMPs will cause some protein to aggregate, leading to folding yields <100%. Outside of optimal folding temperatures this effect becomes more pronounced (Maurya et al., 2013). It is thus critical to perform folding experiments at highly controlled temperature conditions.

### 2.5 *In vivo* Expression and Membrane Fractionation

For *in vivo* expression, cultures of *E. coli* BL21ΔOmpX harboring the different plasmids were grown in autoinduction medium (Studier, 2005) over night, after finding that strong IPTG induction was detrimental to membrane insertion. Cultures were diluted to an OD_600_ of 1.0, and 40 ml were harvested by centrifugation and resuspended in 1.5 ml HEPES buffer (pH 7.4, 10 mM MgCl_2_, 4°C) with DNase (0.1 mg/ml, Sigma-Aldrich) and lysozyme (0.1 mg/ml, AppliChem). The cells were incubated for 15 min on ice and then transferred to a 2 ml Micro tube with 250 μL Zirconia/Silica Beads (0.1 mm dia, BioSpec Products). Cells were then lysed with a FastPrep™ FP120 cell disruptor (three times for 40 s at 6.5 m/s). Cells were cooled for 2 minutes on ice between runs. Intact bacteria, silica beads and cell debris were removed by a brief centrifugation step (12,000 rcf, 1 min). The resulting supernatant was centrifuged (16,000 rcf, 30 min) to pellet the membranes. The IM fraction of the membrane pellet was solubilized selectively (Thein et al., 2010) by resuspending the membranes in 200 μL HEPES (pH 7.4) and then adding 200 μL of 2% N-lauryl Sarcosine (Sigma-Aldrich) for 30 min on a VWR Tube Rotator at room temperature. The remaining OM was harvested by centrifugation (16,000 rcf, 30 min). The OM pellet was washed twice with 500 μL HEPES buffer (pH 7.4), and then re-centrifuged as above. After washing, the pellet was resuspended in 60 μL HEPES buffer (pH 7.4). 30 μL of the suspension was incubated in 500 μL of 6M urea (100 mM glycine, 80 mM HEPES, pH 7.4) for 30 min on a VWR Tube Rotator to remove protein only peripherally attached, rather than integrated into the OM. Urea-washed membrane fractions were then centrifuged (16,000 rcf, 30 min) and resuspended in 30 μL HEPES (pH 7.4). The OM preparations were then mixed with 10 μL 4x SDS buffer for SDS-PAGE analysis.

### 2.6 Stress Signals in the *E. coli* Outer Membrane

To assess *in vivo* stress responses induced by the expression of different OmpX variants, we utilized a reporter gene assay as described previously (Steenhuis et al., 2020; Steenhuis et al., 2021). The *E. coli* strain C41 was used because it has been demonstrated in the past to be robust towards the over-expression of membrane proteins and to associated toxic effects of protein over-expression (Miroux and Walker, 1996). Chemically competent C41 cells were transformed with the reporter plasmids (kindly provided by Joen Luirink). Individual colonies were used to inoculate M9 minimal medium containing ampicillin and kanamycin at 100 and 50 μg/ml respectively in order to maintain reporter plasmids and the plasmids encoding for our OmpX variants. For a list of plasmids used in this study see Supplementary Material S1 (Suppplementary Table S2). Cultures containing combinations of two plasmids (one reporter and one OmpX construct) were grown overnight at 37°C with shaking (200 rpm) in a Minitron shaker incubator (Infors HT, Switzerland). Overnight cultures were diluted 1:40 in fresh M9 medium supplemented with antibiotics and were grown as before until an optical density OD_600_ of 0.4–0.5 was reached. At this point, all bacterial cultures were diluted to OD_600_ of 0.1 and 80 µL were dispensed in a microtiter 96-well plate (655,090, Greiner Bio-One) containing 80 µL of either M9 with antibiotics or M9 with antibiotics and a positive control stress response compound or isopropyl β-d-1-thiogalactopyranoside (IPTG, R0392 Thermo Scientific) at 2 mM. As a positive control polymyxin B (PMB) or ethanol was used as described (Steenhuis et al., 2020). The plates were sealed (Breathe-Easy, Z380059 Sigma-Aldrich), and were incubated at 37°C with linear (3 mm) shaking at 567 cpm in a plate reader (Synergy H1, BioTek). OD_600_ and fluorescence (ex. 488 nm, em. 535 nm) were measured continuously for 3 h.

### 2.7 Modelling

#### 2.7.1 System Generation

**FIGURE 1:**
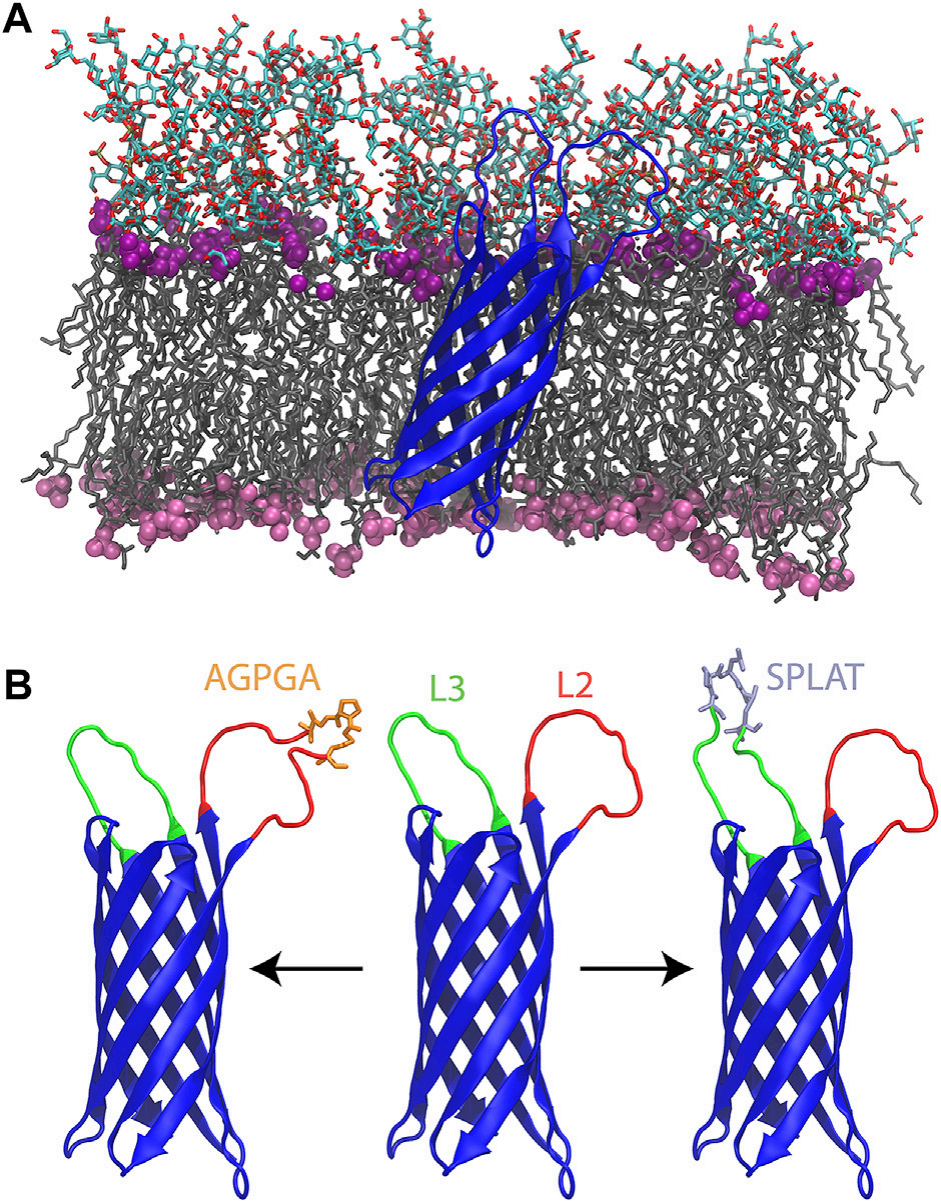
**(A)** Wildtype OmpX (blue) simulated in its native *E. coli* outer membrane. The lipid tails are shown in a grey licorice representation. The phosphate groups for LPS and phospholipids are shown as purple (LPS) and pink (phospholipid) spheres. The oligosaccharides of the LPS molecules are shown in a licorice representation colored by atom type. **(B)** From left to right, L2AGPGA1X, wildtype, and L3SPLAT1X OmpX constructs. Loops two and three are colored red and green, respectively. The AGPGA and SPLAT inserts are shown in a licorice representation, and colored orange for AGPGA and iceblue for SPLAT.

All systems were generated using CHARMM-GUI (Jo et al., 2008; Lee et al., 2019) using a previously resolved NMR structure of OmpX (PDB ID: 2M06) (Hagn et al., 2013) (see wildtype system in Figure 1A). The membrane was built primarily based on previously simulated lipid compositions, with LPS from the *E. coli* K12 strain, which has no O-antigen (Wu et al., 2014; Hwang et al., 2018). The inner leaflet of the membrane was composed of 75% PPPE, 20% PVPG, and 5% PVCL2. The system was solvated and ionized to a concentration of 0.15 M of potassium chloride (KCl). LPS was neutralized using divalent ions with magnesium (Mg^2+^) for lipid A and calcium (Ca^2+^) for the LPS core sugars.

#### 2.7.2 Molecular Dynamics (MD) Simulations

All-atom MD simulations were performed using NAMD3 (Phillips et al., 2005; Phillips et al., 2020) along with the CHARMM36 m force field for proteins (Best et al., 2012; Huang et al., 2017), the CHARMM36 force field for lipids (Klauda et al., 2010), and TIP3P water (Jorgensen et al., 1983). All simulations were performed under periodic boundary conditions with a cutoff at 12 Å for short-range electrostatic and Lennard-Jones interactions and a force-based switching function starting at 10 Å. The particle-mesh Ewald method (Darden et al., 1999) with a grid spacing of less than 1 Å was used for calculation of long-range electrostatics interactions. Bonds between a heavy atom and a hydrogen atom were maintained to be rigid, while all other bonds remain flexible. Each system was equilibrated for 500 ns in duplicate under an isothermal-isobaric ensemble (NPT) at 310 K and 1 bar, with a timestep of 4 fs with hydrogen mass repartitioning (Hopkins et al., 2015; Balusek et al., 2019). A Langevin thermostat with a damping coefficient of 1 ps^−1^ was used for temperature control and a Langevin piston with a period of 0.1 ps and decay of 0.05 ps was used for pressure control. The total simulation time was 14 µs (1 µs for each system). The simulations were visualized using VMD (Humphrey et al., 1996).

## 3 Results

### 3.1 Construct Design

To assess the contribution of the extracellular loops on the folding of OMPs, we introduced peptides of various lengths into loop 2 and loop 3 of *E. coli* OmpX. Two types peptide insertions were tested in this study. The amino acid sequence of the 5-residue inserts was SPLAT and AGPGA, respectively. The inserts do not contain charged residues, and are composed of relatively small amino acids commonly found in OMP loops; both inserts contain one proline residue per repeat. It is worth noting that overall, the extracellular loops of OMPs have less strict sequence requirements compared to the very short turns that connect OMP beta-strands on the periplasmic side of these proteins (Franklin and Slusky, 2018).

Each insert was introduced individually in loop 2 or 3, or repeatedly, leading to insertions of 5–20 residues (Table 1). Thus, with the exception of the wildtype system, all systems have between one and four copies of the sequence AGPGA or the sequence SPLAT inserted in loop 2 (residues 49–59) and/or loop 3 (residues 91–103). The locations of loops 2 and 3, along with inserts are shown in Figure 1B. Additional details of the systems used in this study are given in , Supplementary Table S3.

**TABLE 1:** Sequence and properties of the loop inserts used in this study.

Insert	Molecular Weight	pI	Hydrophobicity (Wimley-White)
SPLAT	469.54	5.24	+0.1 Kcal * mol-1
SPLATx2	939.08	5.24	+0.2 Kcal * mol-1
SPLATx4	1878.16	5.24	+0.4 Kcal * mol-1
AGPGA	353.38	5.57	+3.44 Kcal * mol-1
AGPGAx2	706.76	5.57	+6.88 Kcal * mol-1
AGPGAx4	1413.52	5.57	+13.76 Kcal * mol-1

The two types of insertion sequences are characterized by their differences in hydrophobicity, while the molecular weight and isoelectric point (pI) of the inserts are comparable. Hydrophobicity was calculated according to the Wimley-White scale (Wimley and White, 1996). Based on this, the SPLAT insert partitions into hydrophobic environments more favorably than the AGPGA insert. It is worth noting that the hydrophobicity of the SPLAT inserts is almost constant with increasing repeat number, while the hydrophobicity decreases with repeat number for the AGPGA inserts.

### 3.2 *In vitro* Folding Assays

**FIGURE 2:**
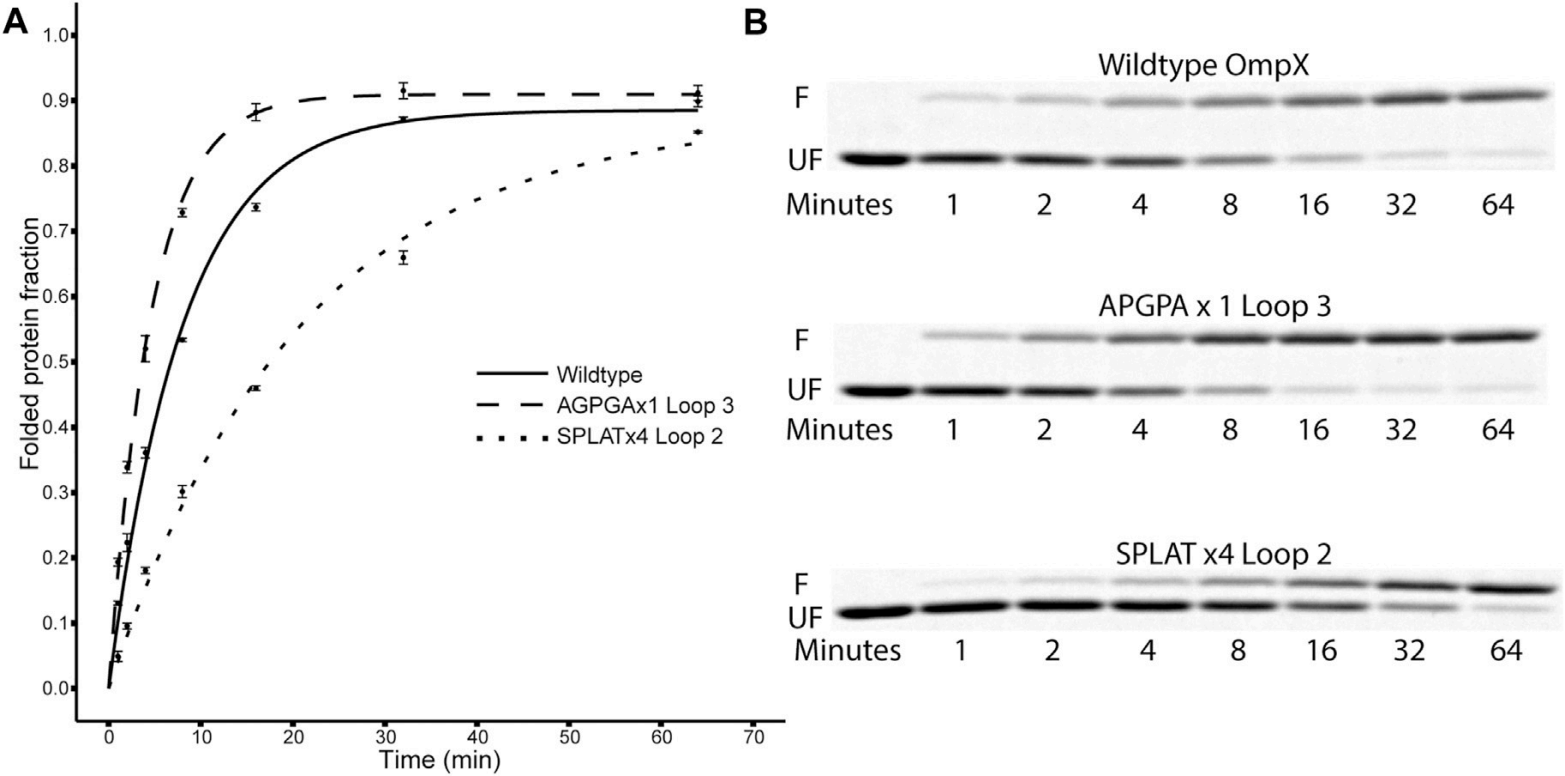
Folding kinetics of OmpX variants. **(A)** shows data points and computed fit curves for three representative datasets: wildtype OmpX, the fast-folding variant APGPAx1 L3, and the slow-folding variant SPLATx4 L2. Due to the difference in migration between folded and unfolded forms of OmpX, densitometry can be used on the SDS-PAGE gels [**(B)**, F: folded band, UF: unfolded band] to measure the ratio and estimate folding rate and yield after plotting the fraction of folded protein over time **(A)**. See Table 2 for detailed results for all constructs.

To assess the effect of different loop insertions, we performed protein folding experiments *in vitro* under strict temperature control (at 15°C). The folding protocol using the detergent SB-12 was adapted from previous work (Arnold et al., 2007). In a nutshell, OmpX and variants thereof were expressed in *E. coli* in the form of inclusion bodies, from constructs lacking the native signal peptide. The inclusion bodies contain only minor impurities, so that no chromatography steps are necessary to obtain OmpX samples of sufficient quality after cell lysis and extensive washing of the pellet. The inclusion bodies are solubilized in 8 M urea solution, and after adjusting the protein concentration, the protein is folded by direct dilution into the detergent-rich folding buffer. The folding reaction is quenched by mixing the sample with SDS sample buffer (without heating the sample). The sample is then loaded on an SDS gel, to separate folded from unfolded protein. This “gel shift assay” or “heat modifiability assay” is based on a peculiar property of bacterial outer membrane proteins: they do not easily denature in SDS (except when heated sufficiently), and the folded form has a different capacity for SDS binding, leading to differences in apparent molecular weight compared to the fully denatured form (Rosenbusch, 1974; Leo et al., 2015; Orwick-Rydmark et al., 2016). By scanning the resulting SDS-PAGE gels after Coomassie staining, densitometry can thus be used to quantify and compare the folded and unfolded form of an OMP (Figure 2). Raw 16bit greyscale TIF files and 8bit copies of the imaged gels are provided in Supplementary Material S2.

Using this methodology, we compared the folding behavior of our constructs with inserts in either loop 2, loop 3, or both. The summary of these experiments can be found in Table 2. Folding rate and folding yield were estimated from curve fits as described above and exemplified in Figure 2. All constructs used in these experiments folded well *in vitro*, except for a construct with four AGPGA inserts in both loops. We observed that the folding yield remains virtually unchanged (85–91%), independent of the insert sequence and insert position. Only the construct with 4x SPLAT insertions in both loops had a slightly lower folding yield (ca. 80%). In contrast to this, the folding rate was much more affected by the different insertions. Consistently, constructs with inserts in loop 3 folded faster than their equivalent counterparts with insertions in loop 2, and some of them even faster than the wildtype protein under the assay conditions tested here. Less surprisingly, adding multiple copies of the inserted peptide repeat consistently led to slower folding kinetics. Last but not least, the AGPGA inserts folded faster than the corresponding SPLAT inserts in all cases.

**TABLE 2:** Folding rates and amplitudes.

Insert	Insert Position	Folding Parameters			
		Rate [1/min]	Rate Error	Yield [Fraction Folded]	Yield Error
WT	No insert	0.123	±0.007	0.885	±0.017
SPLAT x 1	Loop 2	0.081	±0.002	0.916	±0.009
SPLAT x 2	Loop 2	0.061	±0.003	0.902	±0.020
SPLAT x 4	Loop 2	0.048	±0.004	0.874	±0.028
AGPGA x 1	Loop 2	0.103	±0.006	0.897	±0.017
AGPGA x 2	Loop 2	0.091	±0.006	0.877	±0.021
AGPGA x 4	Loop 2	0.064	±0.005	0.860	±0.028
SPLAT x 1	Loop 3	0.142	±0.005	0.896	±0.011
SPLAT x 2	Loop 3	0.116	±0.004	0.924	±0.010
SPLAT x 4	Loop 3	0.068	±0.001	0.905	±0.006
AGPGA x 1	Loop 3	0.216	±0.007	0.909	±0.008
AGPGA x 2	Loop 3	0.186	±0.007	0.878	±0.009
AGPGA x 4	Loop 3	0.141	±0.004	0.888	±0.009
SPLAT x 4 L2L3	Both loops	0.039	±0.003	0.790	±0.030
AGPGA x 4 L2L3	Both loops	Did not fold in this assay			

Yield is the fraction of folded protein in relation to total protein in the sample (as determined by densitometry).

### 3.3 *In vivo* Folding and Stress Responses

**FIGURE 3:**
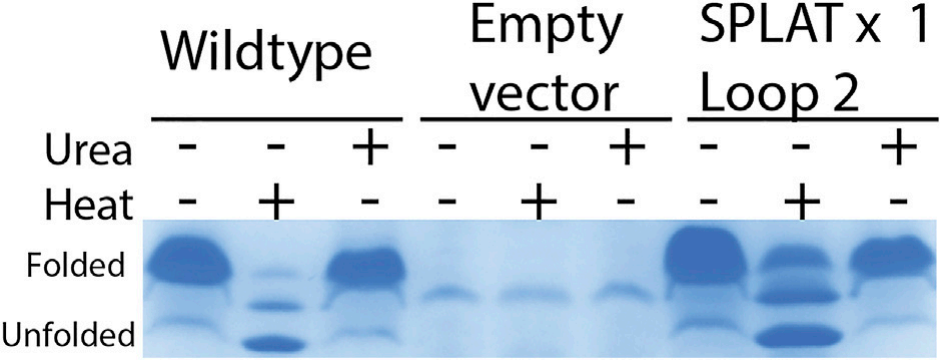
*in vivo* folding of OmpX and variants overexpressed in *E. coli* BL21ΔOmpX. Only the WT OmpX sample, and empty vector control (pET22b) and the sample of the SPLAT x one L2 insert is shown here to exemplify the qualitative nature of this assay. For details of all folding results for all constructs, see Table 3. Gel images demonstrating folding *in vivo* is provided in supplement 2. OM preparations were loaded without heating, heated at 95 °C for 10 min, or washed with urea. OmpX and SPLATx1 L2 folded *in vivo* and resisted the urea wash, suggesting proper membrane insertion.

To assess whether the different insertions would also support proper membrane insertion and folding *in vivo*, we isolated OM fractions after expression of constructs that included the native OmpX signal peptide. As for the *in vitro*-folded samples, all OmpX variants did display heat shifts upon heating of the samples in SDS sample buffer, except for the sample AGPGAx4L2L3 that folds neither *in vitro* nor *in vivo*. By washing membrane fractions in highly concentrated urea buffers, we were also able to assess whether the proteins were correctly inserted or only loosely associated with the outer membrane. In many of the samples, urea washing removed some but not all protein, suggesting that at least a major fraction of the OmpX protein variants were correctly inserted and folded. Figure 3 shows an example gel for this analysis, and Table 3 summarizes the results for all constructs tested. Gel PNGs showcasing the outer membrane fractions are provided in supplement 2.

**TABLE 3:** Qualitative assessment of outer membrane expression and folded state.

Insert	Insert Position	*In vivo* folding^a^			
		Sample (Folded Band)	Sample (Unfolded Band)	Heated Sample Shifts?	Urea Wash
WT	No insert	+++	-	+++	As sample
SPLAT x 1	Loop 2	+++	-	++	As sample
SPLAT x 2	Loop 2	+++	+	+	Less unfolded band compared to sample
SPLAT x 4	Loop 2	++	+	+	Less folded band compared to sample
AGPGA x 1	Loop 2	+++	+	+	Less unfolded band compared to sample
AGPGA x 2	Loop 2	+++	+	+	Less unfolded band compared to sample
AGPGA x 4	Loop 2	+++	+	+	Less folded band compared to sample
SPLAT x 1	Loop 3	++	-	++	As sample
SPLAT x 2	Loop 3	++	+	+	Less unfolded band compared to sample
SPLAT x 4	Loop 3	++	++	+/-	Less folded band compared to sample
AGPGA x 1	Loop 3	+++	+	++	As sample
AGPGA x 2	Loop 3	+++	++	+/-	Less unfolded band compared to sample
AGPGA x 4	Loop 3	++	-	++ (shifts minimally)	As sample
SPLAT x 4 L2L3	Both loops	+	-	+	As sample
AGPGA x 4 L2L3	Both loops	No folded band observed			

a Please note that because of high background, quantification of folding is impossible in these samples. Only the qualitative presence of a band shift upon heating, combined with subjective perception of band intensity, is represented in this table. The original gels are part of the Supplementary Material S1.

### 3.4 Modeling and Simulations of OmpX Constructs

In order to quantify the effects of the loop insertions on OmpX structure and dynamics, we ran two 500-ns MD simulations of the wildtype OmpX as well as 13 mutants with varying degrees of AGPGA and SPLAT insertions in loops 2 and/or 3, all in a *E. coli* K12 outer membrane (see Supplementary Table S3; Figure 1). While we cannot simulate the folding process itself, we can attempt to find features of the folded states that correlate with folding rates. For example, we measured the contact area between loops 2 and 3. The results show that the L2L3SPLAT4X system (double mutant, hereafter) had the highest inter-loop contact area over time, while the wildtype had the lowest, due at least in part to their respective lengths (see Supplementary Figures S1 and S2). We also found that increasing the number of SPLAT repeats in loop 2 increased the contact area between the two loops. However, unexpectedly, increasing the number of SPLAT repeats in loop 3 or AGPGA inserts in either loop did not increase the contact area.

**FIGURE 4:**
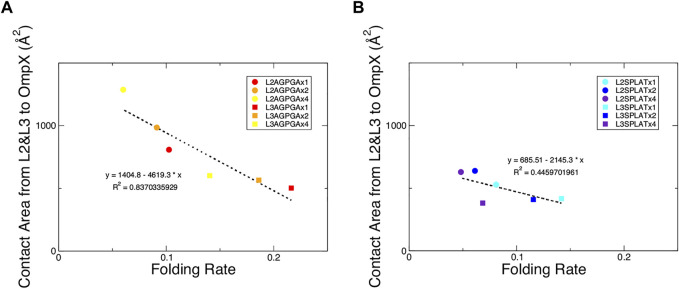
Contact area of loops 2 and 3 with the rest of the protein show an inverse relationship with the folding rate of the constructs. **(A)** Contact area of loops 2 and 3 with the rest of the protein from the AGPGA-insert constructs. The red, orange, and yellow dots represent increasing number of AGPGA inserts (one, two, or four). **(B)** Contact area for the SPLAT-insert constructs. The blue, navy, and purple dots represent the increasing number of SPLAT inserts (one, two, or four). Circular dots represent constructs with inserts in the loop 2, and square dots represent constructs with inserts in loop 3. The linear fit for each is shown as a black dotted line in each panel with the equation of the line and corresponding *R* ^2^ coefficient.

**FIGURE 5:**
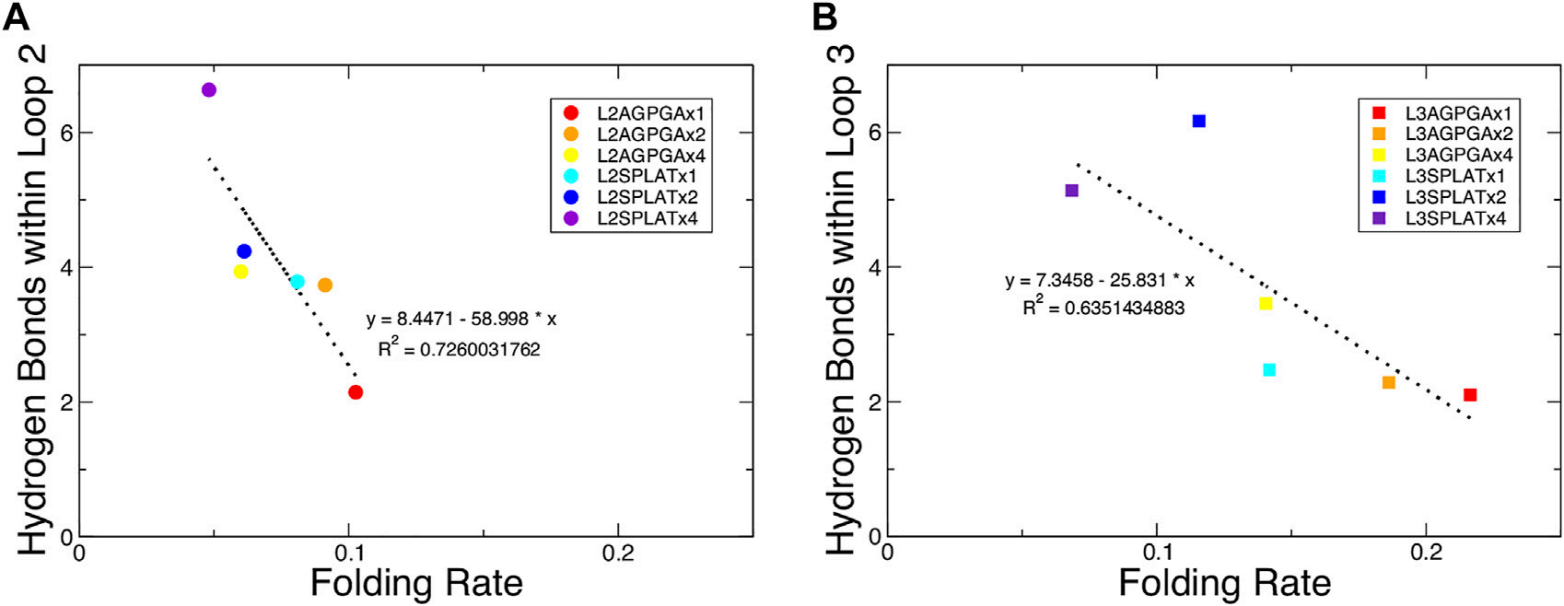
Average number of hydrogen bonds within loop 2 or loop 3 show an inverse relationship with the folding rate of the constructs. **(A,B)** Average number of hydrogen bonds within loop 2 or loop 3 for **(A)** the loop 2 inserts and **(B)** the loop 3 inserts. The red, orange, and yellow dots represent an increasing number of AGPGA inserts (one, two, or four) in the constructs, and the blue, navy, and purple dots represent an increasing number of SPLAT inserts (one, two, or four) in the constructs. Circular dots represent constructs with inserts in loop 2, and square dots represent constructs with inserts in loop 3. The linear fit for each is shown as a black dotted line in each panel with the equation of the line and corresponding *R* ^2^ coefficient.

We also measured the interaction between loops 2 and 3 and the rest of the protein. The results show that while the contact area of constructs with SPLAT inserts in loop 2 increased over time, there was not a clear relationship with the number of SPLAT inserts (see Supplementary Figure S3). The contact area was comparably more stable over time for loop 3 inserts, however (see Supplementary Figures S3, 4). In addition to looking at the contact area over time, we also calculated the average contact area between loops 2 and 3 with the rest of the protein. When plotted against the folding rates measured from the experiment, we find an inverse correlation, i.e., more contact area is associated with a lower folding rate (Figure 4). This suggests that increased interactions between loops 2 and 3 with the rest of the protein negatively impact the folding rate of the protein. In addition, when we calculated the average number of hydrogen bonds within loop 2 or loop 3, we found that the number of hydrogen bonds are inversely correlated with the folding rates (Figure 5). These hydrogen bonds are almost all formed between the backbone atoms of residues outside of the inserts. In contrast, there was no correlation whatsoever between hydrophobic contact area between loops 2 and 3 and the folding rate (Supplementary Figure S7).

We also considered if the inserts destabilized the protein overall. We measured the root-mean-square fluctuations (RMSF) for each of the mutants, finding that while fluctuations in loops 2 and 3 increased noticeably with the size of the insert, the protein overall was largely unaffected (Supplementary Figure S8). We noticed that there is significant contact area between loops 2 and 3 and the outer membrane, which was primarily contributed by interactions with the glycans of the LPS molecules in the outer leaflet. We note that this contact area wasn’t correlated with the size of the inserts nor the folding rate (Supplementary Figure S9).

## 4 Discussion

**FIGURE 6:**
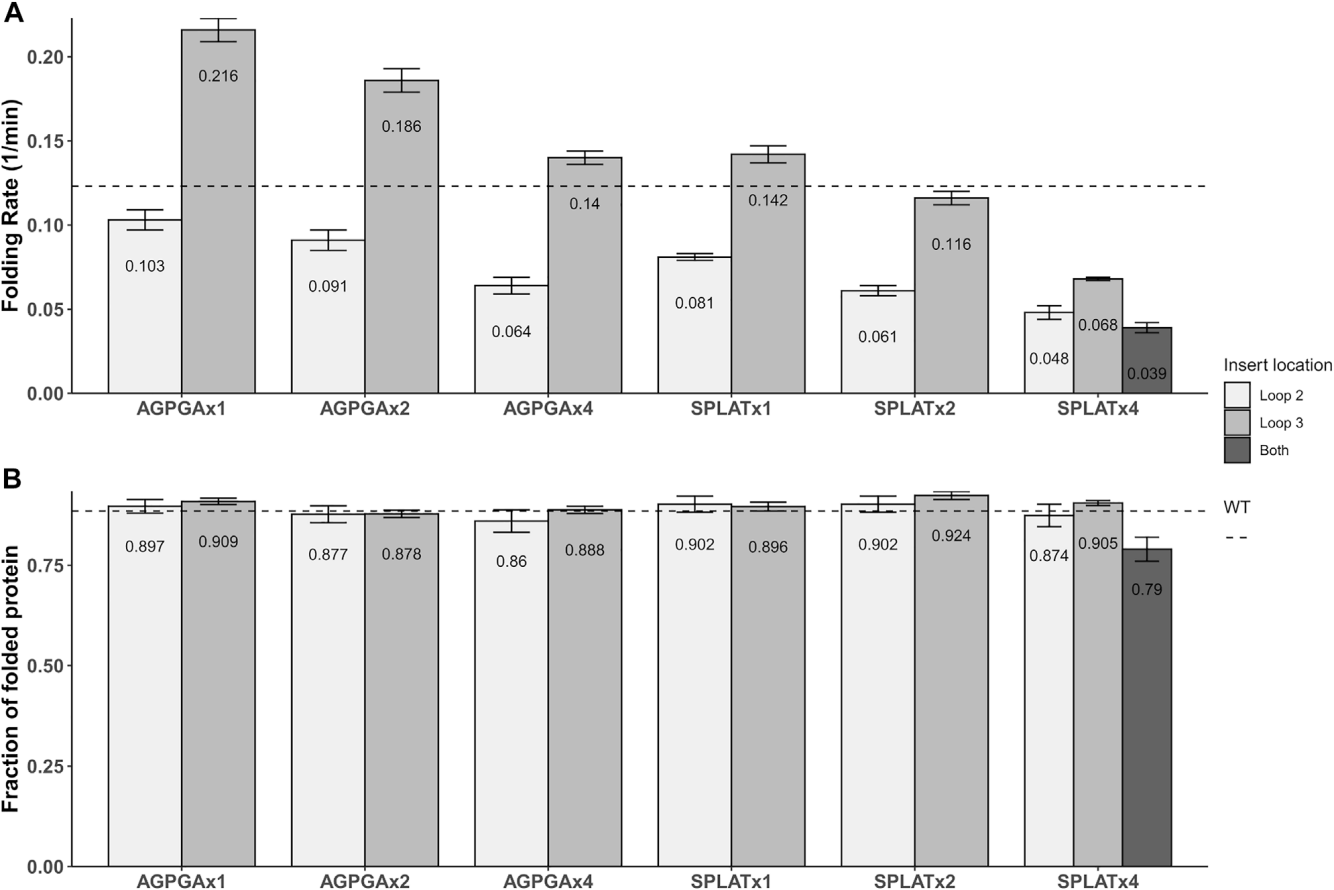
Graphical representation of the folding rates **(A)** and folding yields **(B)** from Table 2. The dotted line denotes the values for wildtype OmpX. Note that while folding rate generally decreases with the number of insert repeats, some inserts actually increase the folding rate (but not the yield).

The aim of this work was to achieve a better overview of what types of loop insertions are tolerated in outer membrane proteins (OMPs)–both for *in vitro* and *in vivo* folding. As OMPs are useful for surface display of antigens and other functional protein units (Lång, 2000), this question is relevant for a variety of biotechnology applications. As a starting point, we decided to compare two different, short loop inserts and repeated these units up to 4x, inserting them into different loops of our model protein OmpX. The key difference between the two inserts is their hydrophobicity, and we assumed that this would directly affect both membrane insertion *in vivo* and folding *in vitro*. What we found is that increasing the repeat number of both inserts in both loop positions slowed down folding systematically, and that the more hydrophilic AGPGA inserts folded faster than their equivalent, more hydrophobic SPLAT inserts. What we found surprising is that the construct AGPGAx1L3, x2L3, x4L3 and also SPLATx1L3 actually folded significantly faster than the wildtype protein. This is visualized in Figure 6 based on the results from Table 2. This might indicate that loop 3 has some constraints related to folding, and that inserting a rather flexible loop in this position loosens these constraints significantly. It is conceivable that loop 3 is directly involved in some yet unknown function of OmpX *in vivo* that would demand a specific structure that does not fold optimally. It has been speculated previously that OmpX is an adhesin, and that the relatively long external loops might be the adhesion interface in this case (Vogt and Schulz, 1999).

Maybe similarly unexpected, only one construct did not fold at all under our assay conditions: a construct where AGPGA was inserted 4x in both loops simultaneously–while a similar construct with the more hydrophobic SPLAT insert 4x in both loops did fold slowly but efficiently *in vitro*, to almost wildtype yields, and did insert into the outer membrane *in vivo*. This shows that while OmpX is a very robust system that tolerates insertions of different size in different (and even multiple) loops, there are limitations.

To better understand the effects of our loop insertions *in vivo*, we attempted to perform reporter gene assays developed specifically to detect different types of bacterial envelope stresses (Steenhuis et al., 2021). The reporter systems (a kind gift by Joen Luirink) have previously been used successfully as detectors of σE, heat-shock and Rcs (regulation of capsular polysaccharide synthesis) stress. Since these stress responses are linked to membrane integrity or periplasmic protein accumulation, we reasoned that our OmpX-variants would be likely candidates to activate them. Likewise, the constructs would have been a useful tool to benchmark the *in vivo* folding-related stress from our constructs. Unfortunately, these assays were inconclusive. The high background that we observed is probably due to the relatively leaky expression of the pET expression system. But we cannot exclude that our constructs in fact do not stress the cells significantly. In favor of this hypothesis, we see that all constructs were able to insert into the outer membrane at least to a large extent after autoinduction (Table 3). Only for some of the constructs, it seemed that there is some additional accumulation of unfolded or misfolded protein–typically the ones with a high number of repeats in the inserts.

Finally, we performed simulations of mature OmpX in an outer membrane with the different inserts. This revealed trends that we were unable to access with our *in vitro* and *in vivo* experiments. When we plotted the contact area between loop 2 and loop 3, we found an inverse correlation, i.e., more contact area is associated with a lower folding rate (Figure 4). This suggests that increased interactions between loops 2 and 3 with the rest of the protein negatively impact the folding rate of the protein. This might also give a more detailed explanation of what is happening to the L3 inserts that fold faster *in vitro*: possibly in this case, some inter-loop interactions are partly disrupted by the very short single-repeat inserts in this position, compared to OmpX wildtype.

A previous study that tested different OmpA mutants with different lengths of extracellular loops found a connection between the degree of hydrogen bonding within the loops and the folding rate (Franklin et al., 2021). Following this, we measured the number of hydrogen bonds over time within loop 2 or loop 3 for each of the simulated constructs. The results show that, while the double mutant had the highest number of hydrogen bonds for one replica, there was again not a clear observable trend between the number of hydrogen bonds and the number of inserts in either loop 2 or loop 3 (see Supplementary Figures S5, 6). However, when we calculated the average number of hydrogen bonds within loop 2 or loop 3, we found that the number of hydrogen bonds are inversely correlated with the folding rates (Figure 5). This finding indicates that both the contacts made by loop 2 or loop 3 with the rest of the protein, along with the intra-loop interactions, both contribute to slowing down the overall rate of protein folding and insertion. Published models for the mechanism of OmpX folding (Raschle et al., 2016; Rath et al., 2019) suggest a two-step folding process, where the rate-limiting step is the formation of an intermediate with an extensive hydrogen-bonding network that brings the N- and C-terminal strands together; from there, OmpX folds further into the final, robust β-barrel structure. In this model, inserting longer and longer loops would lead to longer distance between the terminal loops, and thus to a decreased rate of folding. Our results clearly demonstrate that this is only one of the factors that impact OmpX folding, as we find relatively long insertions that still fold faster than the wildtype protein–but the model does explain the inverse correlation of folding rate with the length of the insert within a set of equivalent loop insertions.

In summary, while of course different additional inserts might give a more complete picture of what are favorable inserts into OMPs for optimal membrane insertion and folding, we conclude that inter-loop contacts and hydrogen bonding are key features that inversely correlate with folding rates, and that more hydrophobic inserts seem to fold slower compared to more hydrophilic counterparts.

## Data Availability

The original contributions presented in the study are included in the article/Supplementary Material. Further inquiries can be directed to the corresponding author.
