# Supplementary material for: The Role of Extracellular Loops in the Folding of Outer Membrane Protein X (OmpX) of Escherichia coli
Source: Front Mol Biosci. 2022 Jul 14;9:918480. doi: 10.3389/fmolb.2022.918480 (PMC9329534; doi:10.3389/fmolb.2022.918480)
Supplement: Supplementary file 1 [file DataSheet1.PDF]

Table S1. Primers used for preparation of the constructs used in this study.
(as described in Table 2 in the main text):

Primers	Sequence 5'-3'
Primer 1 SPLATfwdL2	AGCCCACTAGCAACAGGTGACTACAACAAAAACCAGTACTACG
Primer 2 AGPGAfwdL2	GCAGGACCAGGAGCAGGTGACTACAACAAAAACCAGTACTACG
Primer 3 revL2	AGAGCTTGCAGTACGGCTT
Primer 4 SPLATfwdL3	AGCCCACTAGCAACAACCTACAAACACGACACCAGC
Primer 5 AGPGAfwdL3	GCAGGACCAGGAGCAACCTACAAACACGACACCAGC
Primer 6 revL3	CGGGTATTCAGTGGTCTGGAATTT
Primer 7 AGPGAx2revL2	TGCTCCTGGTCCTGCGCTTGCAGTACGGCTTTTCTCG
Primer 8 SPLATx2revL2	TGTTGCTAGTGGGctAGAGCTTGCAGTACGGCTT
Primer 9 SPLATx2revL3	TGTTGCTAGTGGAGACGGGTATTCAGTGGTCTGGAATTT
Primer 10 AGPGAx2fwdL2	GCGGGTCCGGGTGCGTCTGGTGACTACAACAAAAACCAGTACTAC
Primer 11 SPLATx2fwdL2	AGTCCGCTTGCGACAGGTGACTACAACAAAAACCAGTACTACG
Primer 12 AGPGAx2fwdL3	GCGGGTCCGGGTGCGTACAAACACGACACCAGCGACTACG
Primer 13 AGPGAx2revL3	TGCTCCTGGTCCTGCGGTCGGGTATTCAGTGGTCTGGAATT
Primer 14 SPLATx4fwdL2	AGTCCGCTTGCGACAAGTCCGCTTGCGACAGGTGACTACAACAAAAACCAGTACTACGGC
Primer 15 SPLATx4revL2	TGTTGCTAGTGGGCTTGTTGCTAGTGGGCTAGAGCTTGCAGTACGGCTTTTCTCG
Primer 16 SPLATx4fwdL3	AGCCCACTTGCAACAAGCCCACTAGCAACTCCGACCTACAAACACGACACCAGC
Primer 17 SPLATx4revL3	TGTAGCTAGAGGAGAAGTAGCAAGAGGTGAGTATTCAGTGGTCTGGAATTTACCATAACCCACA
Primer 18 AGPGAx4fwdL2	GCGGGTCCGGGTGCGGCGGGTCCGGGTGCGTCTGGTGACTACAACAAAAACCAGTACTAC
Primer 19 AGPGAx4revL2	TGCTCCTGGTCCTGCTGCTCCTGGTCCTGCGCTTGCAGTACGGCTTTTCTCG
Primer 20 AGPGAx4fwdL3	GCGGGTCCGGGTGCGGCGGGTCCGGGTGCGTACAAACACGACACCAGCGACTACG
Primer 21 AGPGAx4revL3	TGCTCCTGGTCCTGCTGCTCCTGGTCCTGCGGTCGGGTATTCAGTGGTCTGGAATT

Table S2. Plasmids used in this study

Plasmids	Description	Source or reference
WT	pET3b plasmid with OmpX wild-type under control of T7 promoter	Thomas Arnold
SPLAT x 1 L2	pET3b plasmid with 1x SPLAT sequence inserted into Loop 2 of WT OmpX	This study
SPLAT x 2 L2	pET3b plasmid with 2x SPLAT sequence inserted into Loop 2 of WT OmpX	This study
SPLAT x 4 L2	pET3b plasmid with 4x SPLAT sequence inserted into Loop 2 of WT OmpX	This study
AGPGA x 1 L2	pET3b plasmid with 1x AGPGA sequence inserted into Loop 2 of WT OmpX	This study
AGPGA x 2 L2	pET3b plasmid with 2x AGPGA sequence inserted into Loop 2 of WT OmpX	This study
AGPGA x 4 L2	pET3b plasmid with 4x AGPGA sequence inserted into Loop 2 of WT OmpX	This study
SPLAT x 1 L3	pET3b plasmid with 1x SPLAT sequence inserted into Loop 3 of WT OmpX	This study
SPLAT x 2 L3	pET3b plasmid with 2x SPLAT sequence inserted into Loop 3 of WT OmpX	This study
SPLAT x 4 L3	pET3b plasmid with 4x SPLAT sequence inserted into Loop 3 of WT OmpX	This study
AGPGA x 1 L3	pET3b plasmid with 1x AGPGA sequence inserted into Loop 3 of WT OmpX	This study
AGPGA x 2 L3	pET3b plasmid with 2x AGPGA sequence inserted into Loop 3 of WT OmpX	This study
AGPGA x 4 L3	pET3b plasmid with 4x AGPGA sequence inserted into Loop 3 of WT OmpX	This study
SPLAT x 4 L2L3	pET3b plasmid with 4x SPLAT sequence inserted into Loop 2 and 3 of WT OmpX	This study
AGPGA x 4 L2L3	pET3b plasmid with 4x AGPGA sequence inserted into Loop 2 and 3 of WT OmpX	This study

Table S3: OmpX systems simulated

Name	Insert sequence	Copies	Location(s)
Wildtype	N/A	N/A	N/A
L2AGPGA1X	AGPGA	1	S54 and G55
L2AGPGA2X	AGPGA	2	S54 and G55
L2AGPGA4X	AGPGA	4	S54 and G55
L2SPLAT1X	SPLAT	1	S54 and G55
L2SPLAT2X	SPLAT	2	S54 and G55
L2SPLAT4X	SPLAT	4	S54 and G55
L3AGPGA1X	AGPGA	1	P96 and T97
L3AGPGA2X	AGPGA	2	T97 and Y98
L3AGPGA4X	AGPGA	4	T97 and Y98
L3SPLAT1X	SPLAT	1	P96 and T97
L3SPLAT2X	SPLAT	2	P96 and T97
L3SPLAT4X	SPLAT	4	Y95 and P96
L2L3SPLAT4X	SPLAT	4 each	S54 and G55<br>Y95 and P96

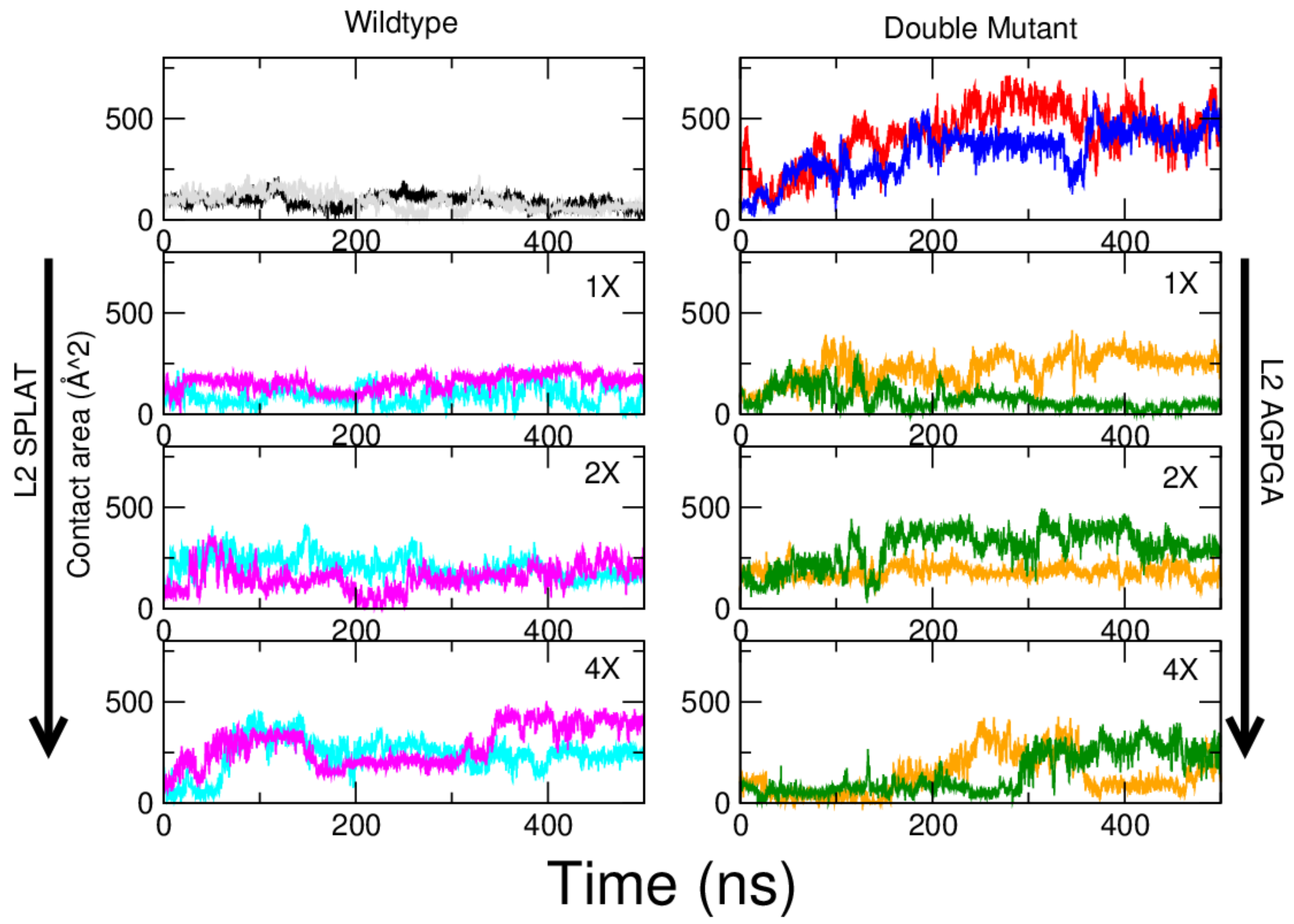

Figure S1: Contact area of loops 2 and 3 for L2 constructs. Top left shows wildtype results with two replicas represented in black and gray. Top right shows double mutant results with two replicas shown in blue and red. The left column shows wildtype and L2 SPLAT constructs with increasing number of insertions going down. The right column shows double mutant and L2 AGPGA constructs with increasing number of insertions going down.

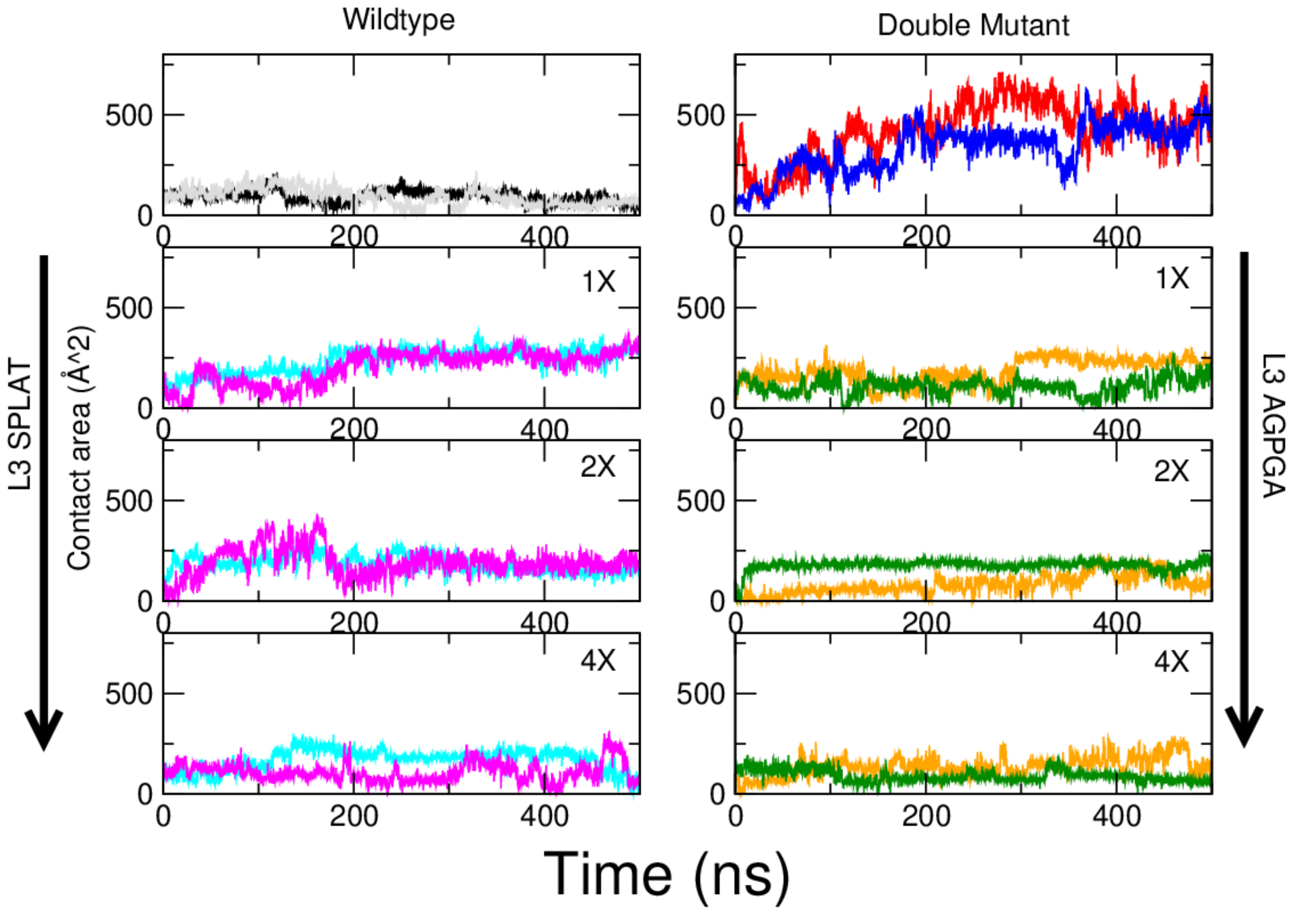

Figure S2: Contact area of loops 2 and 3 for L2 constructs. Top left shows wildtype results with two replicas represented in black and gray. Top right shows double mutant results with two replicas shown in blue and red. The left column shows wildtype and L3 SPLAT constructs with increasing number of insertions going down. The right column shows double mutant and L3 AGPGA constructs with increasing number of insertions going down.

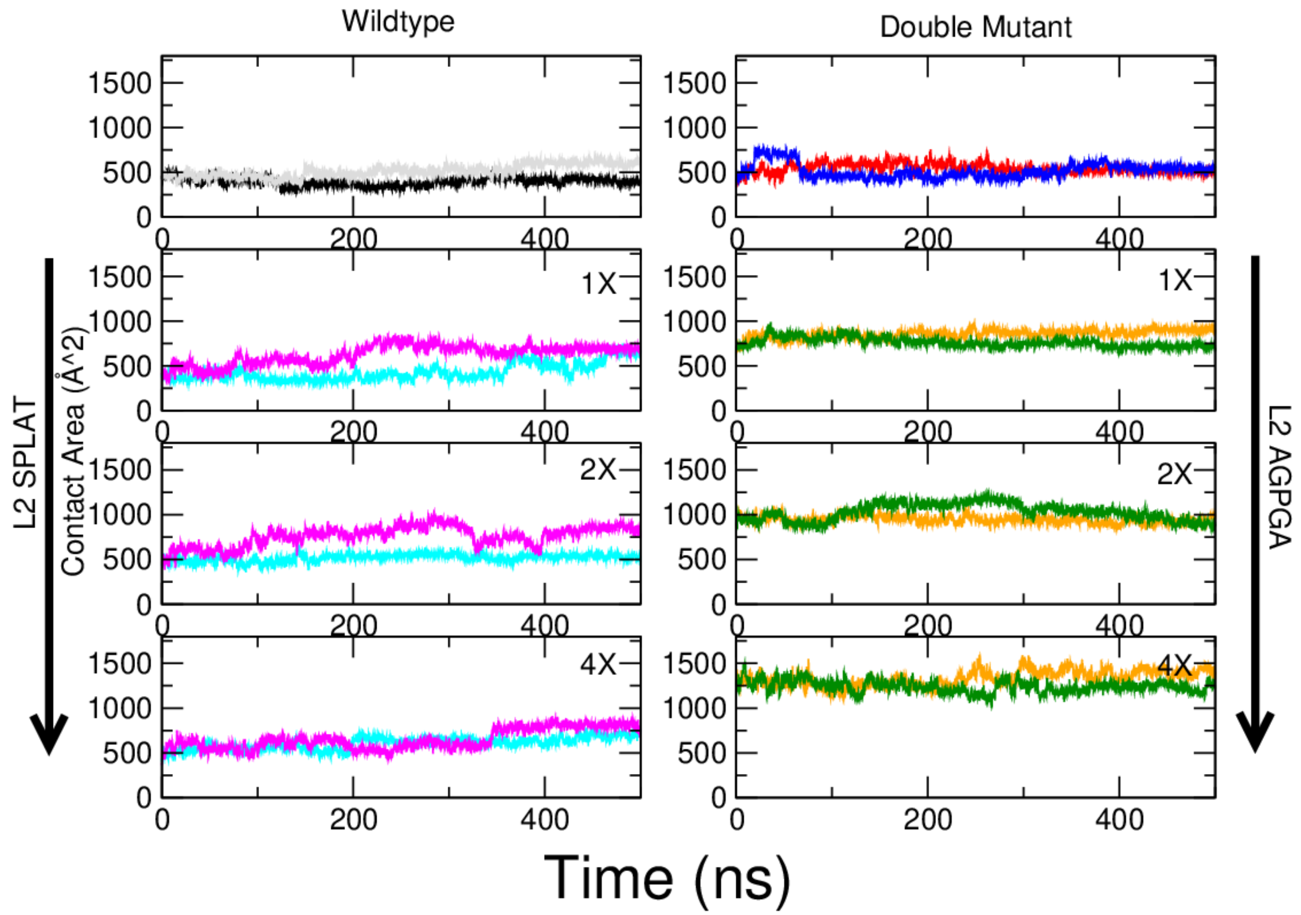

Figure S3: Contact area of loops 2 and 3 with the rest of the protein for L2 constructs. Top left shows wildtype results with two replicas represented in black and gray. Top right shows double mutant results with two replicas shown in blue and red. The left column shows wildtype and L2 SPLAT constructs with increasing number of insertions going down. The right column shows double mutant and L2 AGPGA constructs with increasing number of insertions going down.

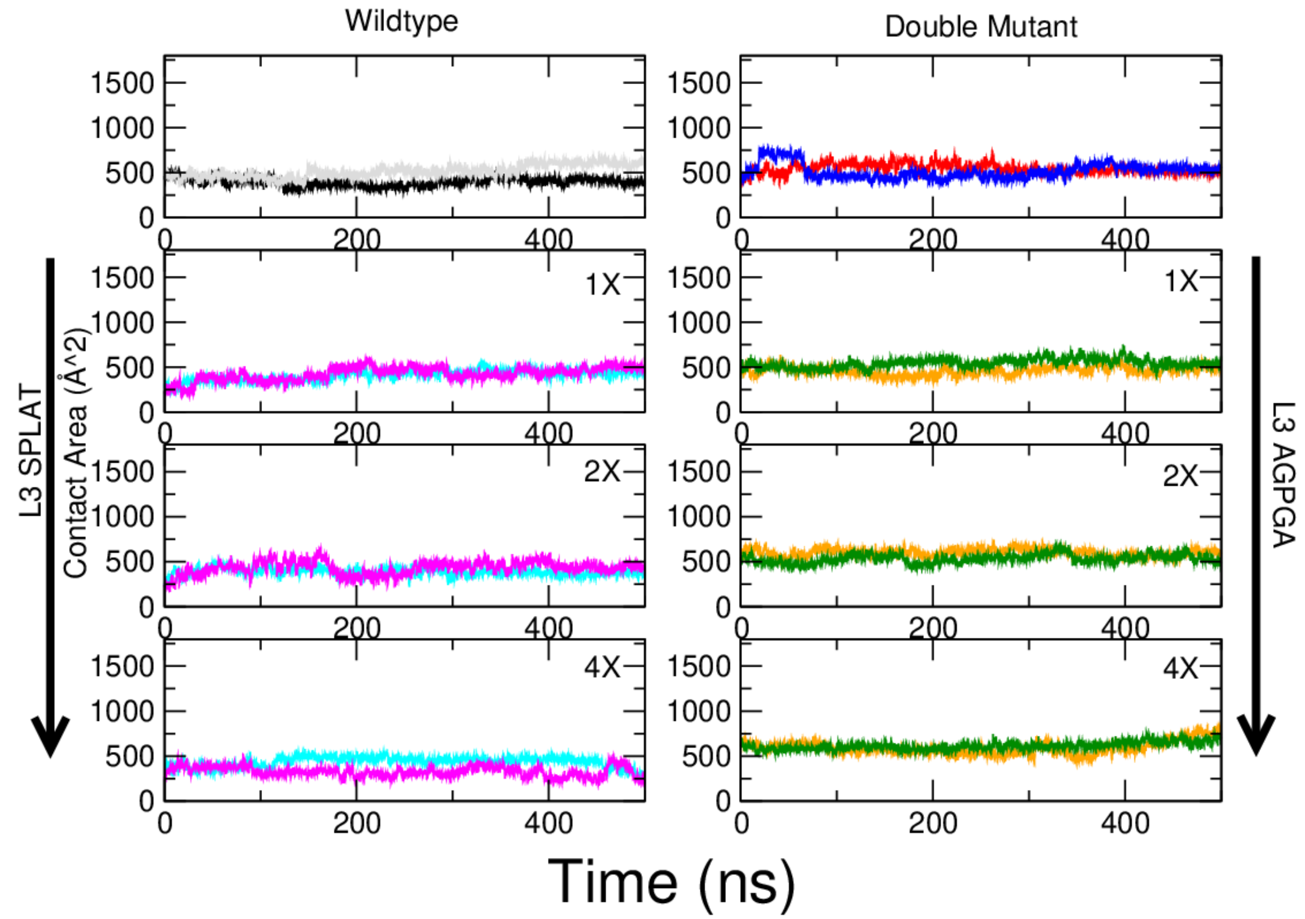

Figure S4: Contact area of loops 2 and 3 with the rest of the protein for L3 constructs. Top left shows wildtype results with two replicas represented in black and gray. Top right shows double mutant results with two replicas shown in blue and red. The left column shows wildtype and L3 SPLAT constructs with increasing number of insertions going down. The right column shows double mutant and L3 AGPGA constructs with increasing number of insertions going down

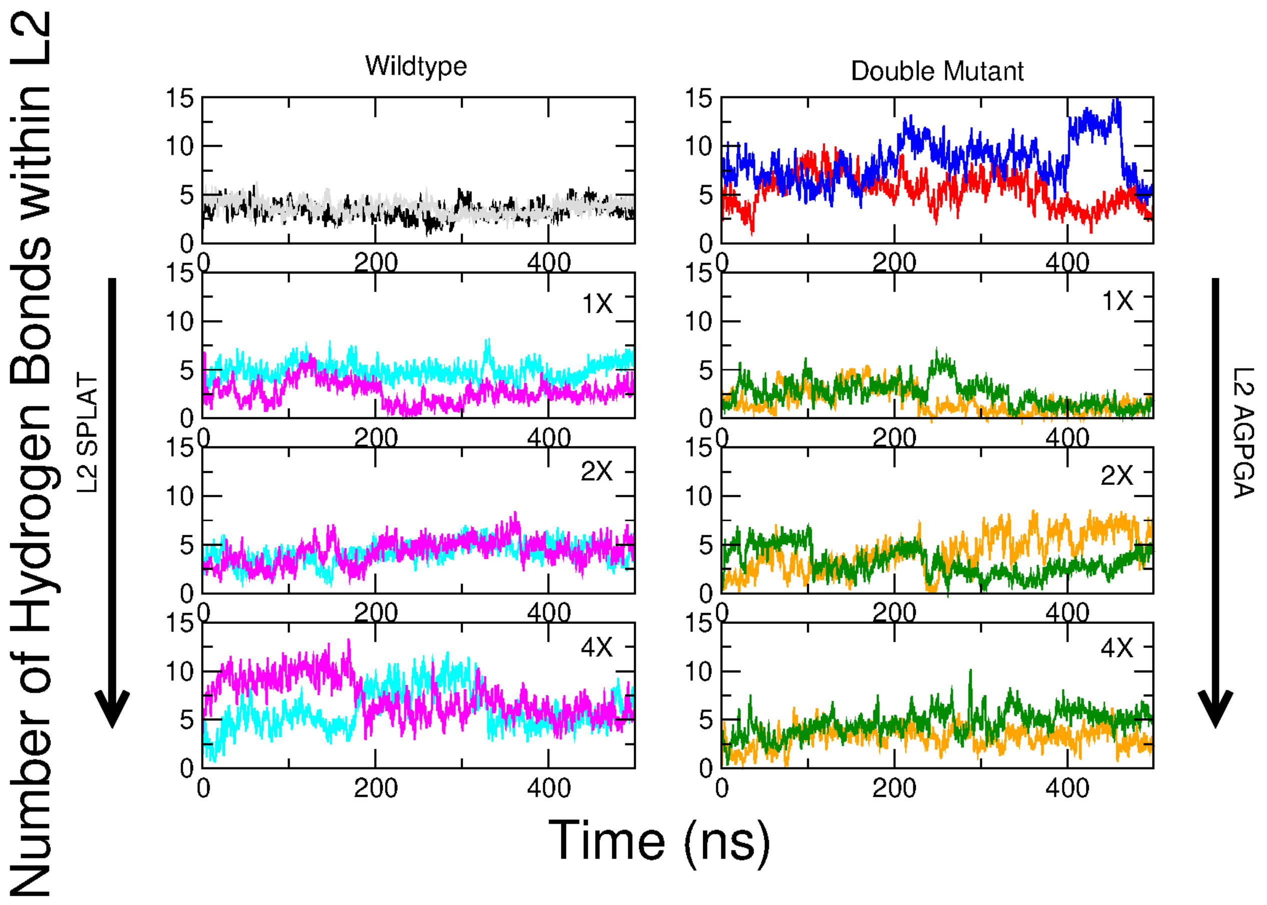

Figure S5: The number of hydrogen bonds within loop 2 or loop 3 for L2 constructs. Top left shows wildtype results with two replicas represented in black and gray. Top right shows double mutant results with two replicas shown in blue and red. The left column shows wildtype and L2 SPLAT constructs with increasing number of insertions going down. The right column shows double mutant and L2 AGPGAconstructs with increasing number of insertions going down.

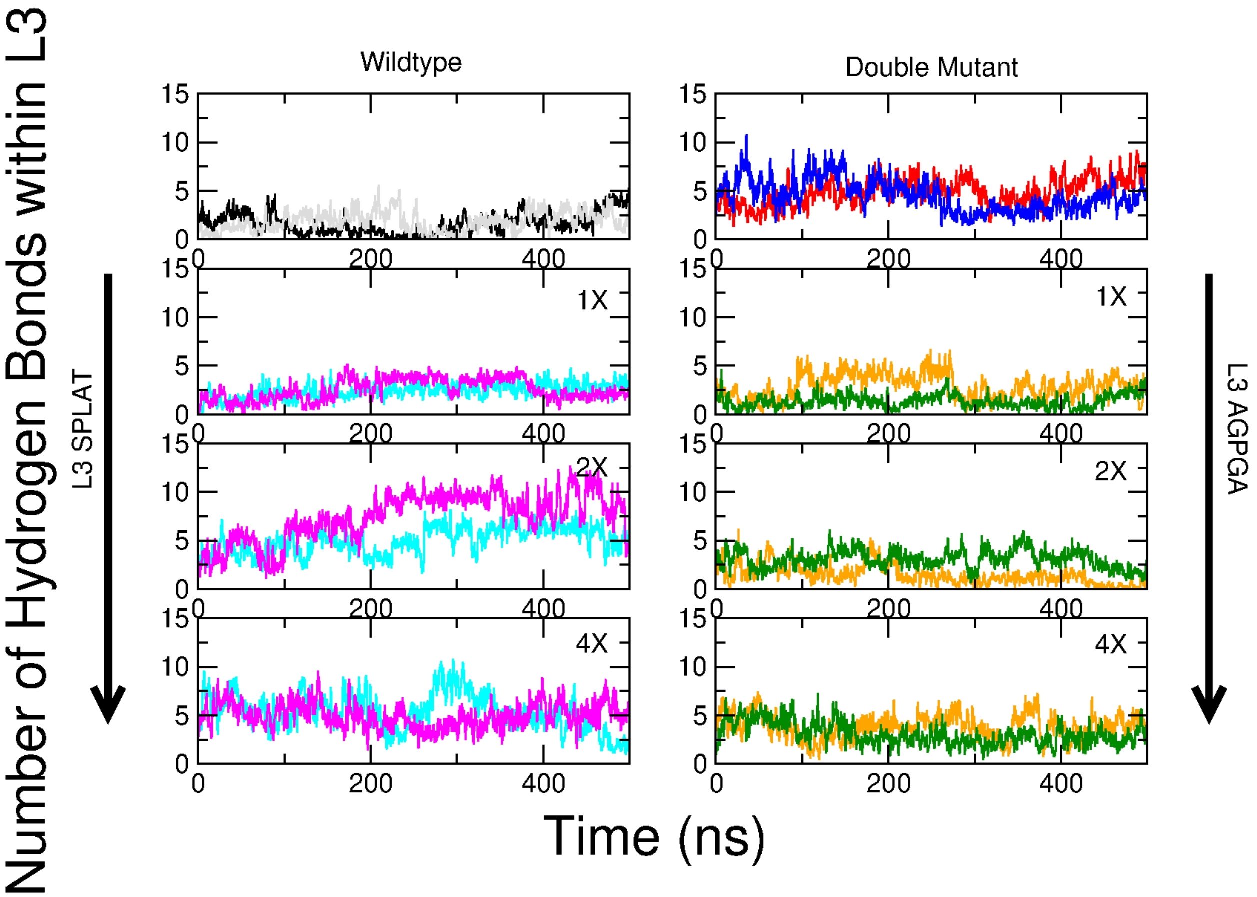

Figure S6: The number of hydrogen bonds within loop 2 or loop 3 for L3 constructs. Top left shows wildtype results with two replicas represented in black and gray. Top right shows double mutant results with two replicas shown in blue and red. The left column shows wildtype and L3 SPLAT constructs with increasing number of insertions going down. The right column shows double mutant and L3 AGPGA constructs with increasing number of insertions going down.

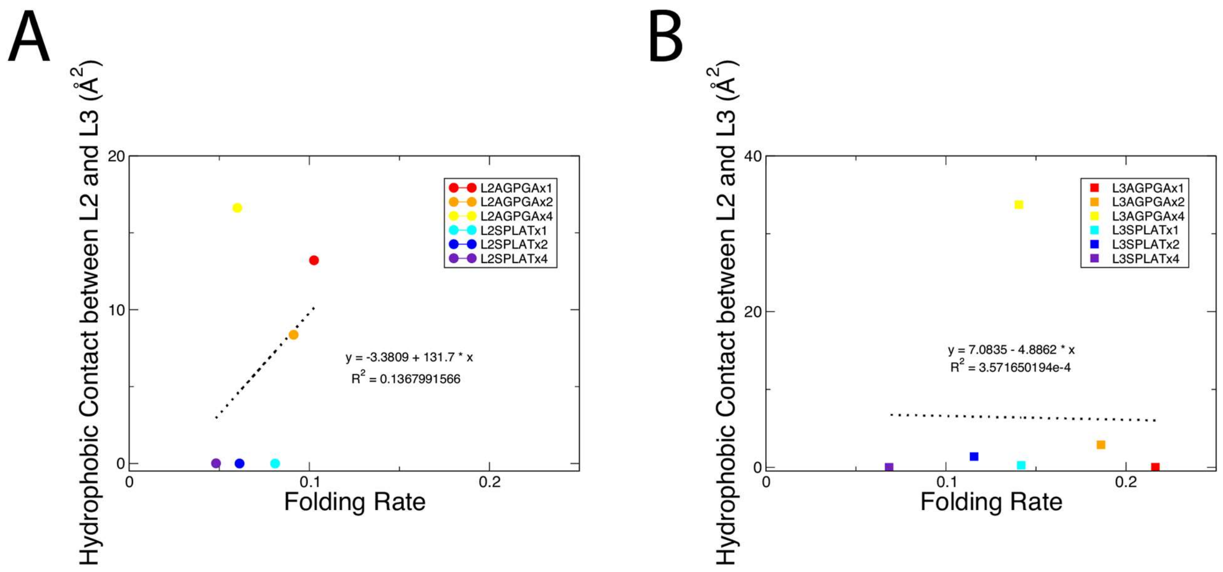

Figure S7: Hydrophobic contact area between loops 2 (A) and 3 (B) vs. the folding rate. Circular dots represent constructs with inserts in the loop 2, and square dots represent constructs with inserts in loop 3. The red, orange, and yellow dots represent increasing number of AGPGA inserts (one, two, or four). The blue, navy, and purple dots represent the increasing number of SPLAT inserts (one, two, or four). The linear fit for each is shown as a black dotted line in each panel with the equation of the line and corresponding R^2 coefficient.

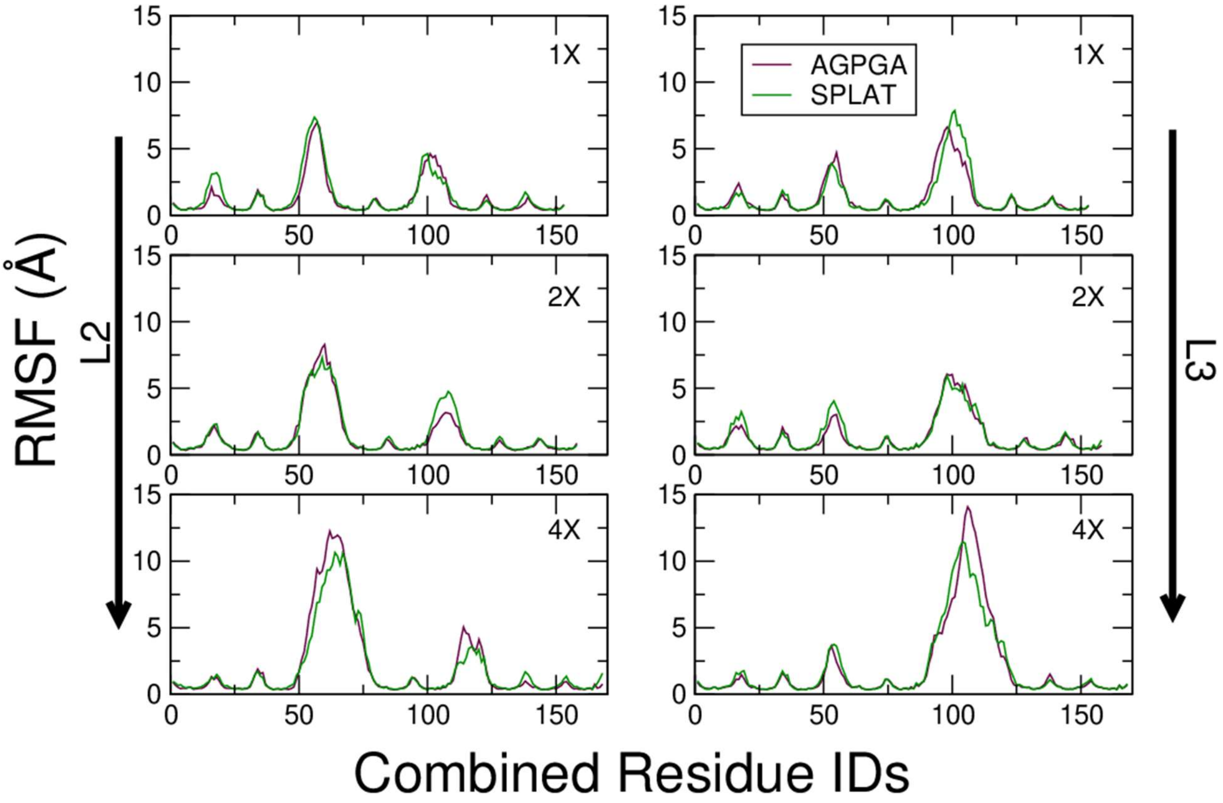

Figure S8: Root-mean-square fluctuations (RMSF) for the each mutant construct. Notably, the RMSF for L2 or L3 goes up with the number of insertions. The neighboring loop, L3 or L2, goes up as well in some cases but not all. The rest of the protein is mostly unaffected.

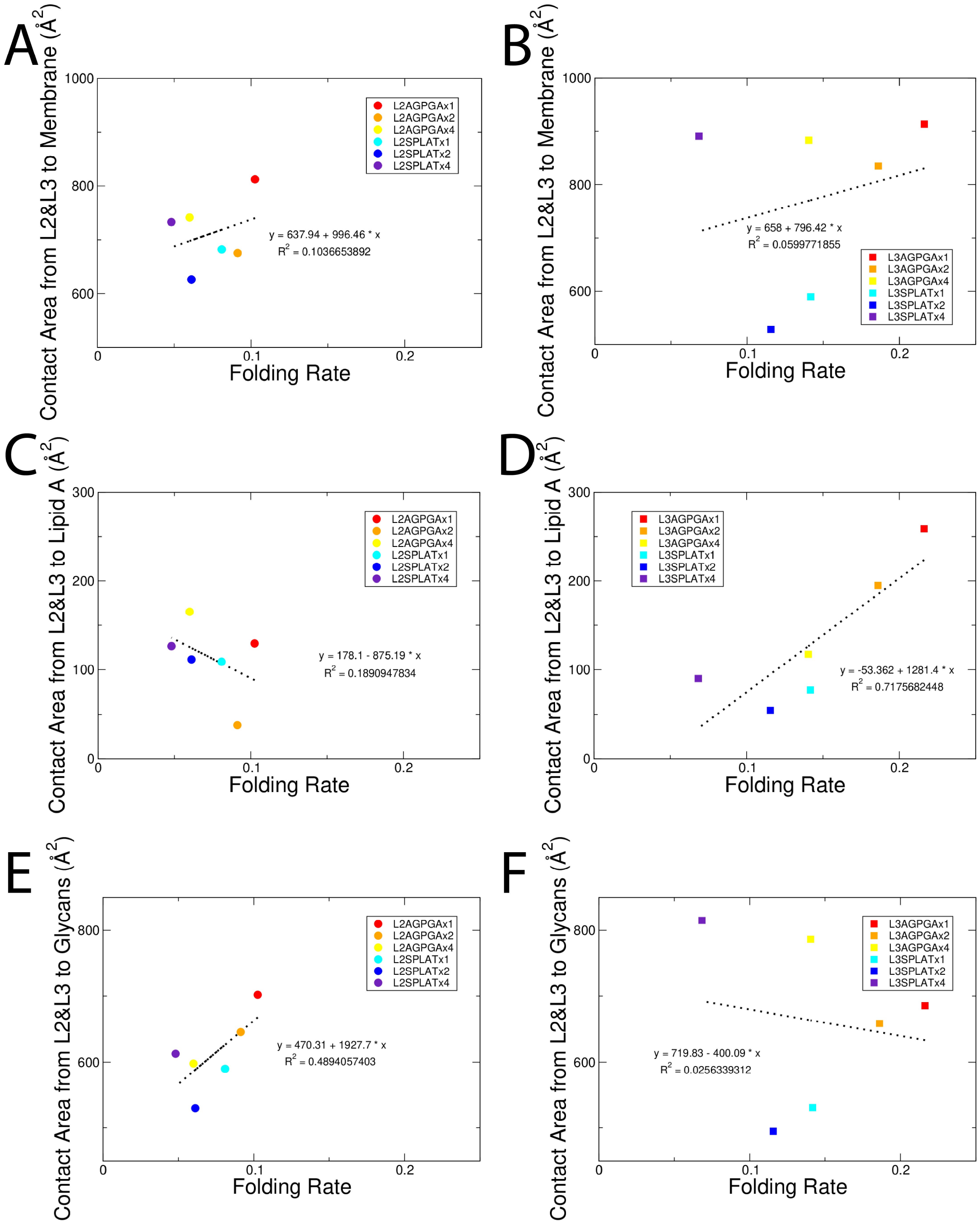

Figure S9: Contact area of loops 2 (A, C, E) and 3 (B, D, F) with the membrane overall (A, B), Lipid A (C, D), and glycans of the LPS (E, F) vs. the folding rate. Circular dots represent constructs with inserts in the loop 2, and square dots represent constructs with inserts in loop 3. The red, orange, and yellow dots represent increasing number of AGPGA inserts (one, two, or four). The blue, navy, and purple dots represent the increasing number of SPLAT inserts (one, two, or four). The linear fit for each is shown as a black dotted line in each panel with the equation of the line and corresponding R^2 coefficient.
