# Supplementary figures and images for: The Role of Extracellular Loops in the Folding of Outer Membrane Protein X (OmpX) of Escherichia coli
Source: Front Mol Biosci. 2022 Jul 14;9:918480. doi: 10.3389/fmolb.2022.918480 (PMC9329534; doi:10.3389/fmolb.2022.918480)

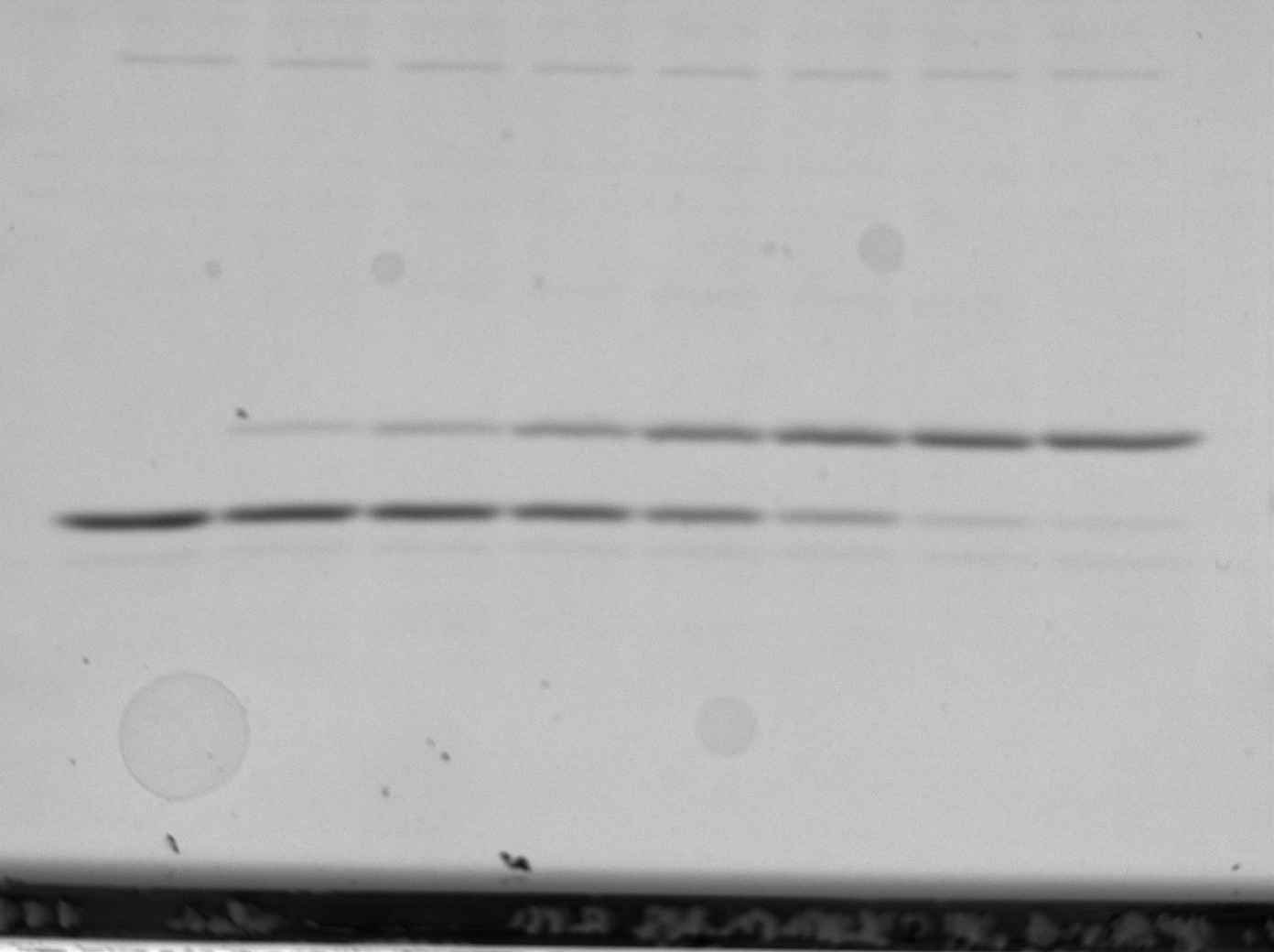

Supplement: Supplementary file 2 [file DataSheet2.zip › Supplement_2a_AGPGA/AGPGA X1 Loop 2/Ax1_L2_1.tif]

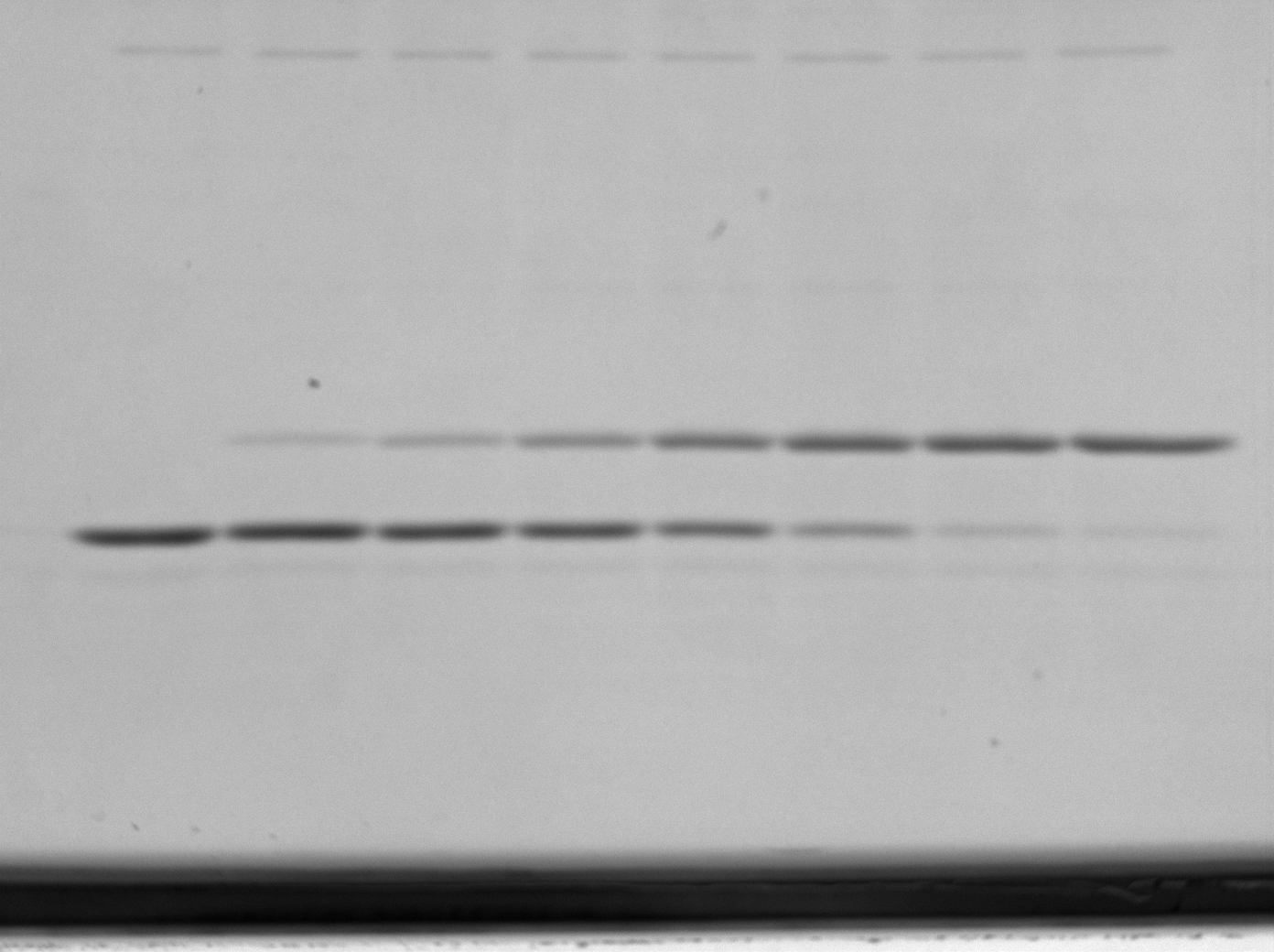

Supplement: Supplementary file 2 [file DataSheet2.zip › Supplement_2a_AGPGA/AGPGA X1 Loop 2/Ax1_L2_2.tif]

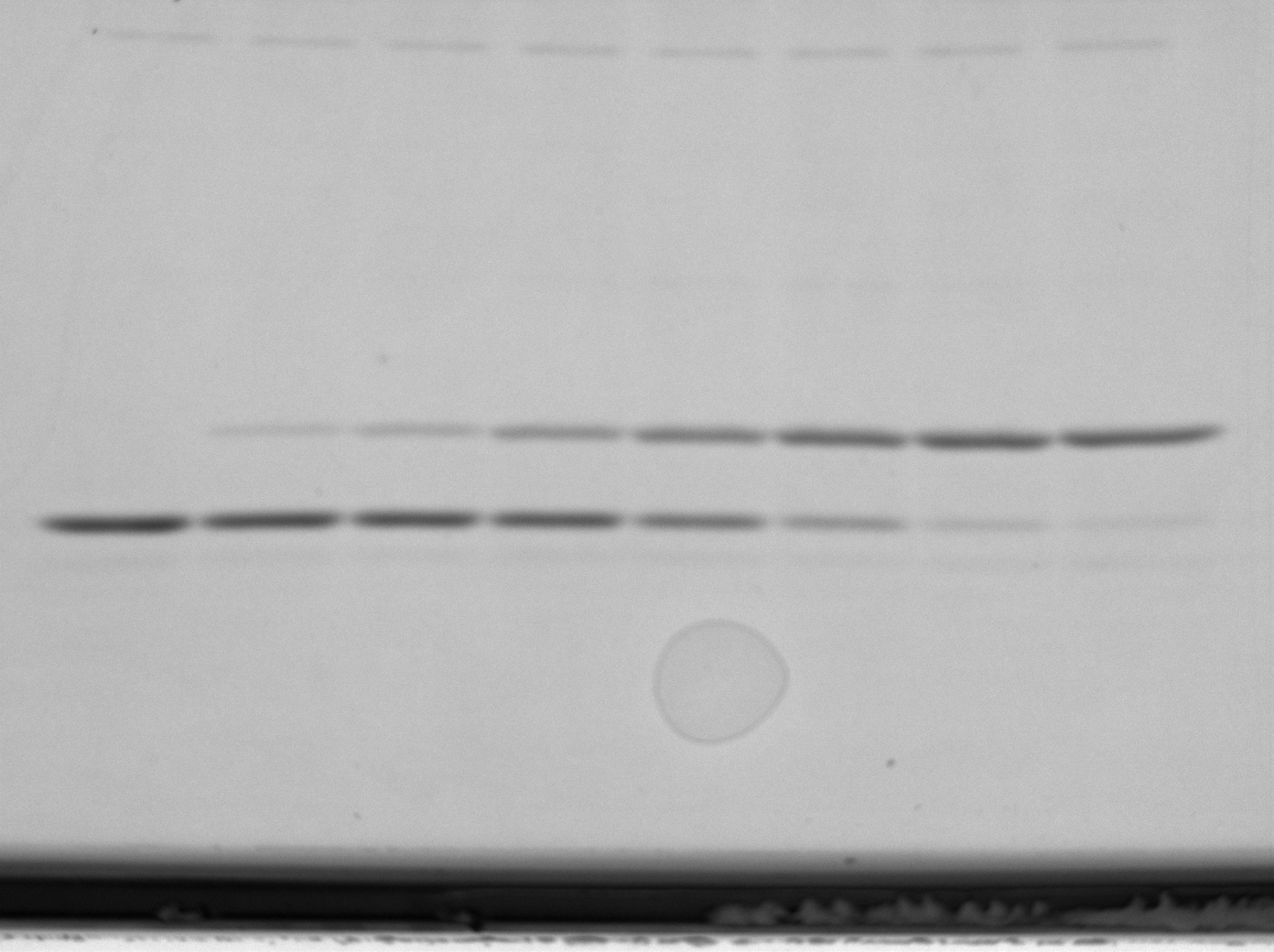

Supplement: Supplementary file 2 [file DataSheet2.zip › Supplement_2a_AGPGA/AGPGA X1 Loop 2/Ax1_L2_3.tif]

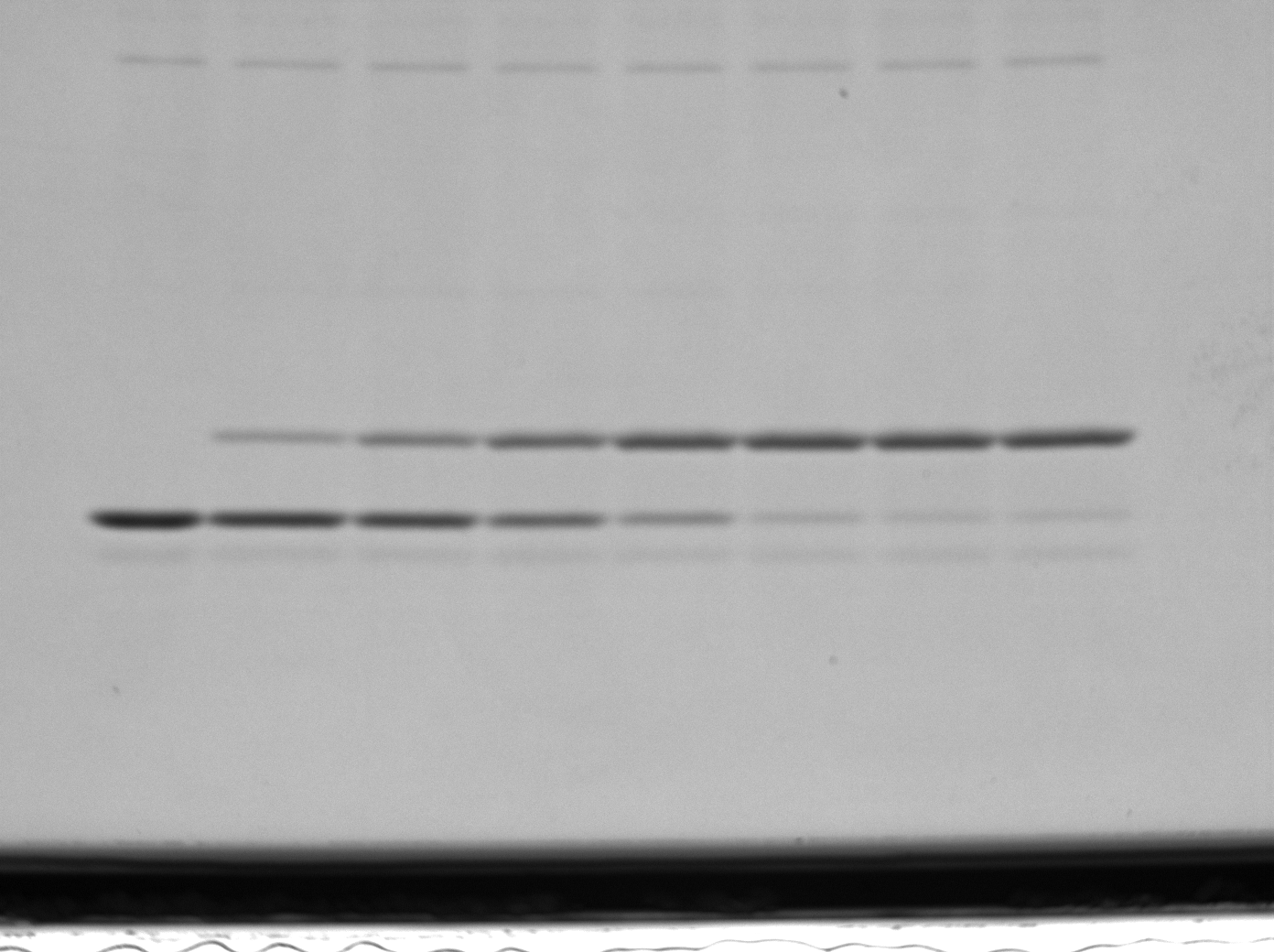

Supplement: Supplementary file 2 [file DataSheet2.zip › Supplement_2a_AGPGA/AGPGA X1 Loop 3/Ax1_L3_1.tif]

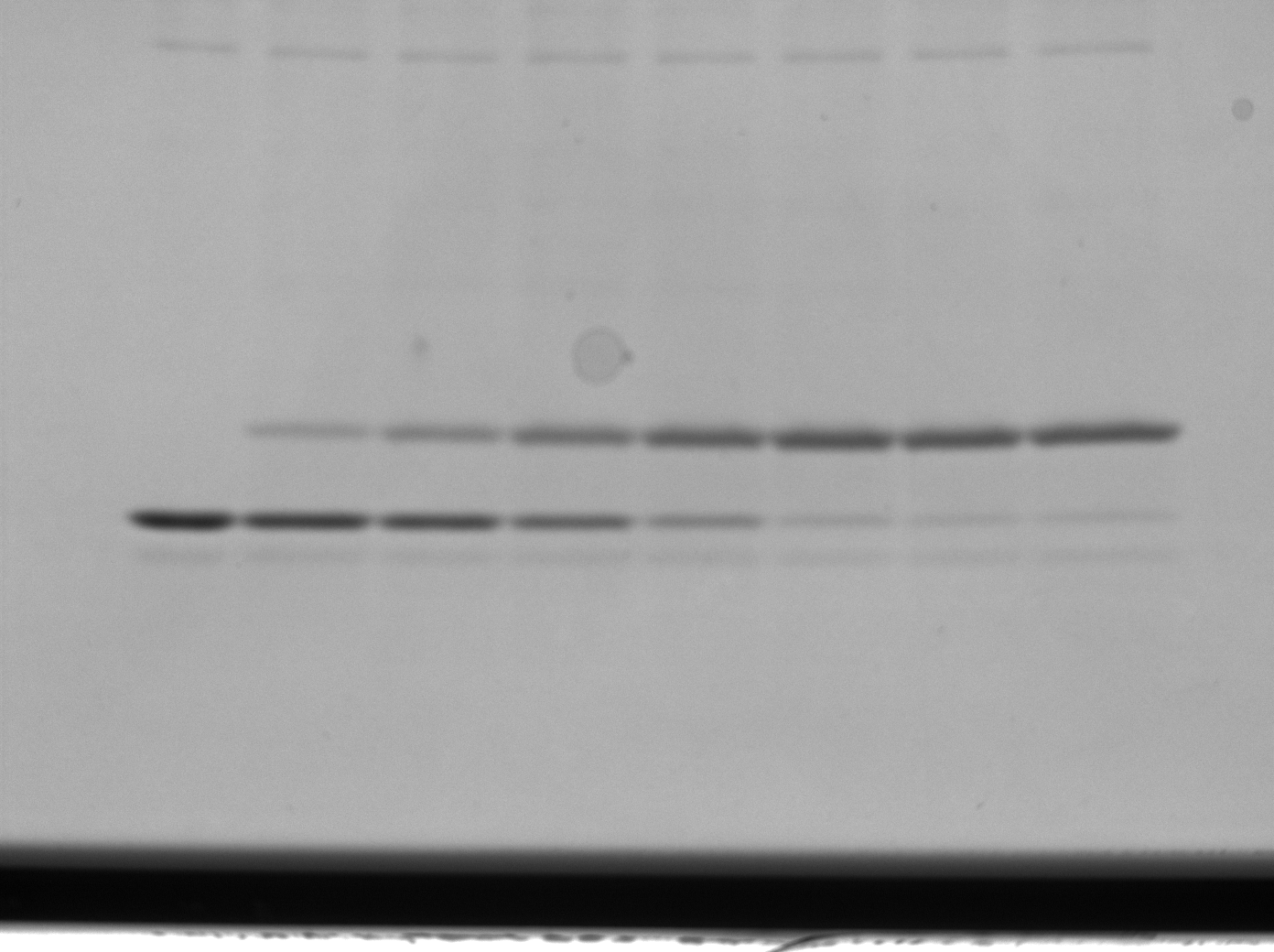

Supplement: Supplementary file 2 [file DataSheet2.zip › Supplement_2a_AGPGA/AGPGA X1 Loop 3/Ax1_L3_2.tif]

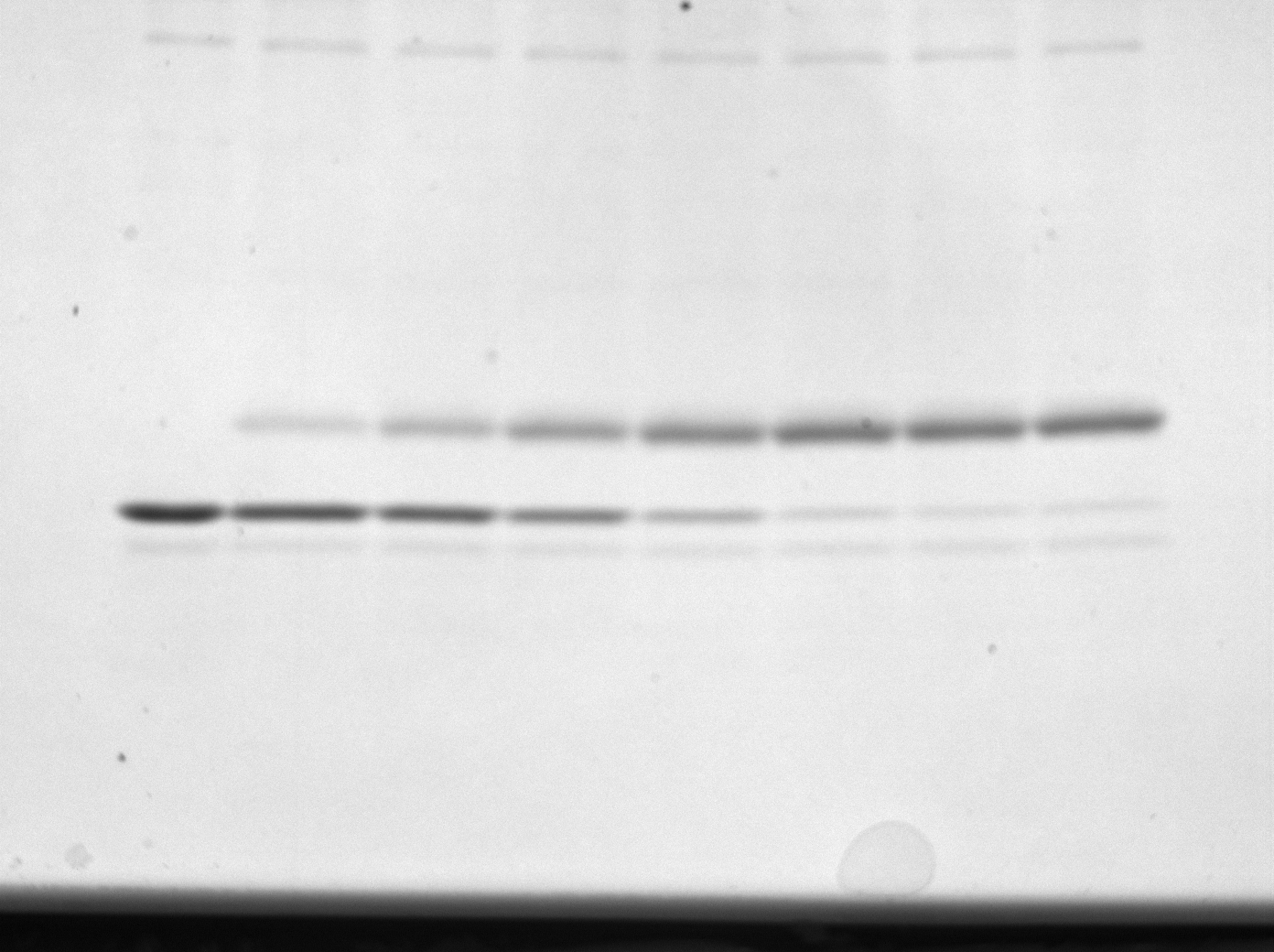

Supplement: Supplementary file 2 [file DataSheet2.zip › Supplement_2a_AGPGA/AGPGA X1 Loop 3/Ax1_L3_3.tif]

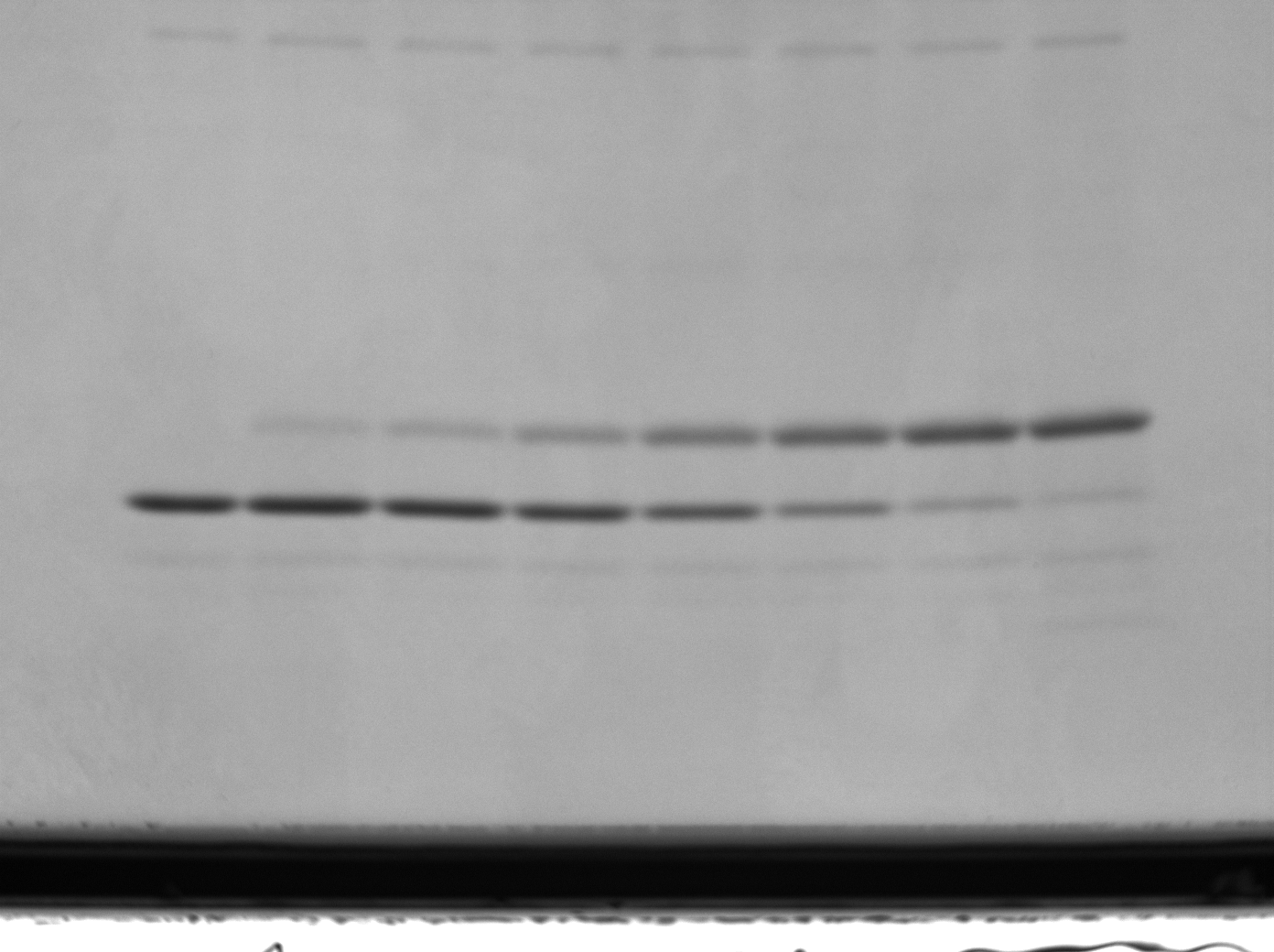

Supplement: Supplementary file 2 [file DataSheet2.zip › Supplement_2a_AGPGA/AGPGA X2 Loop 2/Ax2_L2_1.tif]

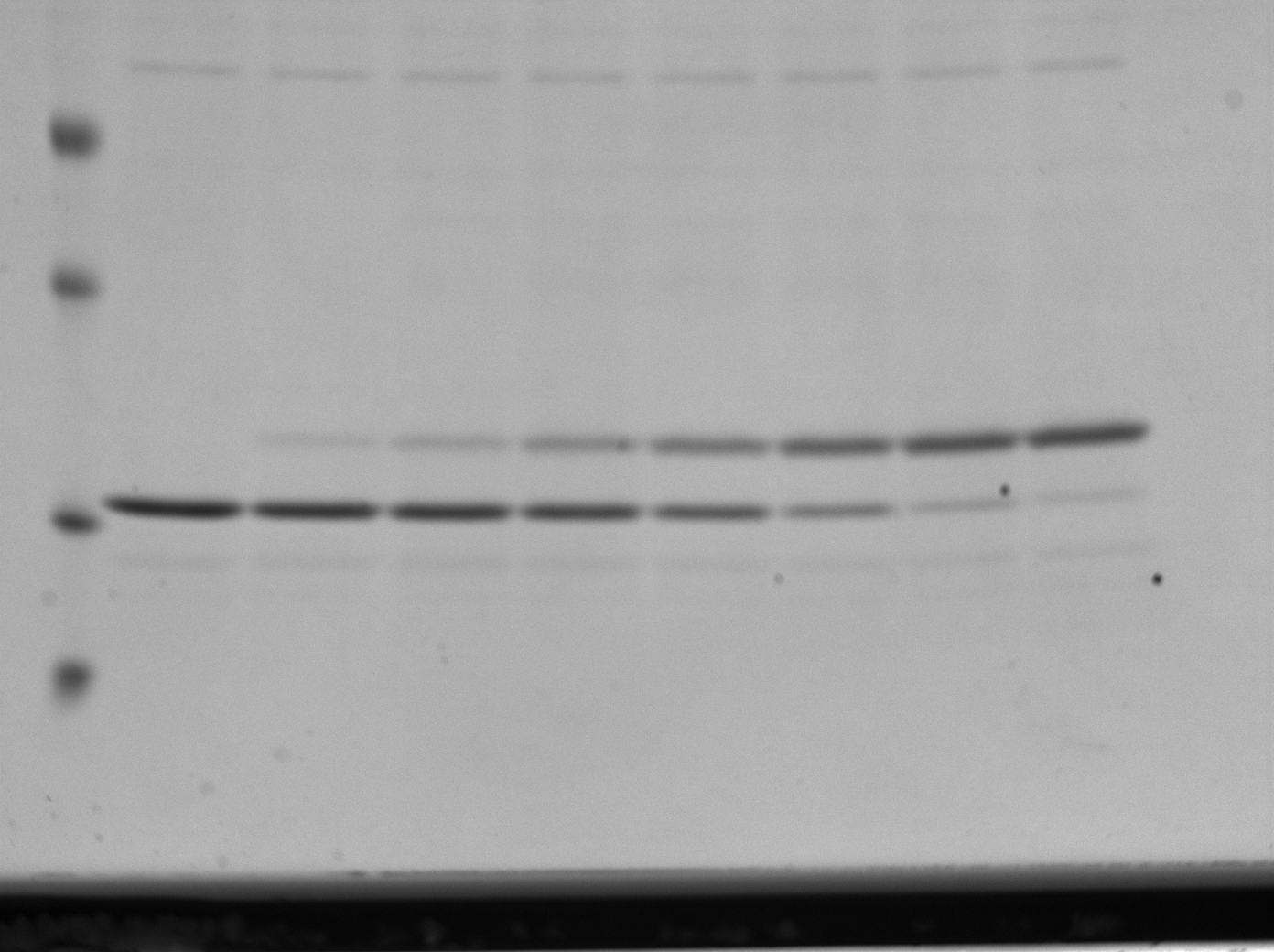

Supplement: Supplementary file 2 [file DataSheet2.zip › Supplement_2a_AGPGA/AGPGA X2 Loop 2/Ax2_L2_2.tif]

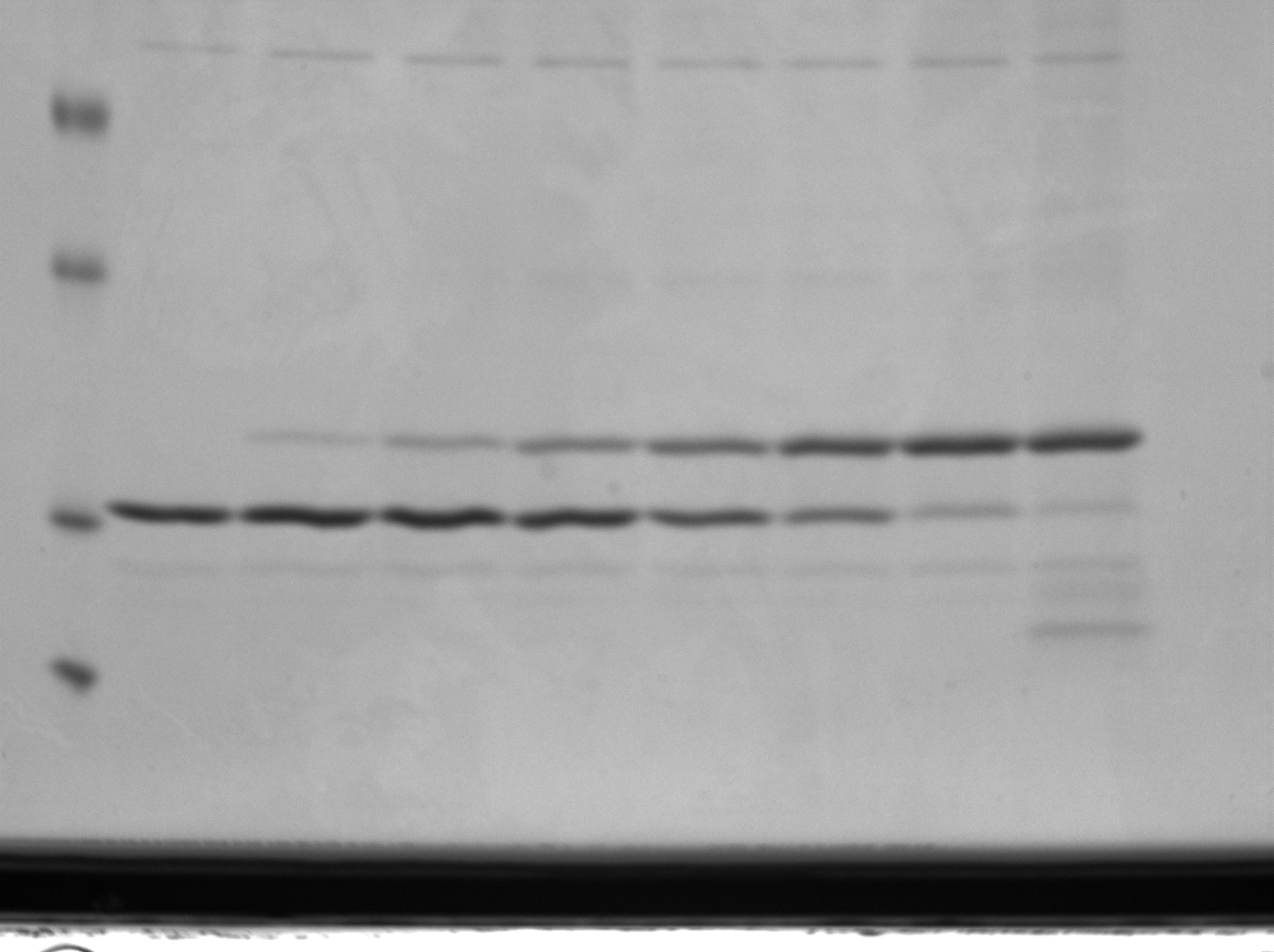

Supplement: Supplementary file 2 [file DataSheet2.zip › Supplement_2a_AGPGA/AGPGA X2 Loop 2/Ax2_L2_3.tif]

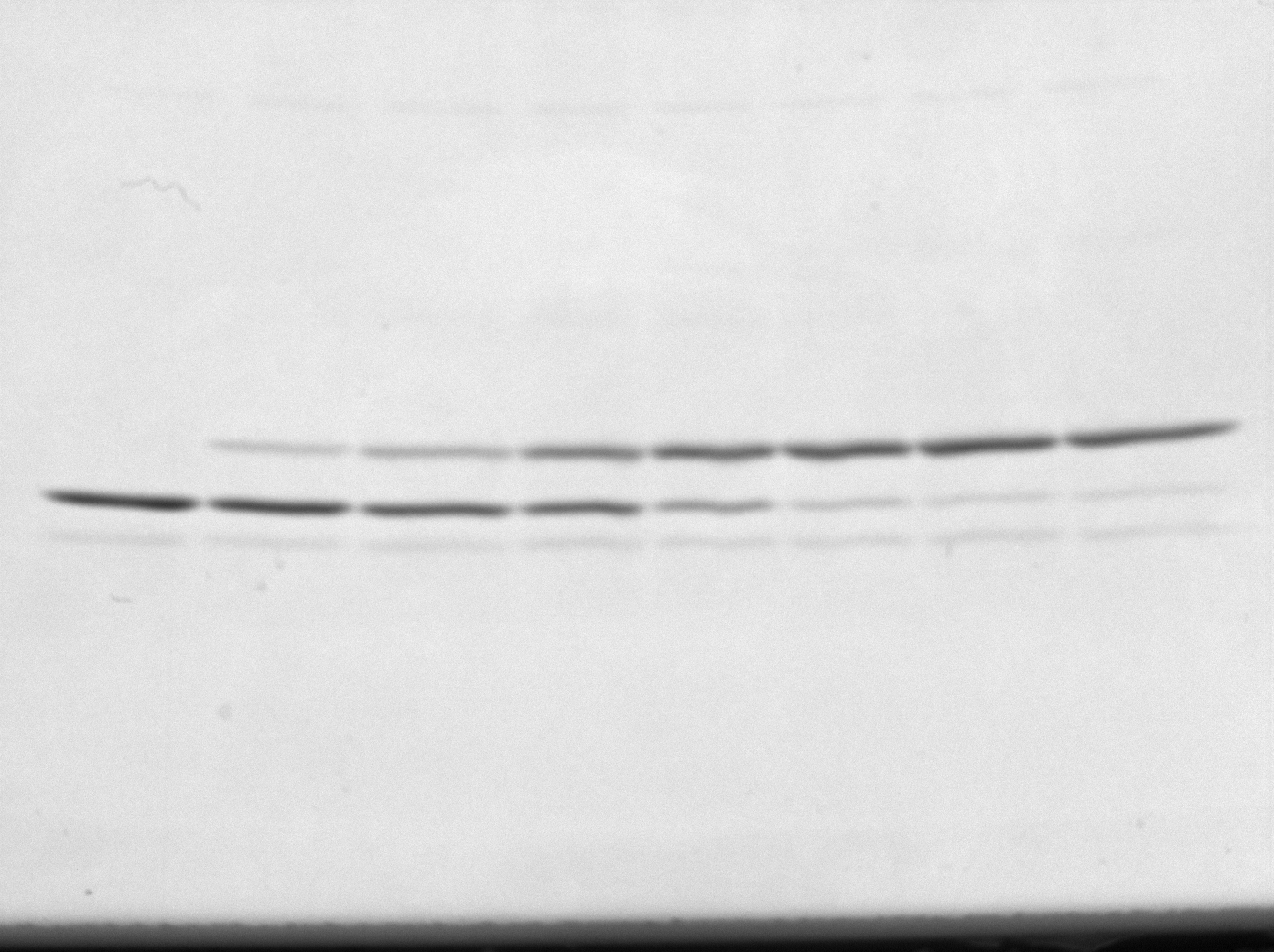

Supplement: Supplementary file 2 [file DataSheet2.zip › Supplement_2a_AGPGA/AGPGA X2 Loop 3/Ax2_L3_1.tif]

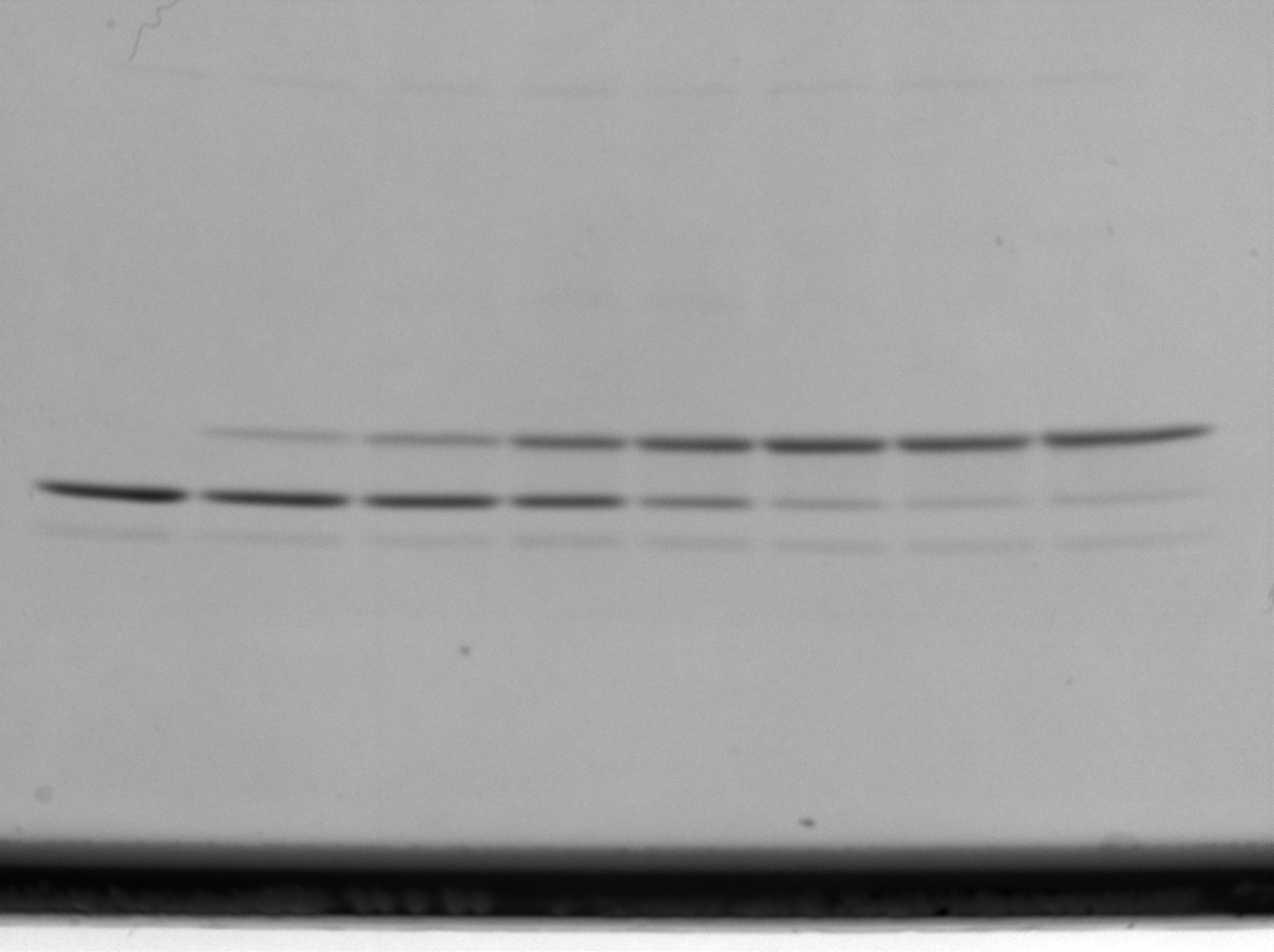

Supplement: Supplementary file 2 [file DataSheet2.zip › Supplement_2a_AGPGA/AGPGA X2 Loop 3/Ax2_L3_2.tif]

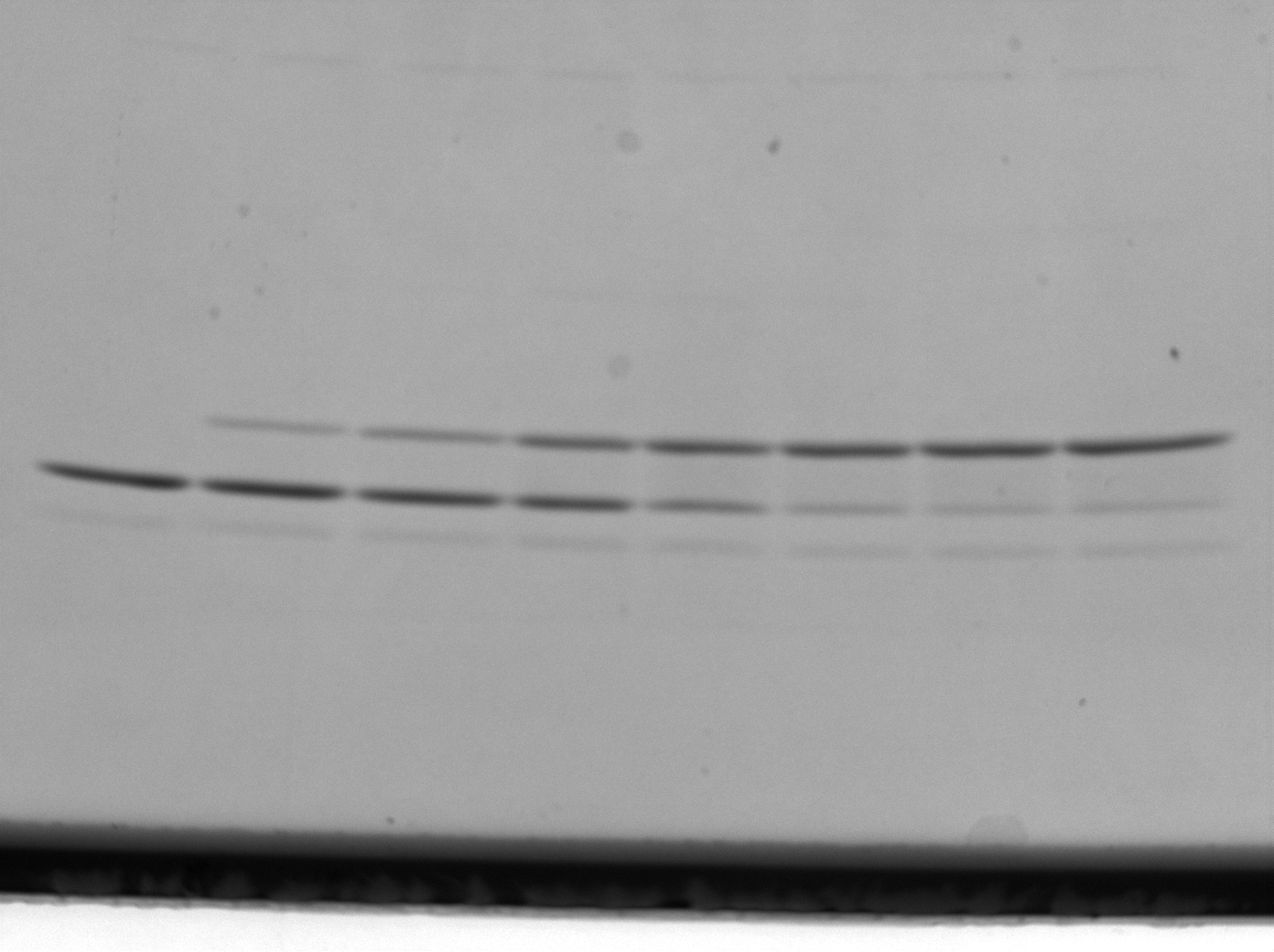

Supplement: Supplementary file 2 [file DataSheet2.zip › Supplement_2a_AGPGA/AGPGA X2 Loop 3/Ax2_L3_3.tif]

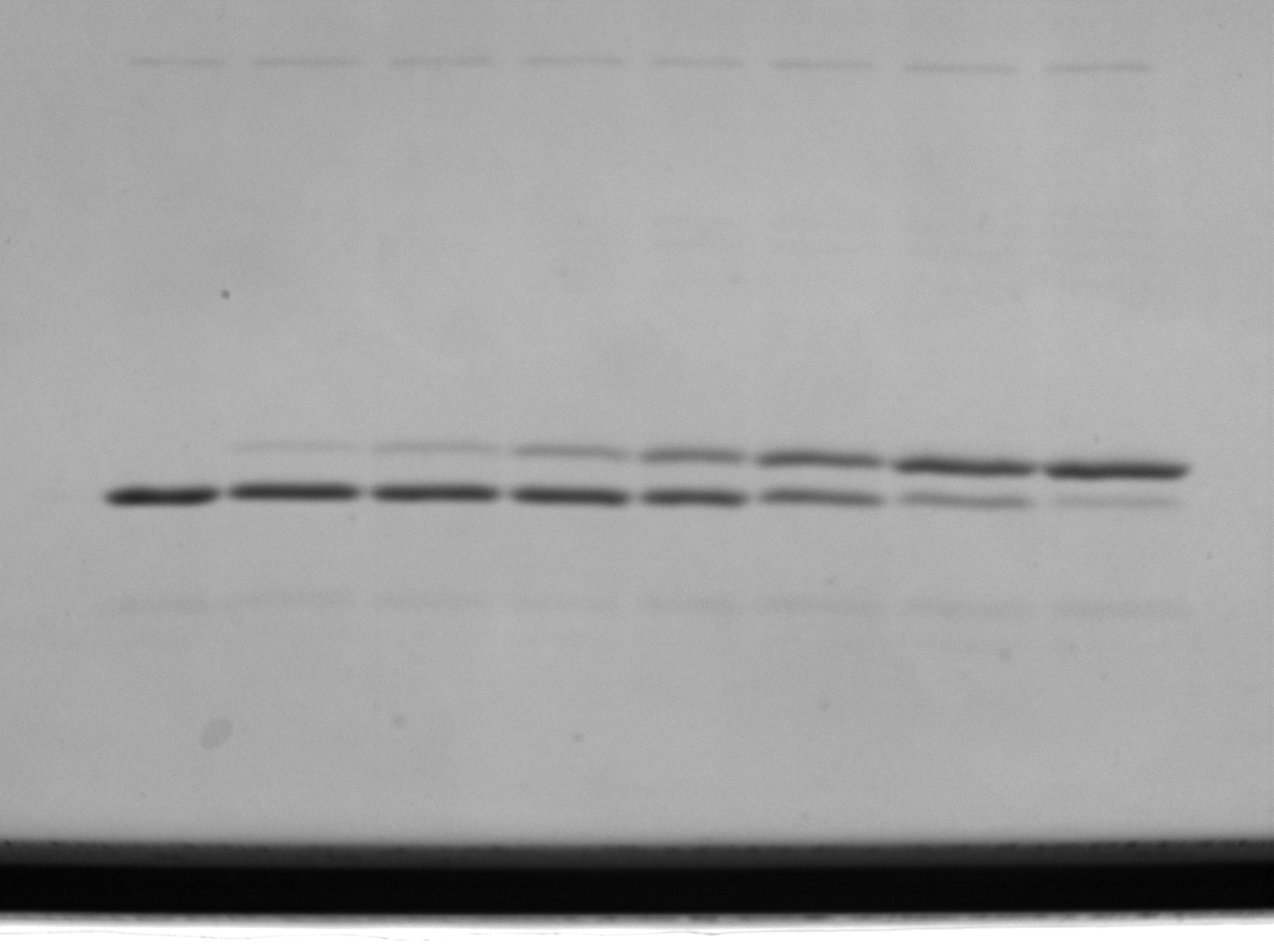

Supplement: Supplementary file 2 [file DataSheet2.zip › Supplement_2a_AGPGA/AGPGA X4 Loop 2/Ax4_L2_1.tif]

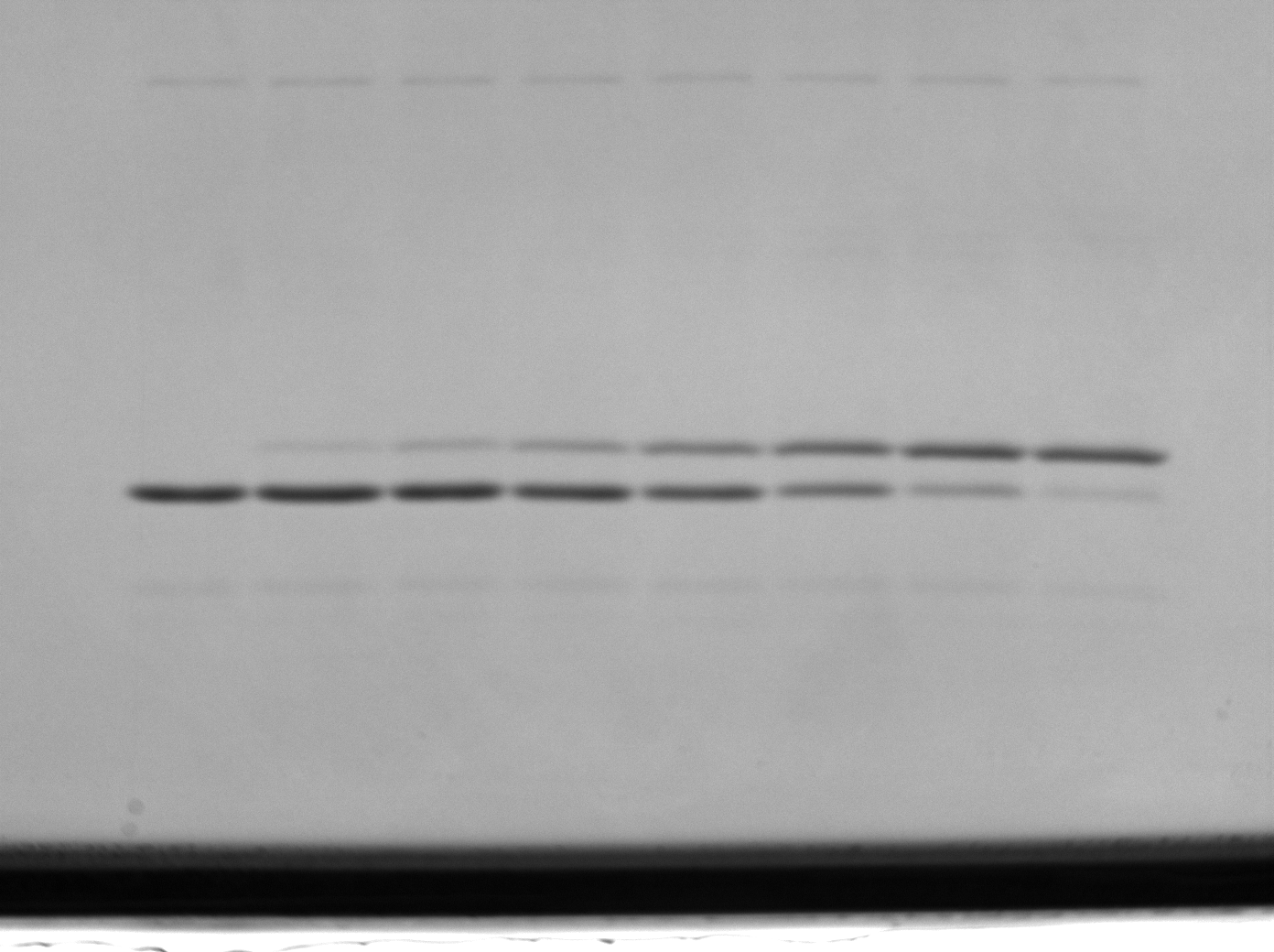

Supplement: Supplementary file 2 [file DataSheet2.zip › Supplement_2a_AGPGA/AGPGA X4 Loop 2/Ax4_L2_2.tif]

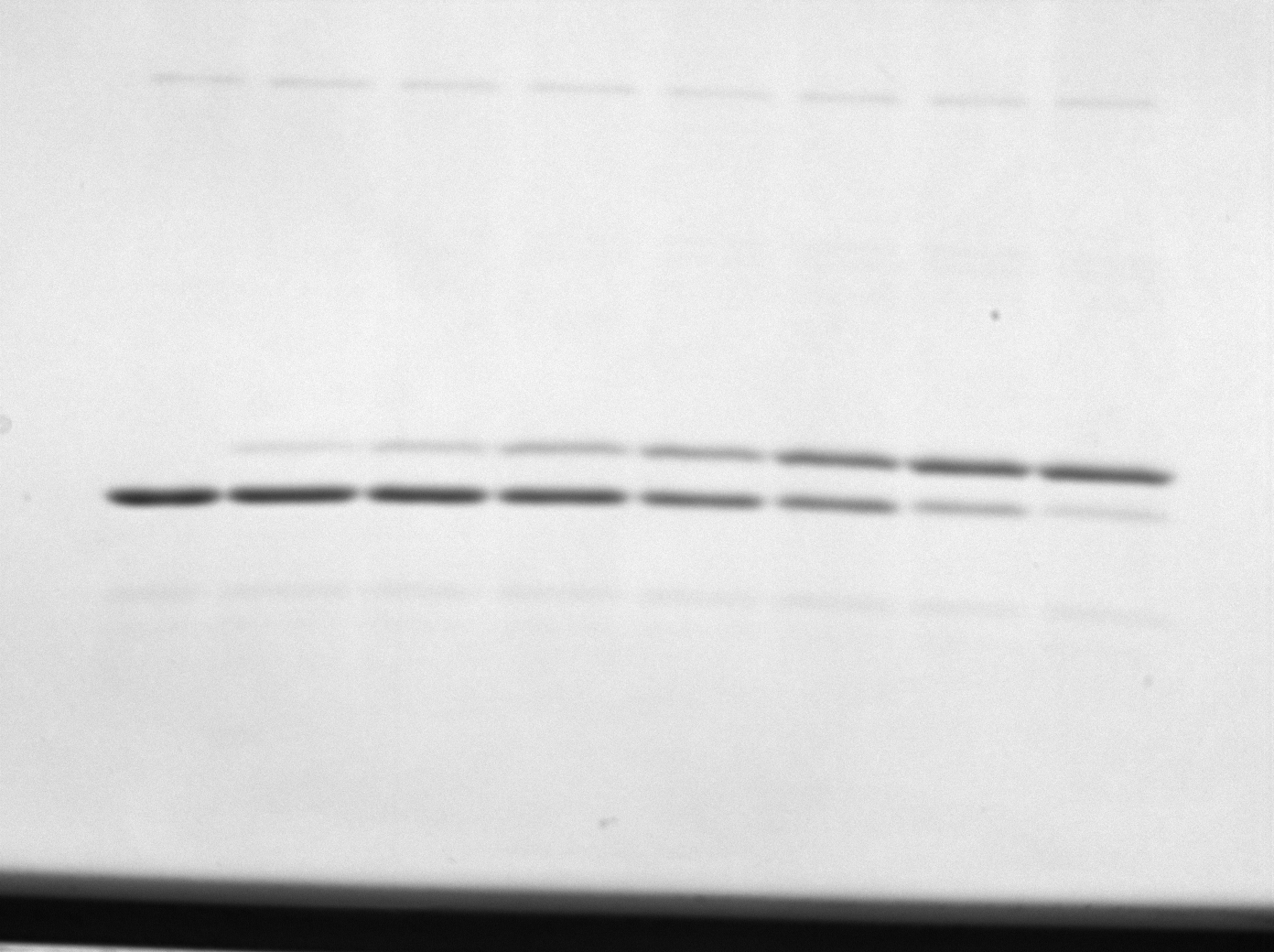

Supplement: Supplementary file 2 [file DataSheet2.zip › Supplement_2a_AGPGA/AGPGA X4 Loop 2/Ax4_L2_3.tif]

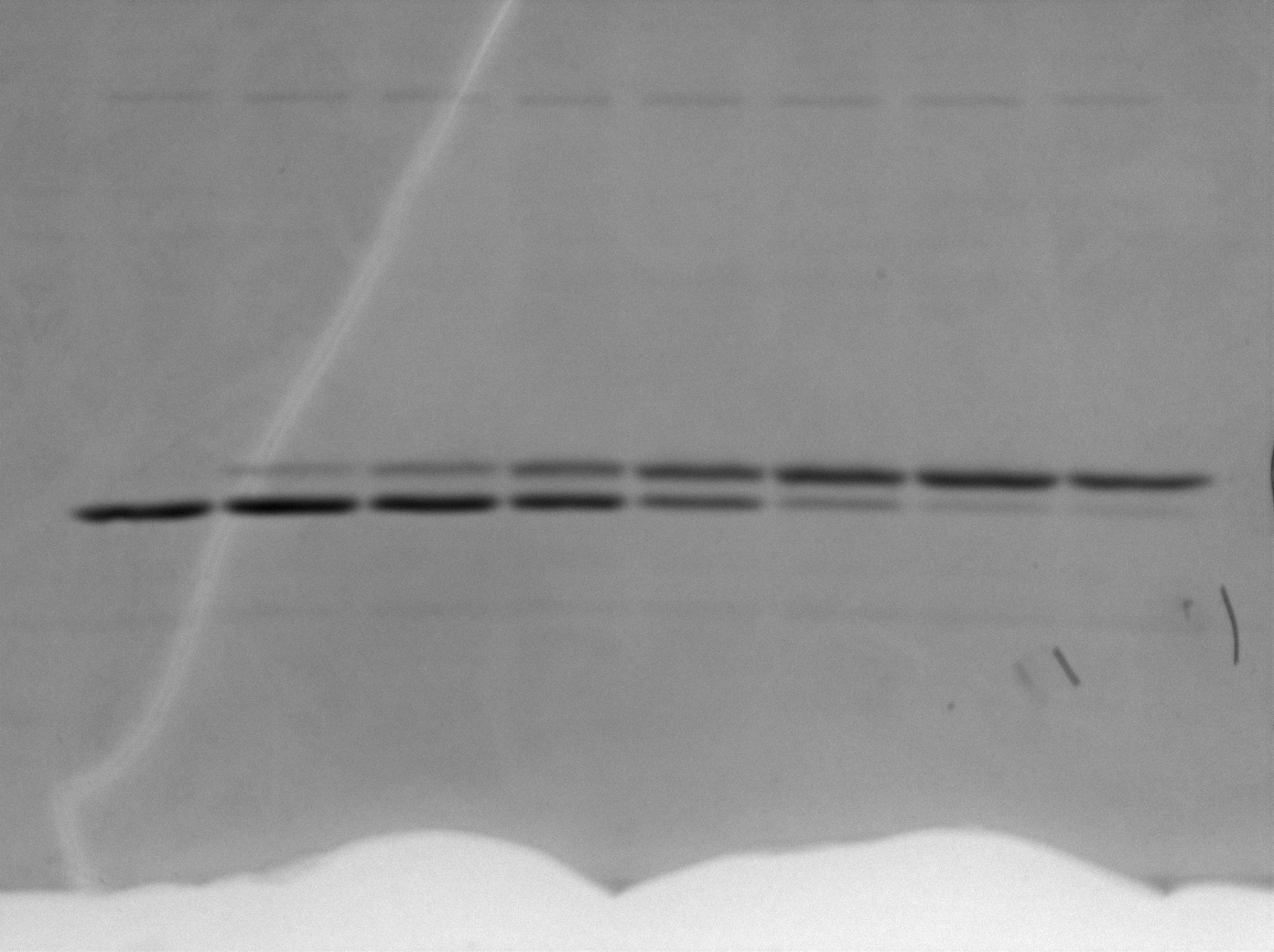

Supplement: Supplementary file 2 [file DataSheet2.zip › Supplement_2a_AGPGA/AGPGA X4 Loop 3/Ax4_L3_1.tif]

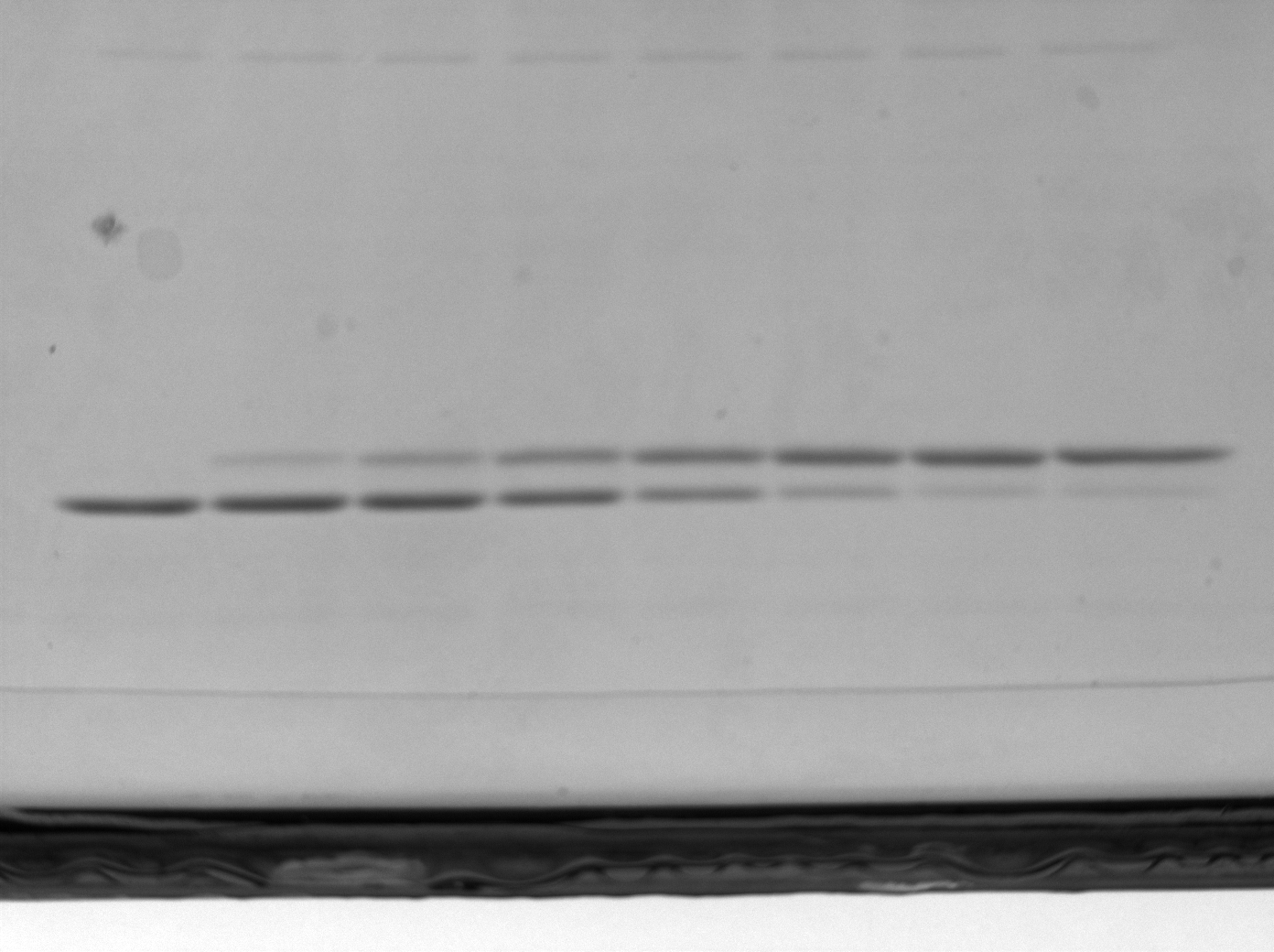

Supplement: Supplementary file 2 [file DataSheet2.zip › Supplement_2a_AGPGA/AGPGA X4 Loop 3/Ax4_L3_2.tif]

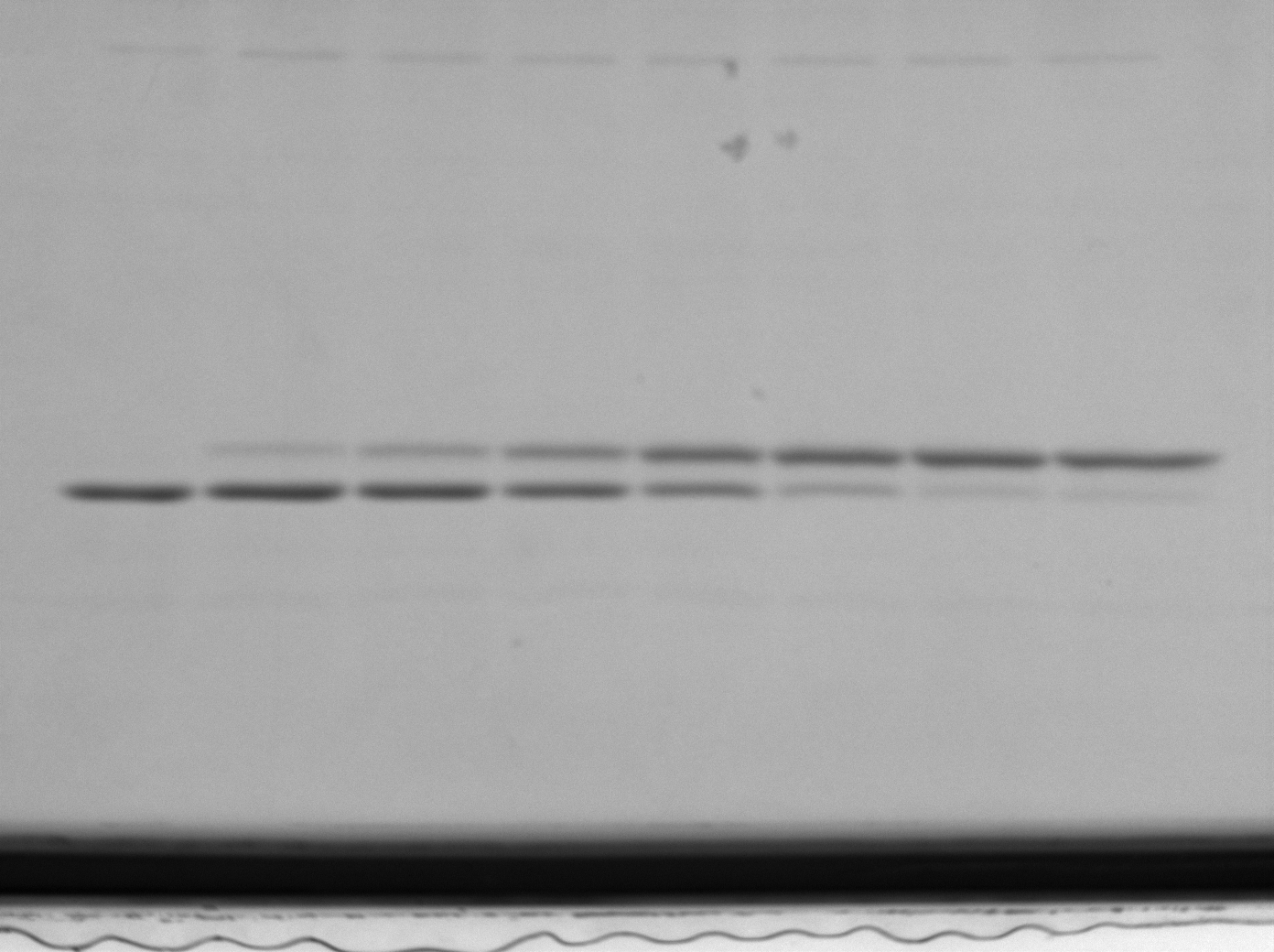

Supplement: Supplementary file 2 [file DataSheet2.zip › Supplement_2a_AGPGA/AGPGA X4 Loop 3/Ax4_L3_3.tif]

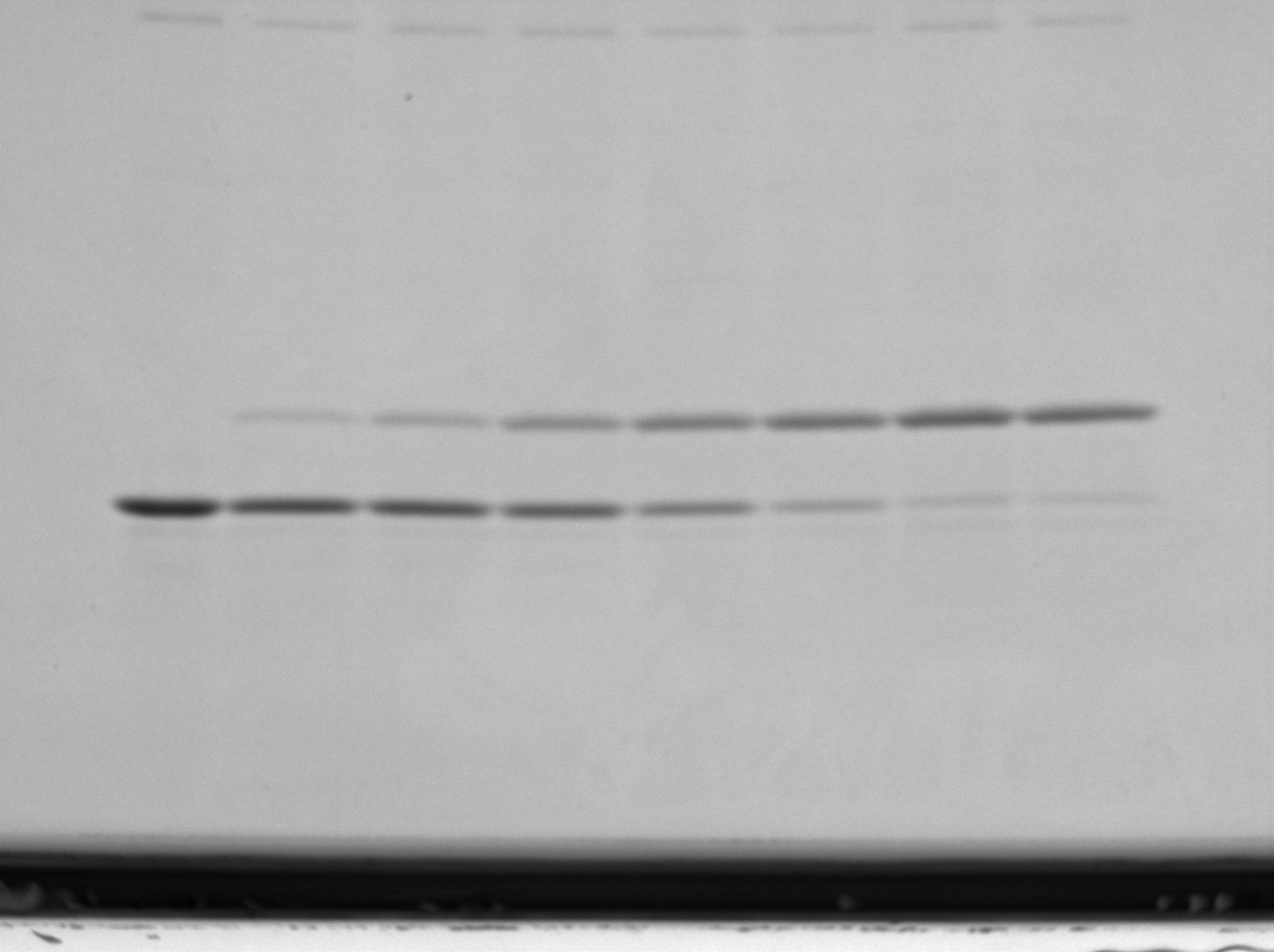

Supplement: Supplementary file 2 [file DataSheet2.zip › Supplement_2a_AGPGA/Wildtype/WT_1.tif]

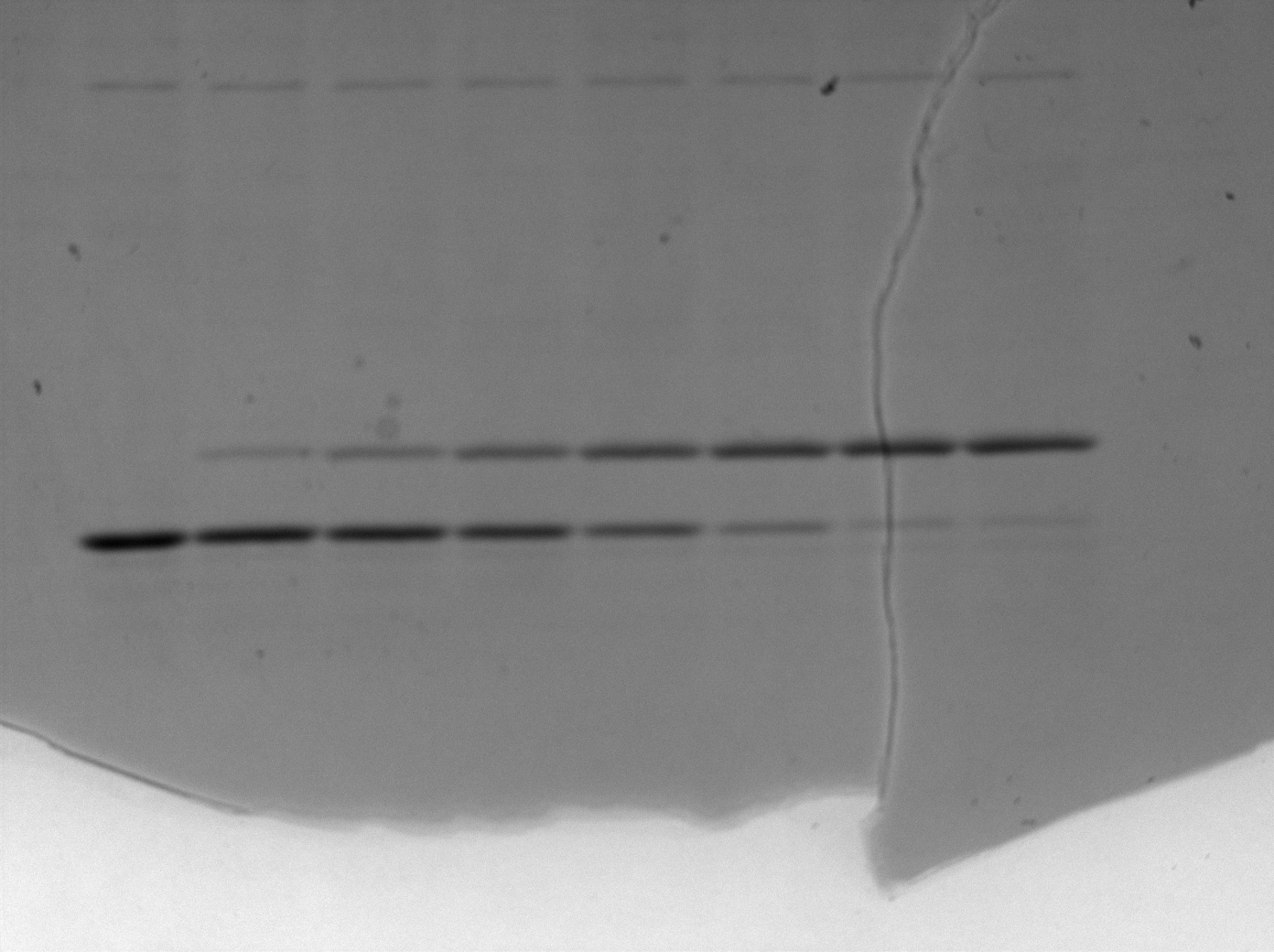

Supplement: Supplementary file 2 [file DataSheet2.zip › Supplement_2a_AGPGA/Wildtype/WT_2.tif]

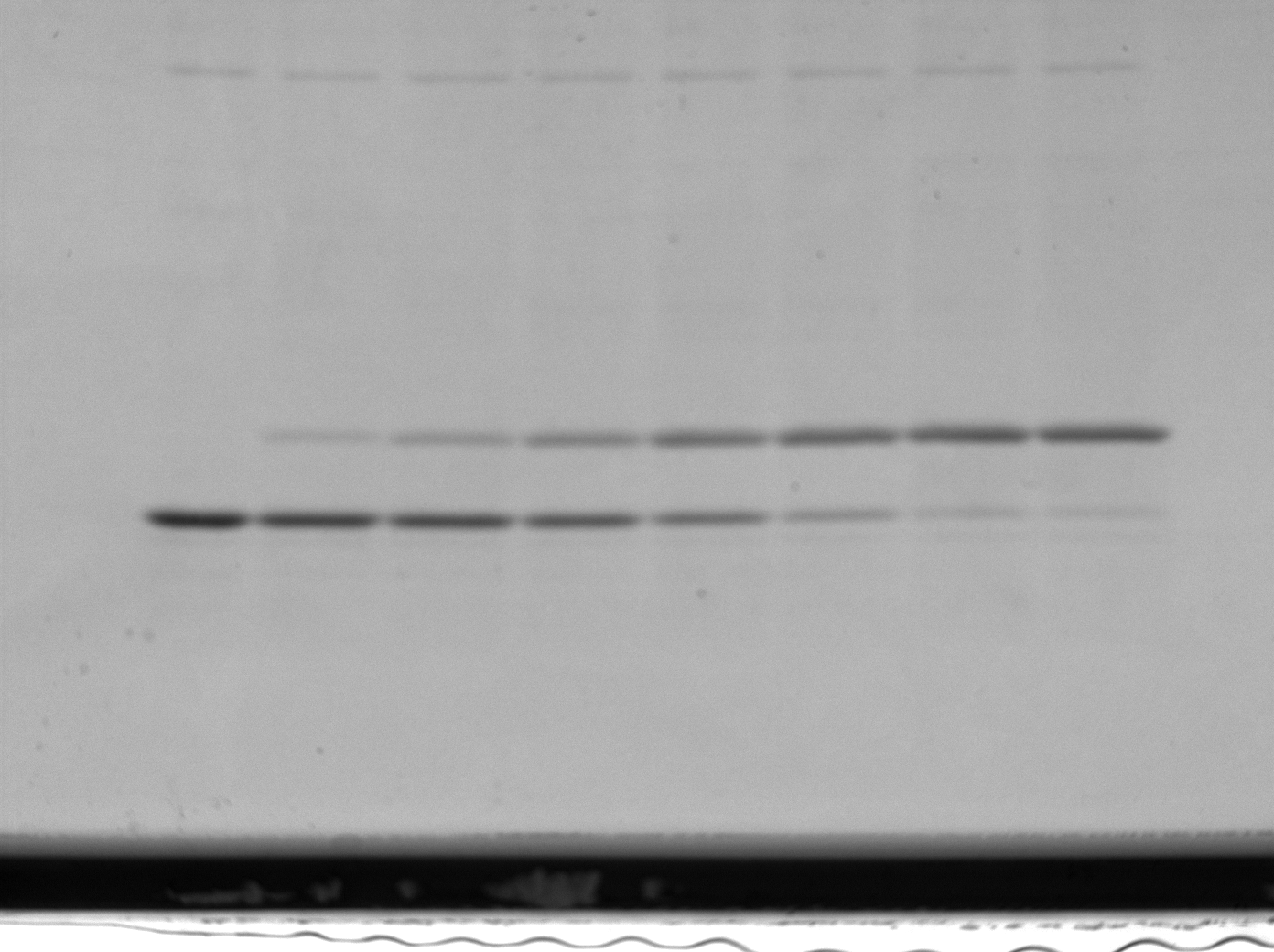

Supplement: Supplementary file 2 [file DataSheet2.zip › Supplement_2a_AGPGA/Wildtype/WT_3.tif]

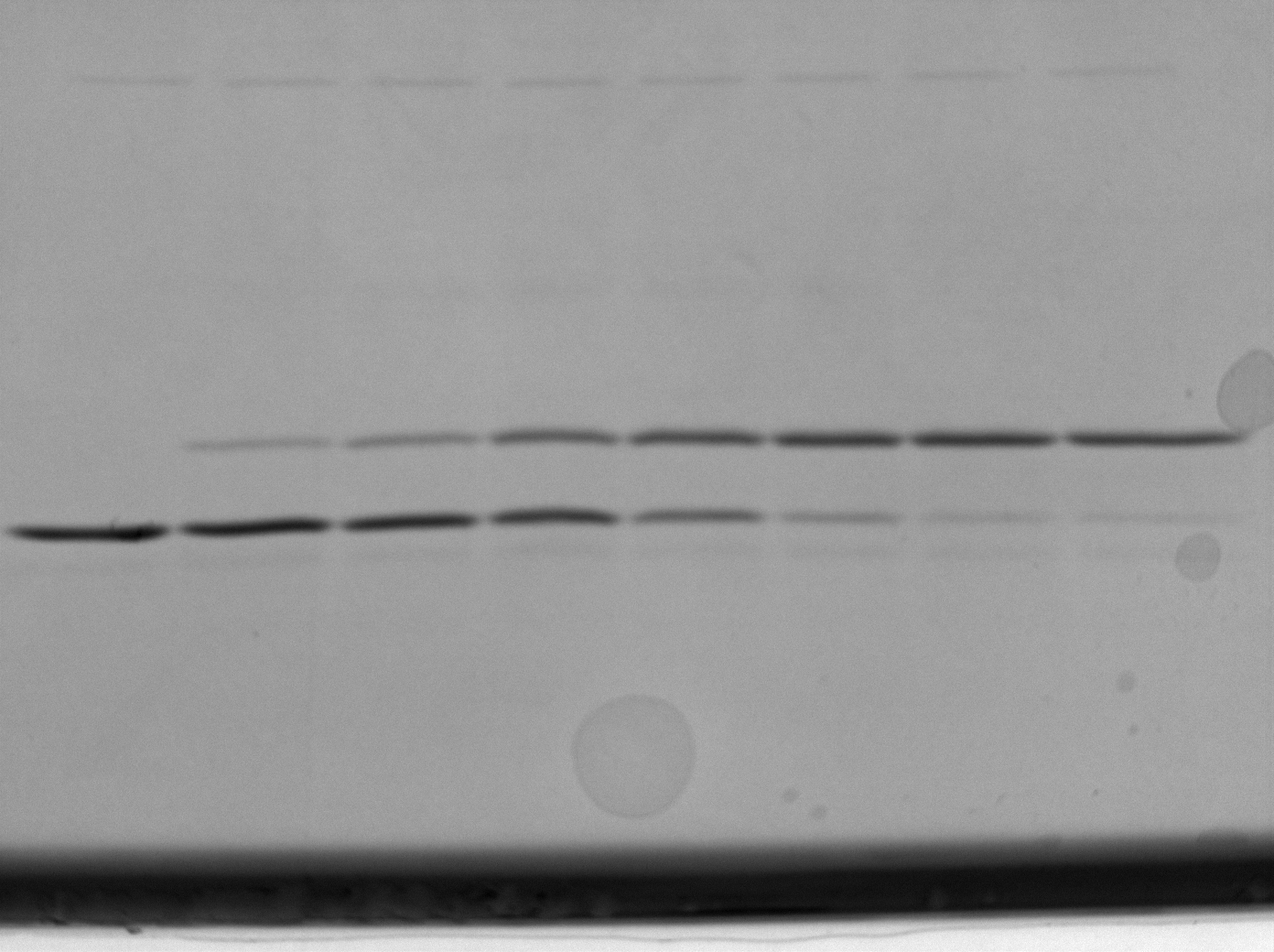

Supplement: Supplementary file 2 [file DataSheet2.zip › Supplement_2b_SPLAT/SPLAT X1 Loop 3/Sx1_L3_1.tif]

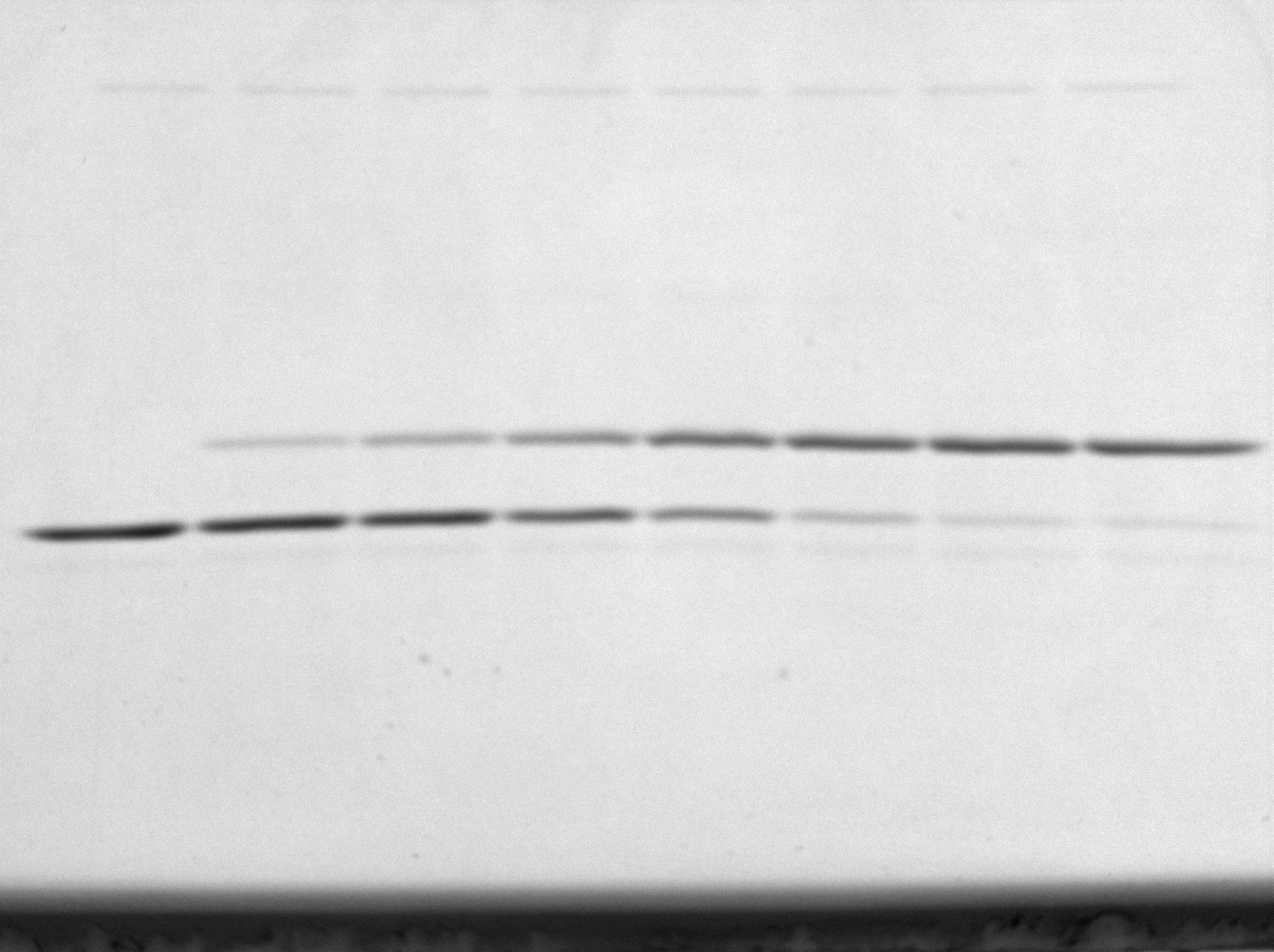

Supplement: Supplementary file 2 [file DataSheet2.zip › Supplement_2b_SPLAT/SPLAT X1 Loop 3/Sx1_L3_2.tif]

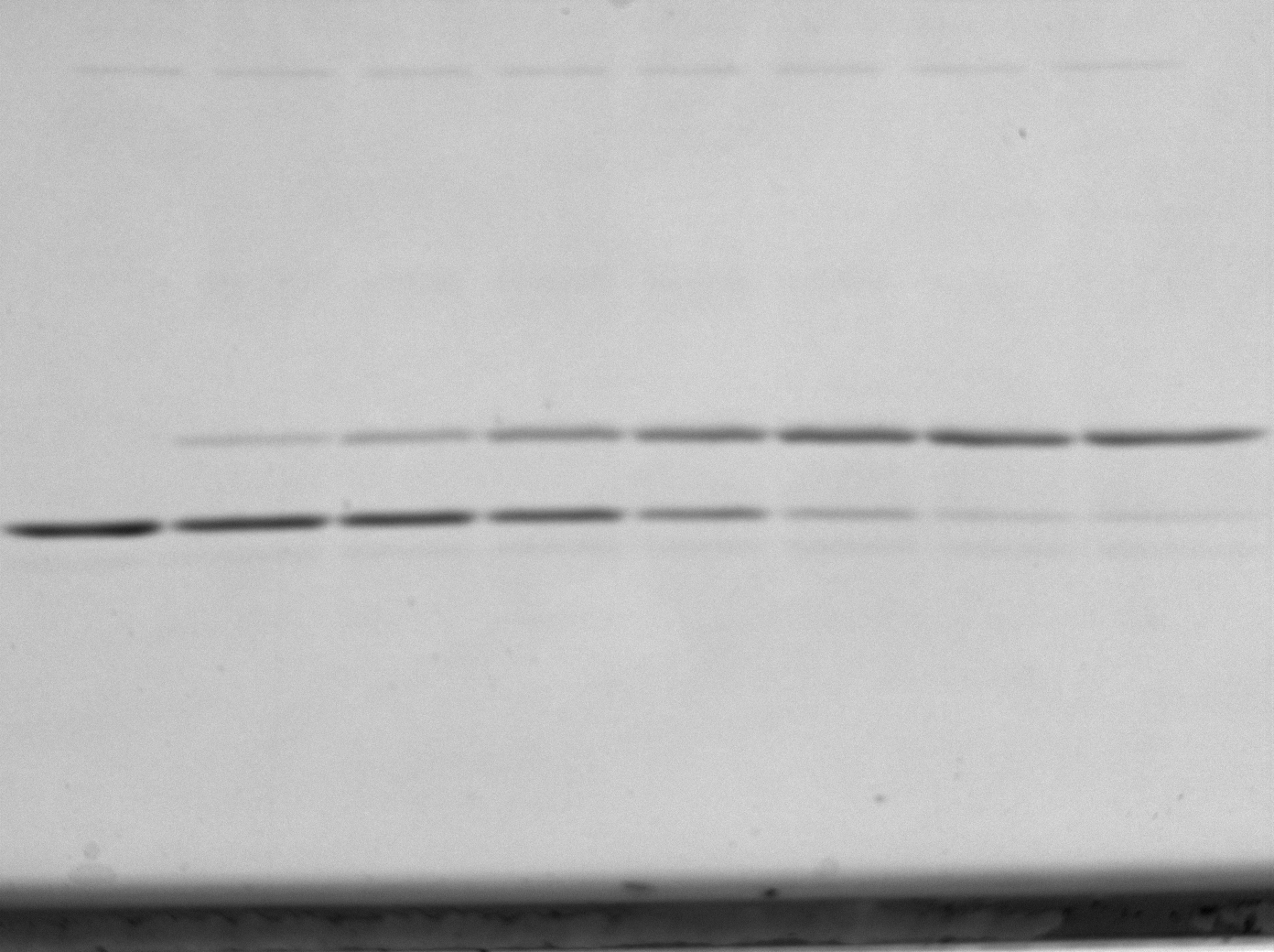

Supplement: Supplementary file 2 [file DataSheet2.zip › Supplement_2b_SPLAT/SPLAT X1 Loop 3/Sx1_L3_3.tif]

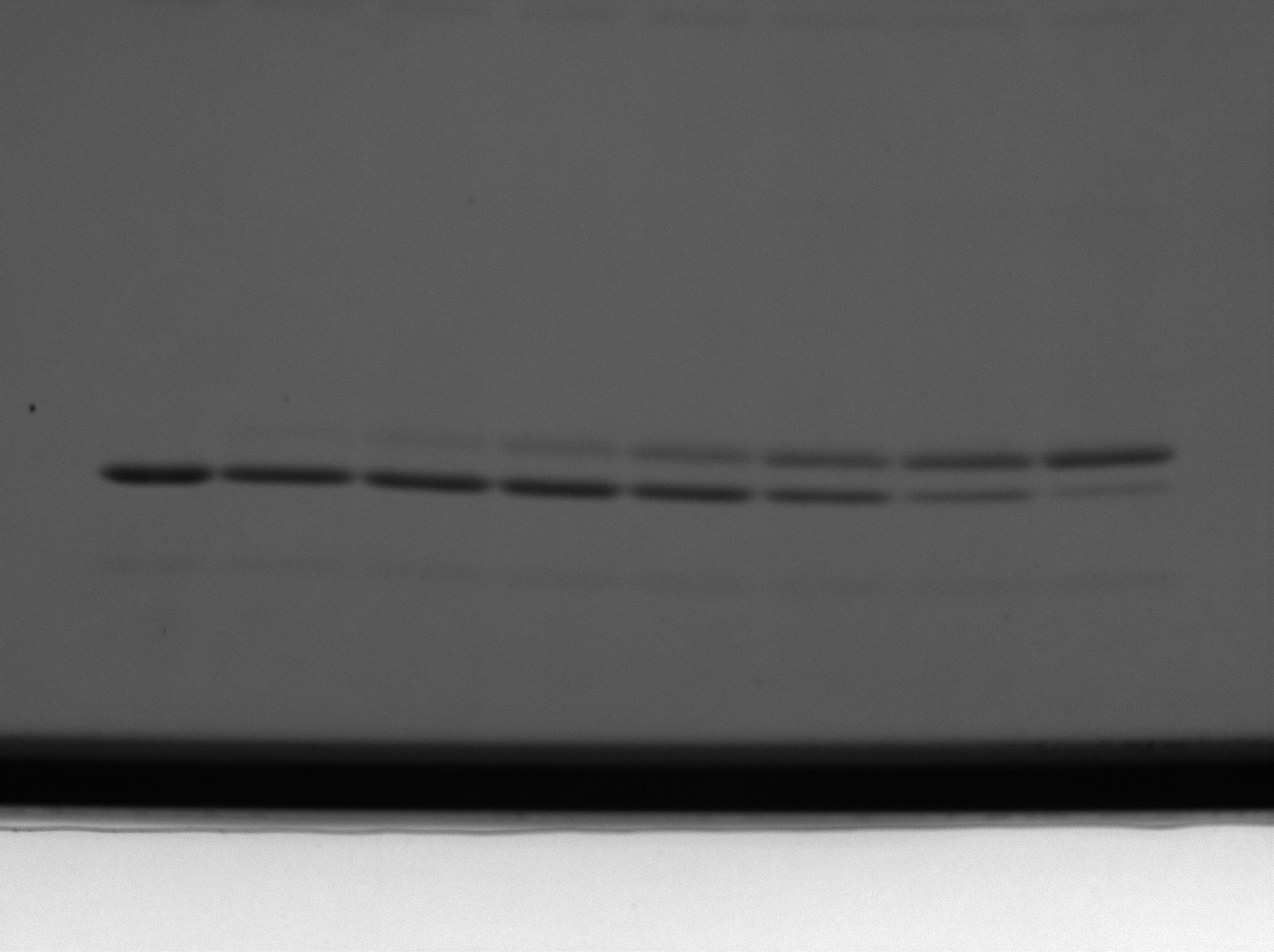

Supplement: Supplementary file 2 [file DataSheet2.zip › Supplement_2b_SPLAT/SPLAT X4 Loop 2 & Loop 3/Sx4_L2_1.tif]

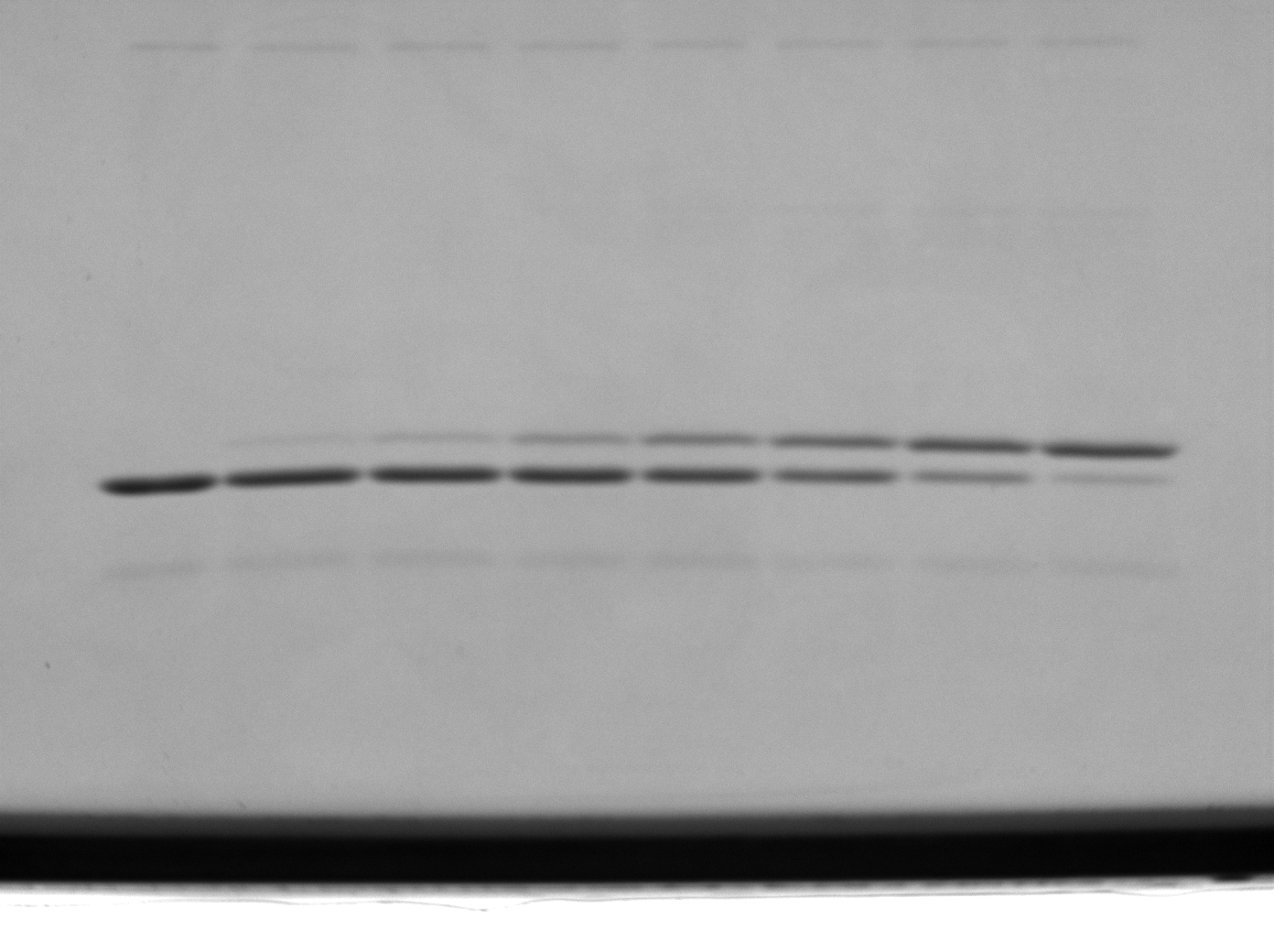

Supplement: Supplementary file 2 [file DataSheet2.zip › Supplement_2b_SPLAT/SPLAT X4 Loop 2 & Loop 3/Sx4_L2_2.tif]

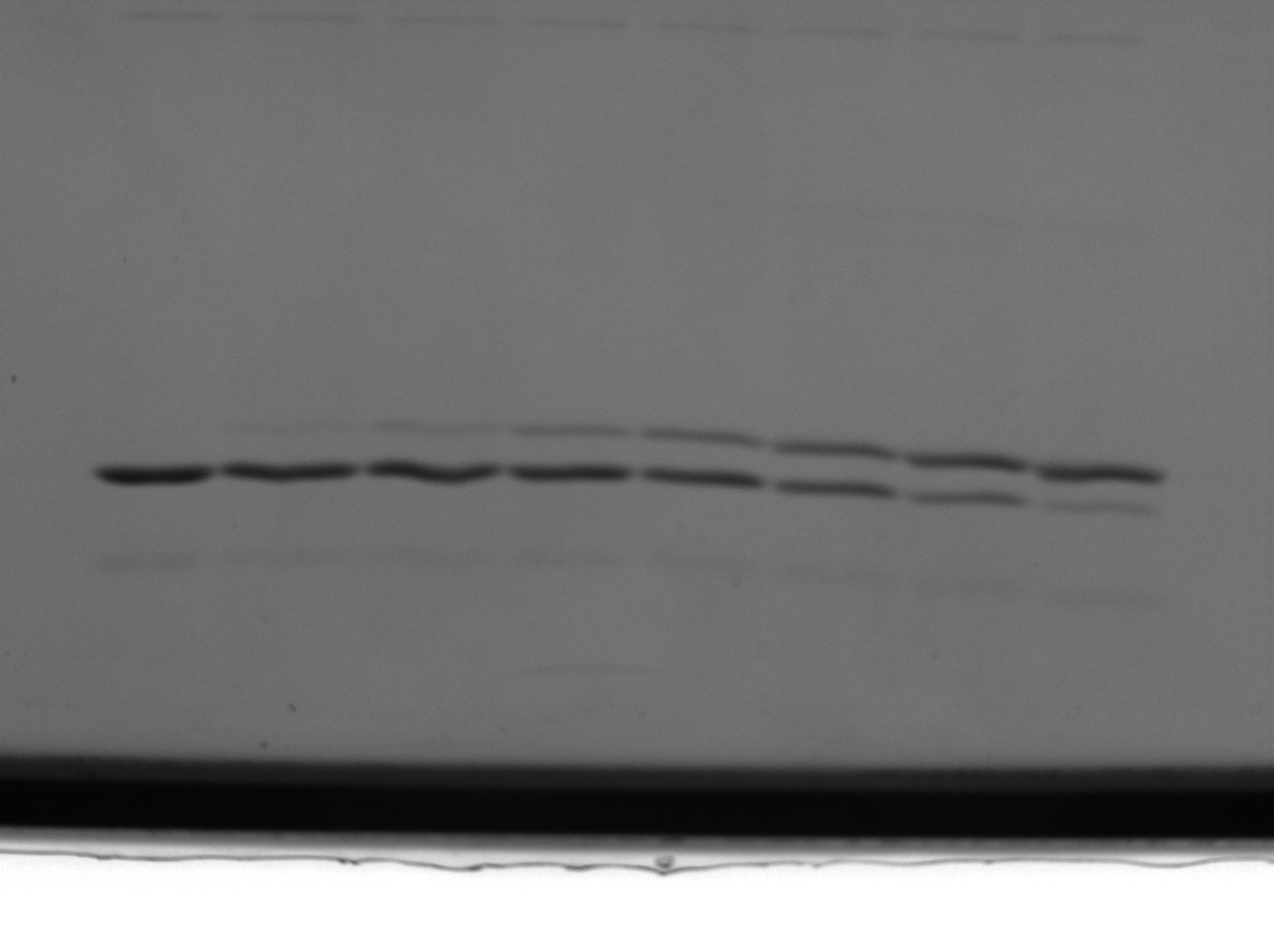

Supplement: Supplementary file 2 [file DataSheet2.zip › Supplement_2b_SPLAT/SPLAT X4 Loop 2 & Loop 3/Sx4_L2_3.tif]

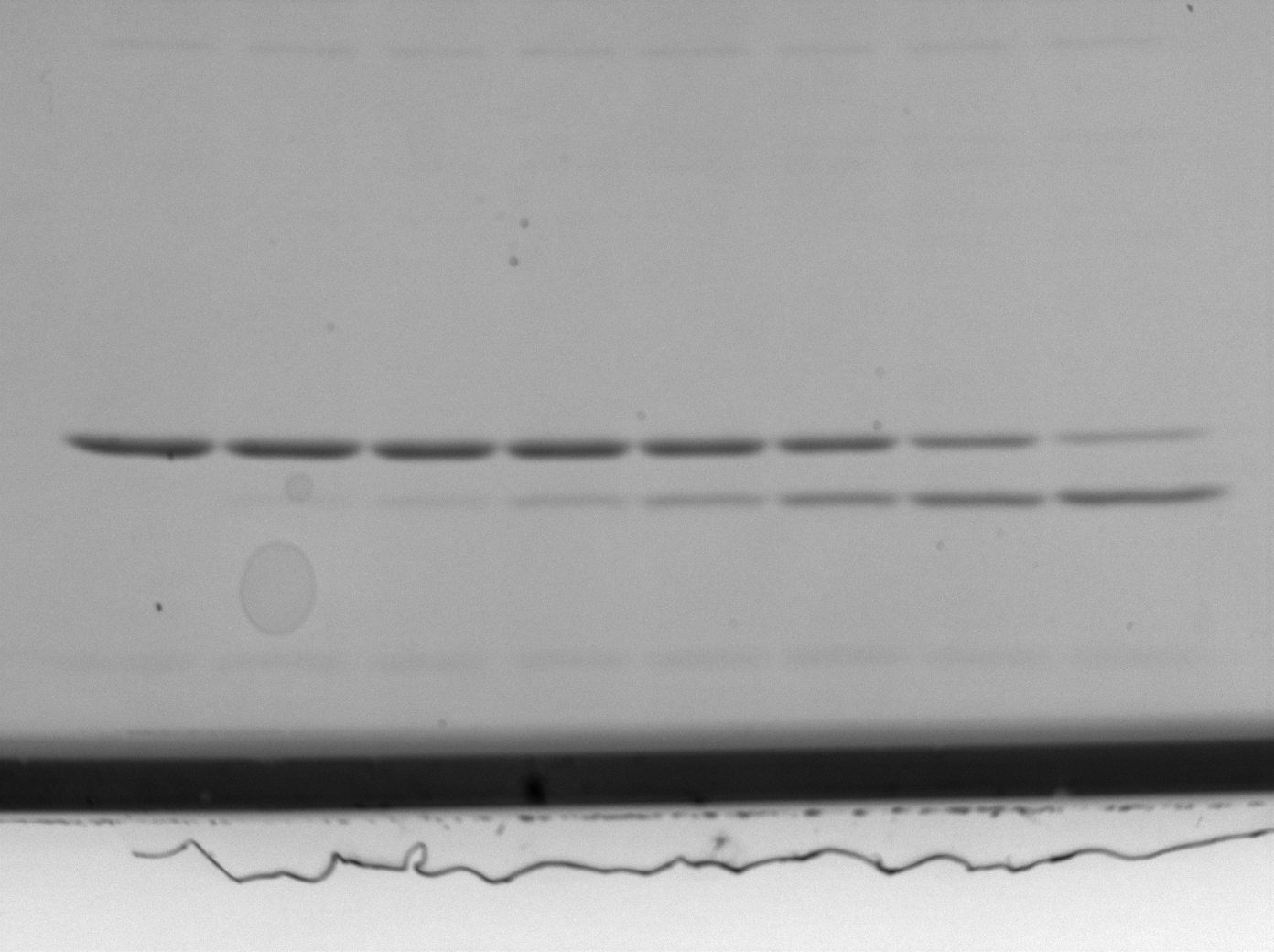

Supplement: Supplementary file 2 [file DataSheet2.zip › Supplement_2b_SPLAT/SPLAT X4 Loop 2/Sx4_L2&L3_1.tif]

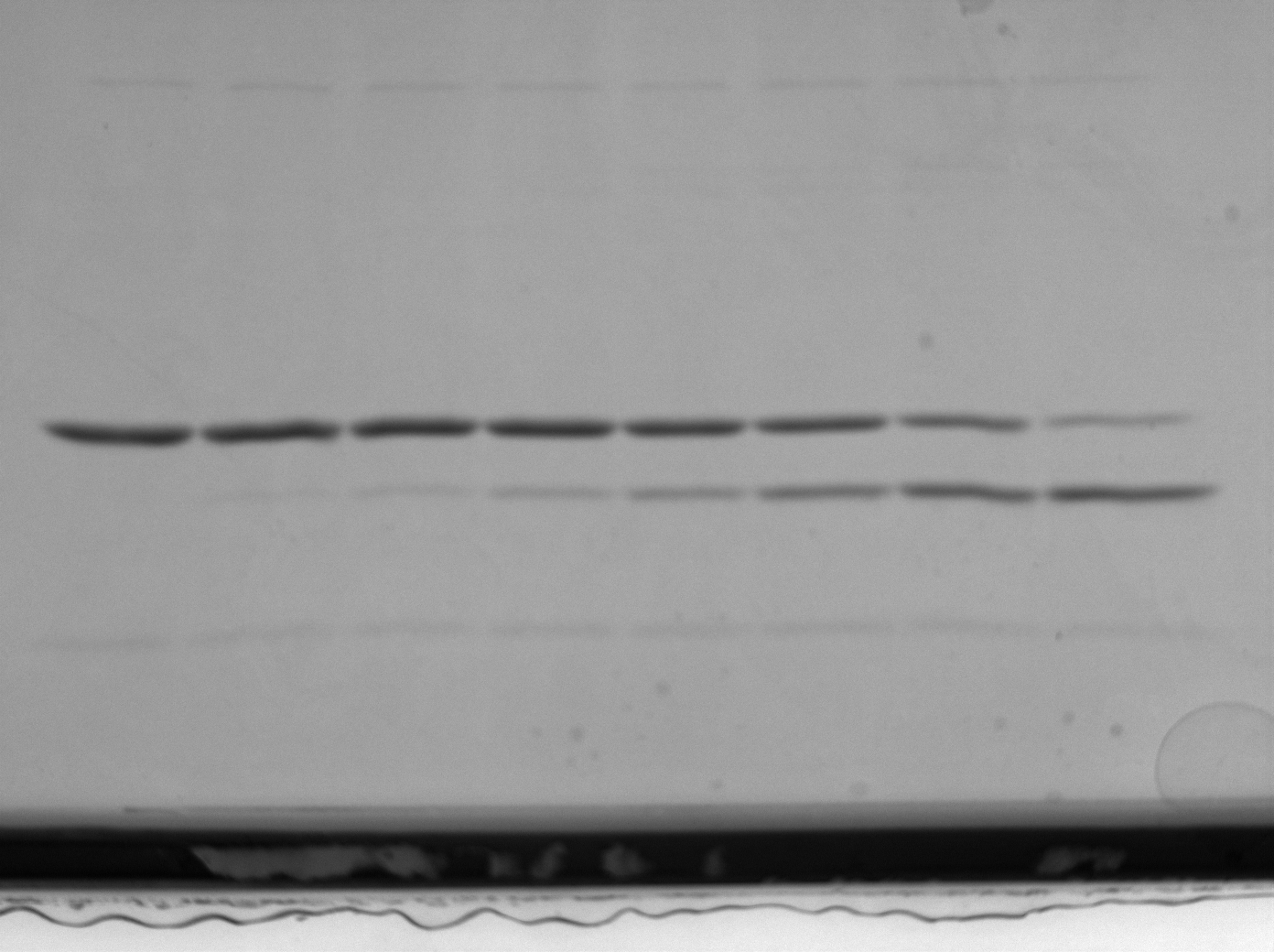

Supplement: Supplementary file 2 [file DataSheet2.zip › Supplement_2b_SPLAT/SPLAT X4 Loop 2/Sx4_L2&L3_2.tif]

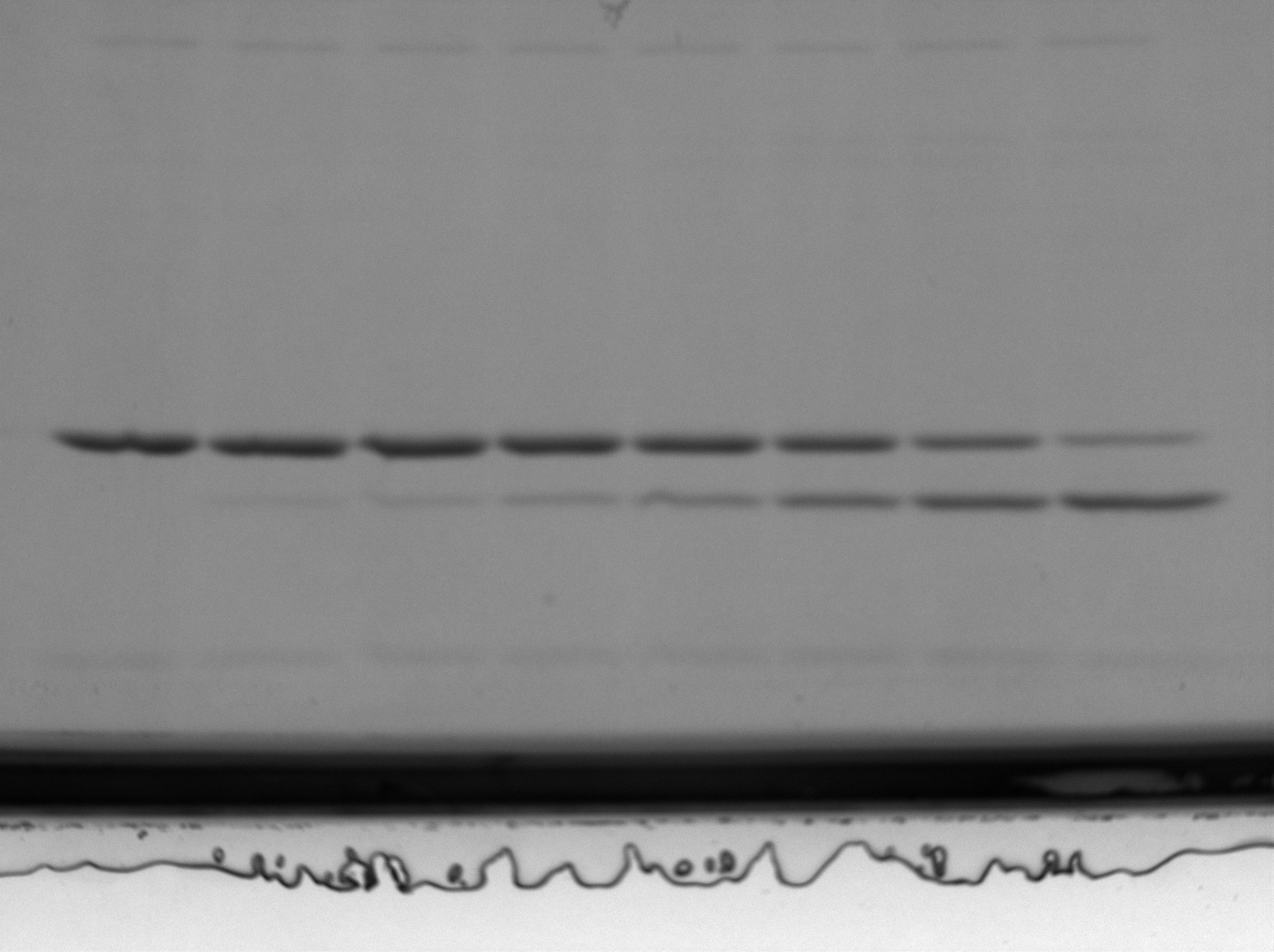

Supplement: Supplementary file 2 [file DataSheet2.zip › Supplement_2b_SPLAT/SPLAT X4 Loop 2/Sx4_L2&L3_3.tif]

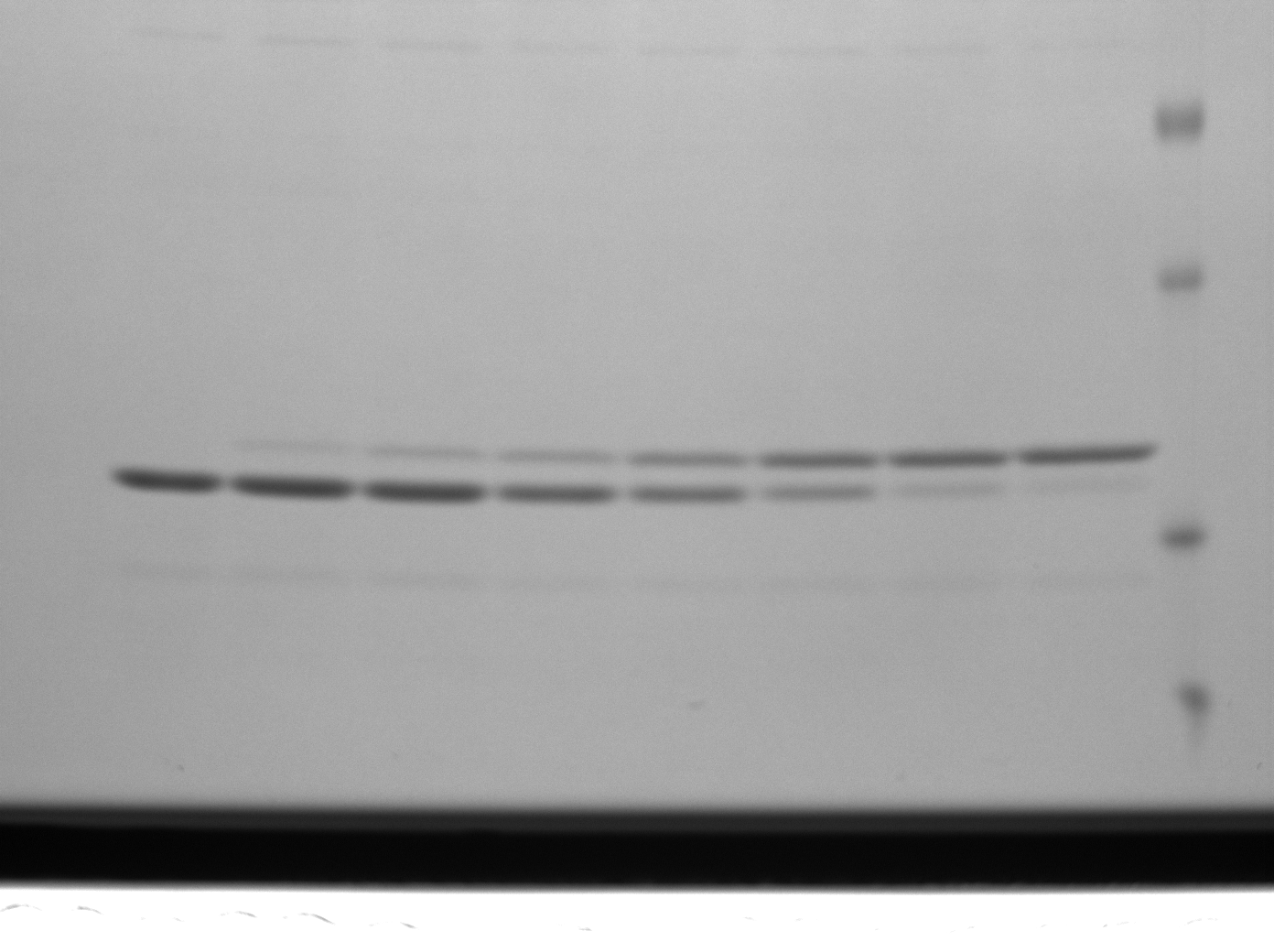

Supplement: Supplementary file 2 [file DataSheet2.zip › Supplement_2b_SPLAT/SPLAT X4 Loop 3/Sx4_L3_1.tif]

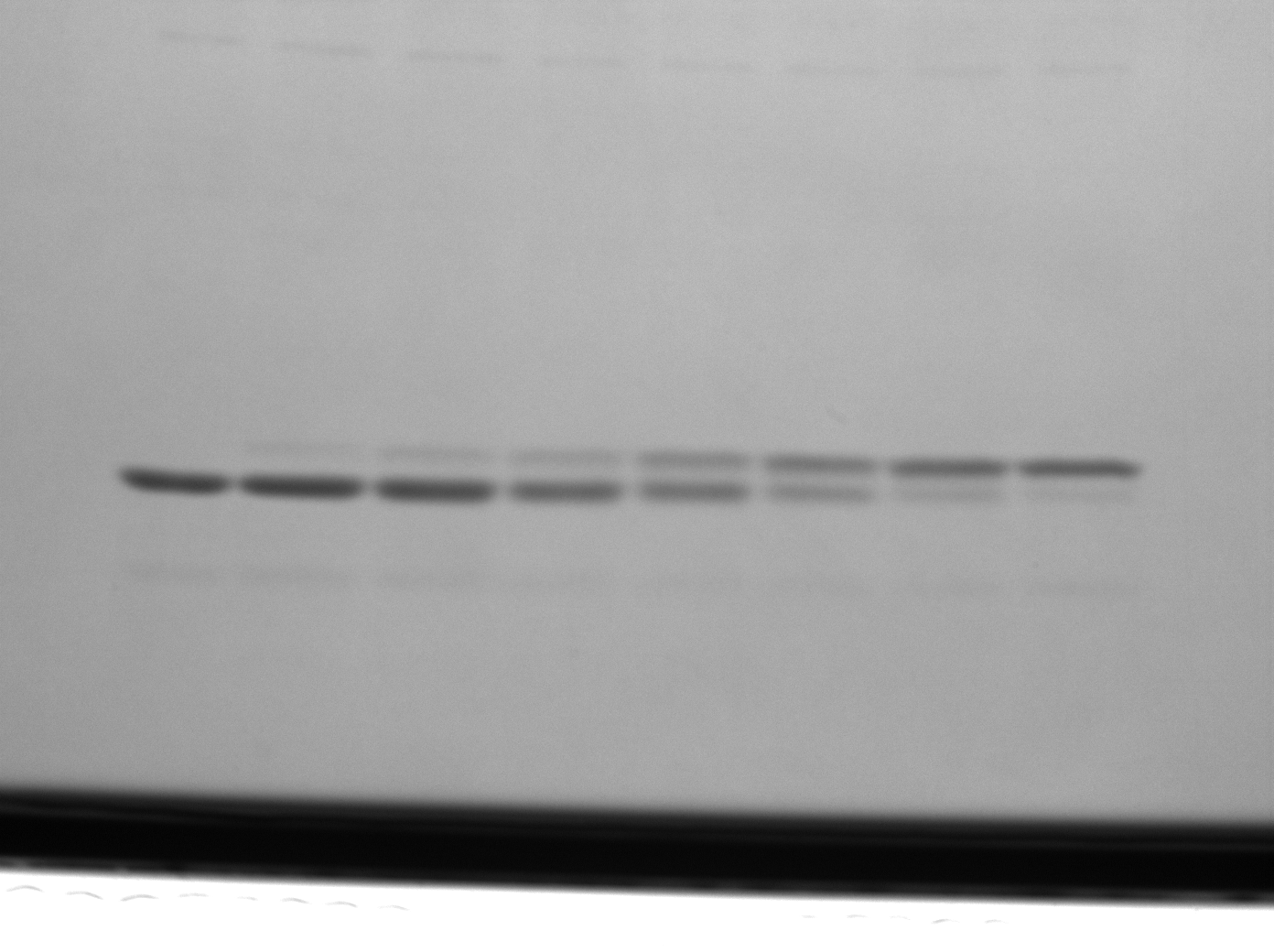

Supplement: Supplementary file 2 [file DataSheet2.zip › Supplement_2b_SPLAT/SPLAT X4 Loop 3/Sx4_L3_2.tif]

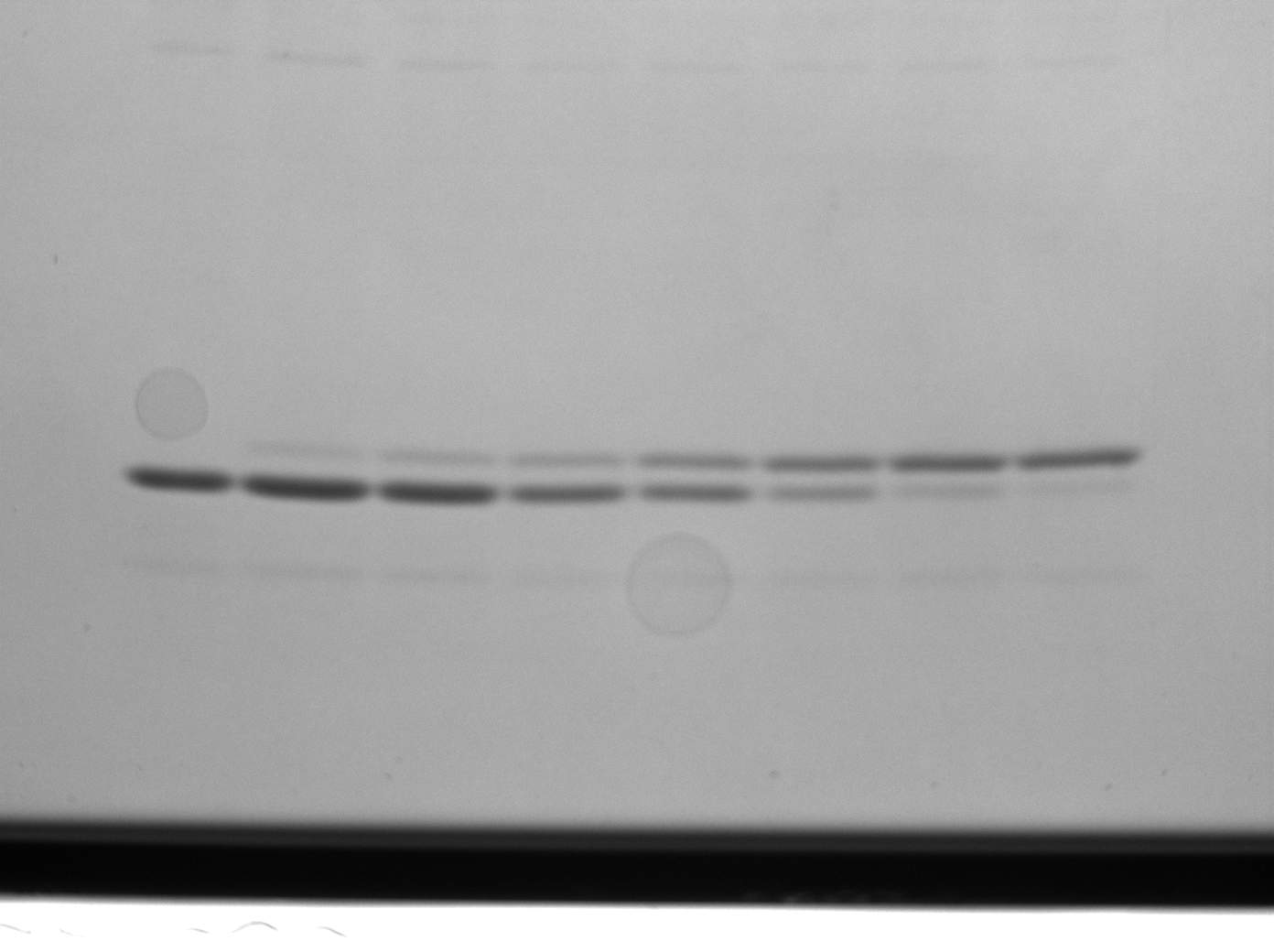

Supplement: Supplementary file 2 [file DataSheet2.zip › Supplement_2b_SPLAT/SPLAT X4 Loop 3/Sx4_L3_3.tif]
